# Determinants of trust in times of crises: A cross-sectional study of 3,065 German-speaking adults from the D-A-CH region

**DOI:** 10.1371/journal.pone.0286488

**Published:** 2023-10-12

**Authors:** Eva S. Schernhammer, Jakob Weitzer, Emilie Han, Martin Bertau, Lukas Zenk, Guido Caniglia, Manfred D. Laubichler, Brenda M. Birmann, Gerald Steiner

**Affiliations:** 1 Department of Epidemiology, Center for Public Health, Medical University of Vienna, Vienna, Austria; 2 Channing Division of Network Medicine, Department of Medicine, Brigham and Women’s Hospital and Harvard Medical School, Boston, MA, United States of America; 3 Complexity Science Hub Vienna, Vienna, Austria; 4 Department of Health Promotion and Prevention, Federal Ministry of the Republic of Austria for Social Affairs, Health, Care and Consumer Protection, Vienna, Austria; 5 Institute of Chemical Technology, Freiberg University of Mining and Technology, Freiberg, Germany; 6 Fraunhofer Institut für Keramische Technologien und Systeme IKTS, Fraunhofer Technologiezentrum für Hochleistungsmaterialien THM, Freiberg, Germany; 7 Saxonian Academy of Sciences, Leipzig, Germany; 8 Department of Knowledge and Communication Management, Faculty of Business and Globalization, University for Continuing Education Krems, Krems an der Donau, Austria; 9 Konrad Lorenz Institute for Evolution and Cognition Research, Klosterneuburg, Austria; 10 Arizona State University, Tempe, AZ, United States of America; 11 Santa Fe Institute, Santa Fe, NM, United States of America; Tallinn University: Tallinna Ulikool, ESTONIA

## Abstract

Interpersonal trust declined worldwide during the COVID-19 pandemic; strategies are needed to restore it. We surveyed 3,065 quota-sampled German-speaking adults residing in the D-A-CH region. Using multinomial logistic regression models and backward elimination for variable selection, we calculated multivariable-adjusted odds ratios (OR) and 95% confidence intervals (95% CIs) to appraise correlates of interpersonal trust using the Interpersonal Trust Short Scale (KUSIV3). Participants with high levels of interpersonal trust (top KUSIV3 tertile (T3)) tended to be older, male, residents of Switzerland, university degree holders, and workers with higher income and work satisfaction (all P_diff_<0.01) compared to those in the lowest KUSIV3 tertile (T1). Optimism was most strongly associated with high interpersonal trust (OR_T3vsT1_ = 5.75, 95%CI = 4.33–7.64). Also significantly associated with high interpersonal trust were: Having voted in the last national election (for the opposition, OR = 1.39, 95%CI = 1.02–1.89 or the governing party, OR = 1.61, 95%CI = 1.23–2.11) versus non-voters; perspective taking (OR_T3vsT1_ = 1.46, 95%CI = 1.11–1.91); being more extraverted (OR_T3vsT1_ = 1.99, 95%CI = 1.53–2.59) and more agreeable (OR_T3vsT1_ = 1.95, 95% CI = 1.46–2.61); and scoring higher on complexity thinking (OR_T3vsT1_ = 1.32, 95%CI = 1.01–1.72). Participants scoring significantly lower for interpersonal trust did not regularly participate in religious meetings (OR = 0.61, 95%CI = 0.44–0.84, versus participation at least monthly); were more conscientious (OR_T3vsT1_ = 0.68, 95%CI = 0.51–0.91) or current smokers (OR = 0.68; 95%CI = 0.53–0.87, versus never smoking); had sleep problems >5 times a week (OR = 0.48; 95%CI = 0.36–0.66, versus none); and scored high on conspiracy belief (OR_T3vsT1_ = 0.53; 95%CI = 0.41–0.69). Results differed minimally by gender and country. These findings may be helpful in devising targeted strategies to strengthen interpersonal trust and social engagement in European societies, especially during times of crises.

## Introduction

Trust, a broad term that essentially describes a capacity to have strong confidence in someone or something, fosters collaboration and enables constructive response to crises [1]. However, the COVID-19 pandemic crisis appears to have weakened rather than fostered trust within societies [2]. This erosion of trust may, at least partially, have been driven by social isolation during pandemic lockdowns, considering the importance of the multiple factors affecting interpersonal trust. These range from social relationships [3] at a broader level to oxytocin at a molecular level–and were likely all impacted by pandemic lockdowns [4]. With state structures, systems of governance and scientific institutions (democracy) also at stake in Western Europe, strategies to promote trust and thus engagement in society are urgently needed. The rapid increase in antidemocratic movements that rely on or appeal to conspiracy theories and to mistrust in scientific and governmental institutions observed during the COVID-19 pandemic calls for urgent action [5].

Interpersonal trust reflects the willingness of individuals to accept vulnerability due to expectations that are placed on the other person’s behavior, essentially encapsulating individuals’ predisposition to trust one another [6]. Interpersonal trust is central to human behavior and social life. It is essential for successful interpersonal relationships, and is considered a key component of social capital [7]. Studies on interpersonal trust need to be framed within a larger context that takes into account the trustworthiness of actors and the relationships between them [8]. The integrative neuropsychoeconomic (NPE) model of interpersonal trust proposed by Krueger and Meyer-Lindenberg [9] describes the evolution of trust from calculus-based, to knowledge-based, to identification-based trust, calling for the inclusion of aspects beyond the economic context and extending to other real-world scenarios (family, institutions). The NPE model also postulates that individual differences in propensity to trust may be driven by variability in functional connectivity in large-scale dynamic networks of the brain [9]. Importantly, this embedded perspective on trust calls for (lab and field) studies to improve our understanding of the interaction of culture, nurture, and nature in shaping human trust (or lack thereof) in specific social, political, and cultural contexts.

Despite extensive literature on determinants of interpersonal trust [10], to our knowledge, putative correlates of trust including a wide range of personality traits and psychological measures, have not been studied during the COVID-19 pandemic in specific regions and especially in the D-A-CH region. We therefore conducted a cross-sectional study of adults in the D-A-CH region to identify potential targets for interventions aimed at strengthening interpersonal trust in societies during times of crises. Given prior literature [11], including our own [12], we hypothesized that optimism would be a particularly strong predictor of trust. We conclude that targeting optimism as well as complexity thinking, which we found to be positively correlated with interpersonal trust, can have positive effects on building trust in our societies and can thus reinforce the role of scientific institutions in fostering democratic societies.

## Materials and methods

### Study population

Data were collected through a non-probability online survey between July 21, 2021, and August 8, 2021, among 3,067 adults residing in the D-A-CH region who were quota-sampled to match the respective population distributions for age, gender, and region of residence. Participants had to be 18 years or older, German-speaking and residing in Germany, Austria or Switzerland. The survey was designed by members of the research team, i.e. the authors of this publication, and implemented by the market research institute INTERROGARE, Bielefeld, Germany, using online panels. The questionnaire comprised 74 questions on lifestyle, health, and COVID-19 related mitigation measures and behavior, taking an average of 25 minutes to complete. Response rates were not available, neither were characteristics of non-responders. Participant informed consent was implied by completing the online survey. Data were only accessed and analyzed by members of the research team and did not include participant identifying information. The study was exempt by the Ethics Board of the Medical University of Vienna from Institutional Review Board approval according to Federal Regulations 45 CFR 46.10(b).

### Variables

The survey assessed numerous sociodemographic variables, including those listed below:

**Table:** 

Age Gender Country of residence Citizenship Migration history Mother tongue Ethnicity Educational attainment	Household income Living area Marital status Work status Political preference/ involvement Participation in religious meetings Social networks (“contact with a close person I can talk to”)

Additionally, we assessed participant’s main job task, satisfaction with work and work-life balance. For the latter, we utilized the validated “*Trierer Kurzskala zur Messung von Work-Life Balance*” (TKS-WLB) [13], which comprised 5 statements ranked from 1 (totally disagree) to 6 (totally agree) and rendered a score from 5 to 30 for which a higher score indicated better work-life balance. We also assessed several health factors, including body mass index (BMI), whether participants ever had tried to lose weight, and if so, their approach (e.g. nutrition, or other strategies), frequency of physical activity (for at least 10 minutes raising heartbeat or respiratory rate), smoking status, chronic disease and depression history, the frequency of sleep-related difficulties such as initiating sleep and/or maintaining sleep and/or waking up earlier than desired, and whether these sleep difficulties had been present for more or less than 3 months.

The survey also assessed several personality characteristics. These included optimism, for which we used the validated Life-Orientation-Test revised (LOT-R), which invites responses to six items on a five‐point Likert scale (0–4), rendering a score from 0 to 24 for which higher scores indicate higher optimism [14]. To assess interpersonal trust, we utilized the validated “*Kurzskala für interpersonales Vertrauen*” (KUSIV3). This instrument features three items that are rated on a scale of 1–5 and averaged [11]. The additional personality traits of empathy and perspective taking were assessed with the validated “*Fragebogen für Empathie und Perspektivenübernahme*.” Briefly, empathy and perspective taking are assessed separately, each with nine statements rated on a six-point Likert scale (0–5), rendering a score from 0 to 45 for each trait (with higher scores indicating stronger demonstration of the traits) [15]. Further, we assessed the “Big Five” personality traits, e.g., conscientiousness, extroversion, agreeableness, openness and neuroticism using the validated Big-Five-Inventory-SOEP (BFI-S) in which three statements per trait are rated on a seven-point Likert scale (1–7), rendering a score from 3 to 21 for each trait, with higher scores suggesting higher degrees of the corresponding personality traits [16]. Lastly, we assessed whether participants considered themselves to be a “No, but” or a “Yes, and” type in conversations. In improvisation, such as in improvisational theater, the “Yes, and” type is considered key for an ability to build spontaneously on previous ideas; it is also considered essential for effective collaboration [17]. We assessed this variable because we considered that a personality type associated with teamwork might also be related to interpersonal trust.

Additionally, several variables related to the COVID-19 pandemic were assessed, including history of COVID-19 infection (positive test), approval of the COVID-19 measures implemented by the government and vaccination against COVID-19. Finally, participants were also asked whether they agreed with the following two statements: 1. Complex real problems require collaboration between scientists and practitioners in problem solving. 2. I have heard of the Sustainable Development Goals (SDGs) and consider them to be important.

#### Conspiracy and complexity

In order to assess conspiratorial and complex thinking we devised a set of additional questions. Survey participants indicated on a Likert scale (disagree, rather disagree, rather agree, agree) their views regarding the positive effect of daily activities (such as eating less meat or walking/cycling more) on their health and the environment/climate. Further, they indicated how much they agreed or disagreed with statements that investigate conspiratorial or complex thinking (using similar questions as in the survey on conspiracy theories implemented by Roose J [18]. Conspiracy and complexity score were developed according to these questions after principal component analysis (PCA), as described in detail elsewhere (see S1 File)

Briefly, PCA was used to identify the questions to be included for the conspiracy and complexity scores. Initially, we examined the factorability of the 12 survey questions intended for the construction of conspiracy and complexity score. All 12 of those questions correlated at least 0.3 with at least one other item, suggesting reasonable factorability (see S1 File). Additionally, the Kaiser-Meyer-Olkin measure of sampling adequacy was 0.86, above the commonly recommended value of 0.6, and Bartlett’s test of sphericity was significant (χ2 = 13143.96, p < 0.05). Eigenvalues of the first two principal components were 4.199 and 2.126, respectively, and explained 35% and 18% of the variance, respectively. The third, fourth and fifth factors had eigenvalues close to 1 and explained 9%, 7%, and 6% of the variance, respectively. Solutions for three, four, five, and six factors were each examined using oblimin rotations of the factor loading matrix. The two-factor solution, which explained 53% of the variance, was preferred because of its previous theoretical support [19] and the leveling of eigenvalues on the screen plot after two factors (see S1 File). For the final stage, we conducted a PCA using varimax and oblimin rotations, with two factors explaining 53% of the variance. The oblimin rotation provided the best-defined factor structure. All factors in this analysis had primary loadings over 0.55. The factor loading matrix for this final solution is presented (see S1 File).

Internal consistency for each of the scales was examined using Cronbach’s alpha [20]. The alpha was moderate, at 0.69, for the complexity score, and high, at 0.87, for the conspiracy score. By eliminating the candidate questionnaire statement, “Pandemics like COVID-19 are connected to climate change.” from the complexity score, the Cronbach’s alpha could have increased to 0.74, but the question was purposefully retained because we found the question to be of high importance to gauge complex thinking.

Composite scores were created, based on the sum of the items that had factor loadings >0.55 on each principal component. Higher scores indicated more complex thinking or more belief in conspiracy theories. Neither score was normally distributed as shown by the Shapiro Wilk’s test (p<0.001) and examination of the histograms (see S1 File). The conspiracy score was positively skewed, while the complexity score displayed negative skewness. Descriptive statistics for the scores are presented in Table 1.

**Table 1:** Sample characteristics (N = 3,065) across tertiles of interpersonal trust.

	Interpersonal trust				
	Lowest tertile (N = 1,043)	Middle tertile (N = 1,007)	Highest tertile (N = 1,015)		
	N (%)	N (%)	N (%)		p-value _[__1__]_
**Interpersonal trust** ^[^^2^^]^					
*Median (interquartile range)*	2.33 (2.00–2.67)	3.00 (3.00–3.33)	4 .00 (3.67–4.33)		<0.001
*Range*	1.00–2.67	3.00–3.33	3.67–5.00		
**Age**					
18–25	120 (11.5)	123 (12.2)	95 (9.4)		
26–35	188 (18.0)	165 (16.3)	124 (12.2)		
36–45	186 (17.8)	205 (20.4)	178 (17.5)		
46–55	192 (18.4)	170 (16.9)	193 (19.0)		
56–65	211 (20.3)	168 (16.7)	217 (21.4)		
≥66	146 (14.0)	176 (17.5)	208 (20.5)		<0.001
**Gender**					
Women	567 (54.4)	518 (51.4)	482 (47.5)		
Men	476 (45.6)	489 (48.6)	533 (52.5)		0.007
**Country of residence**					
Austria	371 (35.6)	329 (32.7)	319 (31.4)		
Germany	362 (34.7)	338 (33.6)	323 (31.8)		
Switzerland	310 (29.7)	340 (33.7)	373 (36.8)		0.019
**Citizenship**					
Austrian	333 (31.9)	310 (30.8)	299 (29.4)		
German	384 (36.8)	352 (34.9)	342 (33.7)		
Swiss	262 (25.1)	294 (29.2)	340 (33.5)		
Other, EU	39 (3.7)	34 (3.4)	21 (2.1)		
Other, Non-EU	25 (2.4)	17 (1.7)	13 (1.3)		0.003
**Ethnicity**					
White	938 (89.9)	917 (91.1)	950 (93.5)		
Other than white	105 (10.1)	90 (8.9)	66 (6.5)		0.013
**Migration history**					
First generation	262 (25.1)	306 (30.4)	264 (26.0)		
Second generation	120 (11.5)	93 (9.2)	84 (8.3)		
More than second generation/none	661 (63.4)	608 (60.4)	667 (65.7)		0.007
**Mother tongue**					
German	936 (89.7)	928 (92.1)	949 (93.4)		
Other than German	108 (10.3)	79 (7.9)	67 (6.6)		0.007
**Rural living area**					
Rural	558 (53.5)	566 (56.2)	533 (52.4)		
Urban	485 (46.5)	441 (43.8)	483 (47.6)		0.212
**Marital status**					
Single	334 (32.0)	296 (29.4)	283 (27.9)		
Married/partnership	556 (53.3)	568 (56.4)	604 (59.5)		
Divorced	130 (12.5)	109 (10.8)	94 (9.3)		
Widowed	23 (2.2)	34 (3.4)	34 (3.3)		0.024
**Educational attainment**					
No university degree	835 (80.1)	772 (76.7)	729 (71.8)		
University degree	208 (19.9)	235 (23.3)	286 (28.2)		<0.001
**Household income**					
Approx. lowest tertile	459 (44.0)	385 (38.2)	297 (29.3)		
Approx. middle tertile	254 (24.4)	262 (26.0)	270 (26.6)		
Approx. highest tertile	330 (31.6)	360 (35.8)	448 (44.1)		<0.001
**Work status**					
Full- (part-) time employed	419 (40.2)	396 (39.3)	406 (40.0)		
Full- (part-) time self-employed	71 (6.8)	61 (6.1)	71 (7.0)		
Unemployed	60 (5.8)	62 (6.2)	36 (3.6)		
Retired	234 (22.4)	250 (24.8)	260 (25.6)		
Student/in training/civil-/military-service	50 (4.8)	61 (6.1)	63 (6.2)		
Household	56 (5.4)	48 (4.8)	46 (4.5)		
Temporary contract	25 (2.4)	13 (1.3)	19 (1.9)		
Permanent contract	128 (12.3)	116 (11.5)	114 (11.2)		0.215
**Satisfaction with work**					
No, does not or does rather not apply	377 (36.2)	260 (25.8)	173 (17.0)		
Yes, does rather apply	456 (43.7)	536 (53.2)	496 (48.9)		
Yes, does totally apply	210 (20.1)	211 (21.0)	346 (34.1)		<0.001
**Work-Life balance**_[__3__]_					
Bottom tertile	445 (42.7)	345 (34.3)	200 (19.7)		
Middle tertile	288 (27.6)	364 (36.1)	323 (31.8)		
Top tertile	310 (29.7)	298 (29.6)	492 (48.5)	<0.001	
**Main job task**					
Physical work with hands	197 (18.9)	160 (15.9)	152 (15.0)		
Mental work with figures/symbols	260 (24.9)	248 (24.6)	283 (27.9)		
Contact/Communication with other people	224 (21.5)	225 (22.3)	229 (22.6)		
Not working	362 (34.7)	374 (37.2)	351 (34.6)		0.160
**Political preference** (last elections)					
Did not vote	370 (35.5)	298 (29.6)	208 (20.5)		
Opposition parties	282 (27.0)	234 (23.2)	259 (25.5)		
Governing parties	391 (37.5)	475 (47.2)	548 (54.0)		<0.001
**Participation at religious meetings**					
At least once a month	111 (10.6)	163 (16.2)	139 (13.7)		
Less than once a month	132 (12.7)	158 (15.7)	180 (17.7)		
Never, or almost never	800 (76.7)	686 (68.1)	696 (68.6)		<0.001
**Contact with a close person (except children)**					
Less than once a week	132 (12.7)	122 (12.1)	54 (5.3)		
At least once a week	193 (18.5)	181 (18.0)	190 (18.7)		
Daily	718 (68.8)	704 (69.9)	771 (76.0)		<0.001
**In conversations I consider myself a:**					
“No, but…” type	380 (36.4)	269 (26.7)	215 (21.2)		
“Yes, and…” type	663 (63.6)	738 (73.3)	800 (78.8)		<0.001
**Optimism**_[__4__]_					
Bottom tertile	569 (54.6)	403 (40.0)	176 (17.3)		
Middle tertile	271 (26.0)	323 (32.1)	225 (22.2)		
Top tertile	203 (19.4)	281 (27.9)	614 (60.5)		<0.001
**Empathy**_[__5__]_					
Bottom tertile	406 (39.0)	332 (33.0)	225 (22.2)		
Middle tertile	259 (24.8)	325 (32.3)	341 (33.6)		
Top tertile	378 (36.2)	350 (34.7)	449 (44.2)		<0.001
**Perspective taking**_[__5__]_					
Bottom tertile	446 (42.8)	333 (33.1)	222 (21.9)		
Middle tertile	236 (22.6)	291 (28.9)	283 (27.9)		
Top tertile	361 (34.6)	383 (38.0)	510 (50.2)		<0.001
**Conscientiousness**_[__6__]_					
Bottom tertile	403 (38.6)	429 (42.6)	235 (23.2)		
Middle tertile	277 (26.5)	283 (28.1)	335 (33.0)		
Top tertile	363 (34.8)	295 (29.3)	445 (43.8)		<0.001
**Extroversion**_[__6__]_					
Bottom tertile	410 (39.3)	275 (27.3)	231 (22.7)		
Middle tertile	338 (32.4)	423 (42.0)	318 (31.3)		
Top tertile	295 (28.3)	309 (30.7)	466 (46.0)		<0.001
**Agreeableness**_[__6__]_					
Bottom tertile	491 (47.1)	402 (39.9)	220 (21.7)		
Middle tertile	304 (29.1)	351 (34.9)	370 (36.5)		
Top tertile	248 (23.8)	254 (25.2)	425 (41.8)		<0.001
**Openness**_[__6__]_					
Bottom tertile	448 (43.0)	384 (38.1)	300 (29.6)		
Middle tertile	284 (27.2)	298 (29.6)	314 (30.9)		
Top tertile	311 (29.8)	325 (32.3)	401 (39.5)		<0.001
**Neuroticism**_[__6__]_					
Bottom tertile	261 (25.0)	225 (22.3)	435 (42.8)		
Middle tertile	156 (14.9)	203 (20.2)	184 (18.1)		<0.001
Top tertile	627 (60.1)	579 (57.5)	397 (39.1)		
**COVID-19 infection (positive test)**	67 (6.4)	81 (8.0)	68 (6.7)		0.312
**Approval of the COVID-19 measures implemented by the government**					
No, they were unnecessary/unjustified	199 (19.1)	135 (13.4)	86 (8.5)		
Yes, partially	394 (37.8)	389 (38.6)	293 (28.9)		
Yes, mainly or totally	450 (43.1)	483 (48.0)	636 (62.6)		<0.001
**Vaccinated against COVID-19**					
Fully immunized (second shot or Johnson&Johnson)	653 (62.6)	668 (66.3)	765 (75.4)		
Partially immunized (first shot)	72 (6.9)	81 (8.0)	62 (6.1)		
Not yet, but made an appointment to get vaccinated	66 (6.3)	63 (6.3)	49 (4.8)		
No, won´t get vaccinated	252 (21.2)	195 (19.4)	139 (13.7)		<0.001
**BMI** [kg/m^2^] _[__7__]_					
Underweight [BMI<18·5]	36 (3.5)	34 (3.4)	26 (2.6)		
Normal weight [BMI≥18·5 & <25]	414 (39.7)	427 (42.4)	437 (43.1)		
Overweight [BMI≥25 & <30]	289 (27.7)	301 (29.9)	314 (30.9)		
Obesity [BMI≥30]	202 (19.4)	164 (16.3)	182 (17.9)		0.011
**Frequency of physical activity done for at least 10 minutes that raises the heartbeat or the respiratory rate**					
Less than once a week	276 (26.5)	177 (17.6)	169 (16.7)		
1–2 days a week	269 (25.8)	280 (27.8)	252 (24.8)		
3–4 days a week	243 (23.3)	284 (28.2)	283 (27.9)		
5–7 days a week	255 (24.4)	266 (26.4)	311 (30.6)		<0.001
**Smoking status**					
Never	385 (36.9)	443 (44.0)	454 (44.7)		
Former	269 (25.8)	282 (28.0)	288 (28.4)		
Current	389 (37.2)	282 (28.0)	273 (26.9)		<0.001
**Chronic disease**_[__8__]_					
None	622 (59.6)	610 (60.6)	590 (58.1)		
At least one	421 (40.4)	397 (39.4)	425 (41.9)		0.527
**Depression**					
Never	818 (78.4)	848 (84.2)	892 (87.9)		
Ever	225 (21.6)	159 (15.8)	123 (12.1)		<0.001
**Difficulty falling asleep in the last 4 weeks**_[__9__]_					
None	206 (19.8)	285 (28.3)	318 (31.3)		
Once a week	40 (3.8)	49 (4.9)	77 (7.6)		
1–2 times a week	246 (23.6)	232 (23.0)	233 (23.0)		
3–4 times a week	272 (26.1)	231 (22.9)	227 (22.3)		
More than 5 times a week	279 (26.8)	210 (20.9)	160 (17.8)		<0.001
**Duration of sleep problems** (regarding the abovementioned)					
**≤ 3 months**	268 (32.0)	297 (41.1)	271 (38.9)		
**> 3 months**	569 (67.9)	425 (58.9)	426 (1.0)		<0.001
**Complex real problems require the collaboration between scientists and practitioners in problem solving**					
Do not agree at all	33 (3.2)	15 (1.5)	10 (1.0)		
Rather not agree	118 (11.3)	89 (8.8)	37 (3.6)		
Rather agree	538 (51.6)	582 (57.8)	474 (46.7)		
Agree	354 (33.9)	321 (31.9)	494 (48.7)		<0.001
**I have heard of the SDGs and consider them to be important**					
Do not agree at all	282 (27.0)	199 (19.8)	225 (22.2)		
Rather not agree	347 (33.3)	309 (30.7)	238 (23.5)		
Rather agree	339 (32.5)	409 (40.6)	384 (37.8)		
Agree	75 (7.2)	90 (8.9)	168 (16.5)		<0.001
**Conspiracy score**_[__10__]_					
Bottom tertile	320 (30.7)	373 (37.0)	555 (54.7)		
Middle tertile	212 (20.3)	179 (17.8)	176 (17.3)		
Top tertile	511 (49.0)	455 (45.2)	284 (28.0)		<0.001
**Complexity score**_[__11__]_					
Bottom tertile	466 (44.7)	430 (42.7)	302 (29.8)		
Middle tertile	289 (27.7)	292 (29.0)	310 (30.5)		
Top tertile	288 (27.6)	285 (28.3)	403 (39.7)		<0.001
**Weight loss**					
Yes, I have tried losing weight and I lost the weight I wanted to lose	306 (29.3)	307 (30.5)	340 (33.4)		
Yes, I have tried losing weight but I have not lost the weight I wanted to lose	355 (34.0)	333 (33.1)	315 (31.0)		
Yes, I have tried losing weight but I have not lost any	100 (9.6)	89 (8.8)	79 (7.8)		
No, I never have tried to lose weight	282 (27.0)	278 (27.6)	281 (27.7)		0.366
**If you ever have tried to lose weight, what did you focus on?**					
Nutrition & physical activity	499 (65.6)	480 (65.8)	552 (75.2)		
Only nutrition	192 (25.2)	186 (25.5)	145 (19.7)		
Only physical activity	42 (5.5)	45 (6.2)	24 (3.3)		
Nether nutrition nor physical activity	28 (3.7)	18 (2.5)	13 (1.8)		<0.001

[1] P-values were calculated using Pearson’s chi-squared test for categorical and k-sample equality-of-medians test for continuous variables because Shapiro-Wilk tests indicated that continuous variables were not normally distributed

[2] KUSIV-3 (15)

[3] TKS-WLB (13)

[4] LOT-R (14)

[5] Questionnaire for empathy and perspective taking, German version (16)

[6] BFI-S (17)

[7] 239 participants had missing information on BMI

[8] Asthma, COPD, chronical bronchitis, emphysema, heart attack, angina pectoris or coronary heart disease, cancer, hypertension, stroke or diabetes

[9] Report of difficulty initiating sleep and/or difficulty maintaining sleep and/or waking up earlier than desired.

[10] For derivation see S1 File

[11] For derivation see S1 File

### Statistical methods

Descriptive statistics were used to summarize characteristics of the whole study population across tertiles of interpersonal trust. For categorical variables, frequencies were reported and Pearson’s chi-squared tests used to test for differences between countries. For continuous variables, median and interquartile range (*IQR*) were reported after Shapiro-Wilk tests indicated that the continuous variables were not normally distributed. We used k-sample equality-of-medians tests to compare findings across tertiles of interpersonal trust. We investigated different transformation approaches but did not find one that adequately normalized the continuous variables. Thus, we decided to categorize continuous variables into tertiles based on the total study sample.

For the univariable analyses, we used multinomial logistic regression models to calculate odds ratios (OR) and 95% confidence intervals (CI) to investigate factors associated with interpersonal trust. Ordered logistic regression was not applied because the parallel regression assumption was violated. Too few participants reported a gender other than man or woman (n = 2) to be analyzed separately, and thus those participants were excluded from all analyses, leaving a final sample of 3,065 participants.

The following factors were investigated in univariable analyses:

**Table:** 

**Age [18–25; 26–35; 36–45; 46–55; 56–65; older than 65]**	Optimism [tertiles]
Gender	Interpersonal trust [tertiles]
Country of residence [Austria; Germany; Switzerland]	Empathy [tertiles]
Citizenship [Austrian; German; Swiss; Other, EU; Other, Non-EU]	Perspective taking [tertiles]
Migration history [first generation; second generation; more than second-generation/none]	Conscientiousness [tertiles]
Ethnicity [White; non-White]	Extroversion [tertiles]
Mother tongue [German; other than German]	Agreeableness [tertiles]
Educational attainment [No university degree; university degree]	Openness [tertiles]
Household income [lowest; intermediate; highest tertile in the country of residence] Living area [urban; rural]	Neuroticism [tertiles]
Marital status [single; married/partnership; divorced; widowed]	A previous or current COVID-19 infection
Work status [Full-(part-)time employed; Full-(part-)time self-employed; unemployed; retired; student/in training/civil-/military service; household; temporary contract; permanent contract]	Weight loss [Yes, I have tried losing weight and I lost the weight I wanted to lose; Yes, I have tried losing weight but I have not lost the weight I wanted to lose; Yes, I have tried losing weight but I have not lost any; No, I never have tried to lose weight]
Satisfaction with work [No, does (rather) not apply; Yes, does rather apply; Yes, does apply]	Frequency of physical activity (for at least 10 minutes raising heartbeat or respiratory rate) [less than once a week; 1–2 days a week; 3–4 days a week; 5–7 days a week]
Work-life balance [tertiles]	Smoking status [never; former; current]
Political involvement/choice in the last elections [did not vote; voted for the opposition party, voted for the governing party]	Chronic disease history [asthma; COPD; chronical bronchitis; emphysema; heart attack; angina pectoris or coronary heart disease; cancer; hypertension; stroke or diabetes].
Type of sleep problem [sleep-related difficulties such as initiating sleep and/or maintaining sleep and/or waking up earlier than desired]	Frequency of sleep problems in the last 4 weeks [none; once a week; 1–2 times a week; 3–4 times a week; more than 5 times a week]
Contact with a close person I can talk to (except children) [less than once a week; at least once a week; daily]	Duration of sleep problems [≤3 months; >3 months]
Self-reported type in conversations [“No, but…” type; “Yes, and…” type]	Approval of the statement: “Complex real problems require collaboration between scientists and practitioners in problem solving” [Do not agree at all or rather not agree; rather agree; agree]
Approval of the COVID-19 measures implemented by the government [No, they were unnecessary and unjustified; Yes, partially; Yes, mainly or totally]	Approval of the statement: “I have heard of the SDGs and consider them to be important” [Do not agree at all; rather not agree; rather agree; agree]
BMI [kg/m2]	Derived score for conspiracy thinking [tertiles]
Participation at religious meetings [at least once a month; less than once a month; never or almost never]	Derived score for complexity thinking [tertiles]

For the multivariable regression analyses, we initially included all variables that were associated (p<0.05) with trust in univariable analyses. We then utilized backward stepwise selection to eliminate variables with a p-value ≥ 0.05 to arrive at our final models. We followed this approach for the whole sample and for each country and gender separately. Variance inflation factors (VIF) indicated no multicollinearity in the multivariable models (VIF<4). We used missing indicators to represent missing data in the models. A two-sided *p*-value less than 0.05 was considered statistically significant.

#### Transparency and promotion

We describe our sampling plan, all data exclusions, all manipulations, and all measures in the study, and we adhered to the Journal of Applied Psychology methodological checklist. All data, analysis code, and research materials are available upon reasonable request. This study’s design and its analysis were not preregistered. All data analyses were performed using STATA (version 14.1, 2015, StataCorp LP).

## Results

### Participant characteristics

Of the 3,065 survey participants included in the analysis, 48·8% were men. The age ranged between 18 and 90 years (mean = 48, *SD* = 16.5). Some socioeconomic characteristics, e.g., educational attainment, as well as several personality characteristics differed across tertiles of interpersonal trust (Table 1). For example, interpersonal trust was higher among men (52.5% in top tertile) compared to women (47.5% in top tertile) and highest among participants residing in Switzerland (participants in top tertile of interpersonal trust: Switzerland 36.8%; Germany, 31.8%; Austria, 31.4%).

### Correlates of trust

Among all 3,065 participants, the median score in the top tertile of interpersonal trust was 4.00 (Interquartile range within the tertile (IQR): 3.67–4.33), compared to 2.33 (IQR: 2.00–2.67) among those who were in the bottom tertile of interpersonal trust.

The multivariable adjusted models using backward stepwise selection identified additional variables to retain in the model as factors significantly associated with high interpersonal trust (Table 2). Of all the variables in the final model, optimism was the most strongly associated with interpersonal trust (OR_T3vsT1_ = 5.75, 95%CI = 4.33–7.64). Additionally, persons who had voted in the last national election were more likely to experience high levels of interpersonal trust regardless of which party they chose (OR_opposition party voters_ = 1.39, 95%CI = 1.02–1.89 and OR_governing party voters_ = 1.61, 95%CI = 1.23–2.11), compared to non-voters. Perspective taking (OR_T3vsT1_ = 1.46, 95%CI = 1.11–1.91) and being more extroverted (OR_T3vsT1_ = 1.99, 95%CI = 1.53–2.59) and more agreeable (OR_T3vsT1_ = 1.95, 95%CI = 1.46–2.61) was also associated with higher level of interpersonal trust. Similarly, individuals who scored higher on complexity thinking exhibited higher level of interpersonal trust (OR_T3vsT1_ = 1.32, 95%CI = 1.01–1.72). In contrast, variables that were significantly associated with low levels of interpersonal trust included not regularly participating in religious meetings (OR = 0·61, 95%CI = 0·44–0·84, versus participation at least monthly) and being more conscientious (OR_T3vsT1_ = 0.68, 95%CI = 0.51–0.91). Further, being a current smoker (OR = 0.68; 95%CI = 0.53–0.87, versus never smoking) or having sleep problems more than 5 times a week (OR = 0.48; 95%CI = 0.36–0.66, versus none) were both associated with significantly lower level of interpersonal trust. Lastly, scoring high on COVID-19 conspiracies was a significant predictor of low interpersonal trust (OR_T3vsT1_ = 0.53; 95%CI = 0.41–0.69).

**Table 2:** Factors cross-sectionally associated with interpersonal trust in the D-A-CH region (N = 3,065).

	Interpersonal trust										
	Lowest tertile (N = 1,043)	Middle tertile (N = 1,007)					Highest tertile (N = 1,015)				
	N (%)	N (%)	OR_crude_ (95% CI)	p	OR_adj._ (95% CI) ^[^^1^^]^	p ^[^^1^^]^	N (%)	OR_crude_ (95% CI)	p-value	OR_adj._ (95% CI) ^[^^1^^]^	p-value ^[^^1^^]^
**Age**											
18–25	120 (11.5)	123 (12.2)	Ref.				95 (9.4)	Ref.			
26–35	188 (18.0)	165 (16.4)	0.86 (0.62–1.19)	0.352			124 (12.2)	0.83 (0.59–1.18)	0.309		
36–45	186 (17.9)	205 (20.3)	1.08 (0.78–1.48)	0.657			178 (17.5)	1.21 (0.86–1.70)	0.272		
46–55	192 (18.4)	170 (16.9)	0.86 (0.62–1.20)	0.378			193 (19.0)	1.27 (0.91–1.78)	0.163		
56–65	211 (20.2)	168 (16.7)	0.78 (0.56–1.07)	0.125			217 (21.4)	1.30 (0.93–1.81)	0.119		
≥66	146 (14.0)	176 (17.5)	1.18 (0.84–1.64)	0.341			208 (20.5)	1.80 (1.28–2.53)	0.001		
**Men**	567 (54.4)	518 (51.4)	Ref.					Ref.			
**Women**	476 (45.6)	489 (48.6)	1.12 (0.95–1.34)	0.185			482 (47.5)	1.32 (1.11–1.57)	0.002		
**Country of residence**							533 (52.5)				
Austria	371 (35.6)	329 (32.7)	Ref.		Ref.			Ref.		Ref.	
Germany	362 (34.7)	338 (33.6)	1.05 (0.85–1.30)	0.630	1.04 (0.83–1.31)	0.740	319 (31.4)	1.04 (0.84–1.28)	0.732	1.24 (0.96–1.59)	0.100
Switzerland	310 (29.7)	340 (33.7)	1.24 (1.00–1.53)	0.051	1.22 (0.96–1.56)	0.104	323 (31.8)	1.40 (1.13–1.73)	0.002	1.62 (1.25–2.12)	<0.001
**Citizenship**_[__2__]_							373 (36.8)				
Austrian	333 (31.9)	310 (30.8)	Ref.					Ref.			
German	384 (36.8)	352 (34.9)	0.98 (0.80–1.22)	0.886			299 (29.4)	0.99 (0.80–1.23)	0.941		
Swiss	262 (25.1)	294 (29.2)	1.21 (0.96–1.51)	0.107			342 (33.7)	1.45 (1.15–1.81)	0.001		
Other, EU	39 (3.7)	34 (3.4)	0.94 (0.58–1.52)	0.791			340 (33.5)	0.60 (0.34–1.04)	0.070		
Other, Non-EU	25 (2.4)	17 (1.7)	0.73 (0.39–1.38)	0.333			21 (2.1)	0.58 (0.29–1.15)	0.120		
**Ethnicity**							13 (1.3)				
White	938 (89.9)	917 (91.1)	Ref.					Ref.			
Other than white	105 (10.1)	90 (8.9)	0.88 (0.65–1.18)	0.384			950 (93.5)	0.62 (0.45–0.86)	0.004		
**Migration history**							66 (6.5)				
First generation	262 (25.1)	306 (30.4)	Ref.		Ref.			Ref.		Ref.	
Second generation	120 (11.5)	93 (9.2)	0.66 (0.48–0.91)	0.011	0.63 (0.45–0.89)	0.008	264 (26.0)	0.69 (0.50–0.96)	0.029	0.71 (0.48–1.03)	0.074
More than second generation/none	661 (63.4)	608 (60.4)	0.79 (0.65–0.96)	0.018	0.77 (0.62–0.96)	0.023	84 (8.3)	1.00 (0.82–1.23)	0.989	0.92 (0.72–1.18)	0.497
**Mother tongue**							667 (65.7)				
German	936 (89.7)	928 (92.1)	Ref.		Ref.			Ref.		Ref.	
Other than German	108 (10.3)	79 (7.9)	0.74 (0.54–1.00)	0.049	0.62 (0.43–0.87)	0.007	949 (93.4)	0.61 (0.45–0.84)	0.002	0.70 (0.47–1.05)	0.083
**Living area**							67 (6.6)				
Urban	558 (53.5)	566 (56.2)	Ref.					Ref.			
Rural	485 (46.5)	441 (43.8)	0.90 (0.75–1.07)	0.218			533 (52.4)	1.05 (0.88–1.24)	0.622		
**Marital status**							483 (47.6)				
Single	334 (32.0)	296 (29.4)	Ref.					Ref.			
Married/partnership	556 (53.3)	568 (56.4)	1.15 (0.95–1.40)	0.154			283 (27.9)	1.28 (1.05–1.56)	0.013		
Divorced	130 (12.5)	109 (10.8)	0.95 (0.70–1.28)	0.716			604 (59.5)	0.85 (0.63–1.16)	0.315		
Widowed	23 (2.2)	34 (3.4)	1.67 (0.96–2.90)	0.069			94 (9.3)	1.74 (1.00–3.03)	0.048		
**Educational attainment**							34 (3.3)				
No university degree	835 (80.1)	772 (76.7)	Ref.					Ref.			
University degree	208 (19.9)	235 (23.3)	1.22 (0.99–1.51)	0.062			729 (71.8)	1.57 (1.28–1.93)	<0.001		
**Household income**							286 (28.2)				
Bottom tertile	459 (44.0)	385 (38.2)	Ref.					Ref.			
Middle tertile	254 (24.4)	262 (26.0)	1.23 (0.99–1.53)	0.065			297 (29.3)	1.64 (1.31–2.06)	<0.001		
Highest tertile	330 (31.6)	360 (35.8)	1.30 (1.06–1.59)	0.011			270 (26.6)	2.10 (1.71–2.57)	<0.001		
**Work status**							448 (44.1)				
Full- (part-) time employed	419 (40.2)	396 (39.3)	Ref.		Ref.			Ref.		Ref.	
Full- (part-) time self-employed	71 (6.8)	61 (6.1)	0.91 (0.63–1.31)	0.612	0.92(0.62–1.36)	0.657	406 (40.0)	1.03 (0.72–1.47)	0.862	0.95 (0.63–1.43)	0.791
Unemployed	60 (5.8)	62 (6.2)	1.09 (0.75–1.60)	0.646	1.72 (1.14–2.62)	0.011	71 (7.0)	0.62 (0.40–0.96)	0.031	1.46 (0.88–2.45)	0.147
Retired	234 (22.4)	250 (24.8)	1.13 (0.90–1.42)	0.286	1.29 (1.00–1.66)	0.052	36 (3.6)	1.15 (0.92–1.43)	0.229	0.93 (0.71–1.23)	0.611
Student/in training/civil-/military-service	50 (4.8)	61 (6.1)	1.29 (0.87–1.92)	0.209	1.38 (0.89–2.15)	0.146	260 (25.6)	1.30 (0.88–1.93)	0.193	1.67 (1.04–2.68	0.032
Household	56 (5.4)	48 (4.8)	0.91 (0.60–1.37)	0.640	1.12 (0.72–1.74)	0.605	63 (6.2)	0.85 (0.56–1.28)	0.433	1.05 (0.64–1.70)	0.856
Temporary contract	25 (2.4)	13 (1.3)	0.55 (0.28–1.09)	0.087	0.57 (0.28–1.19)	0.134	46 (4.5)	0.78 (0.43–1.45)	0.437	0.88 (0.42–1.86)	0.740
Permanent contract	128 (12.3)	116 (11.5)	0.96 (0.72–1.28)	0.774	0.91 (0.67–1.23)	0.541	19 (1.9)	0.92 (0.69–1.22)	0.565	1.04 (0.75–1.46)	0.798
**Satisfaction with work**											
No, does not or does rather not apply	377 (36.2)	260 (25.8)	Ref.		Ref.		114 (11.2)	Ref.		Ref.	
Yes, does rather apply	456 (43.7)	536 (53.2)	1.70 (1.40–2.08)	<0.001	1.38 (1.10–1.74)	0.006	173 (17.0)	2.37 (1.90–2.95)	<0.001	1.39 (1.07–1.82)	0.015
Yes, does totally apply	210 (20.1)	211 (21.0)	1.46 (1.14–1.87)	0.003	1.18 (0.87–1.58)	0.290	496 (48.9)	3.59 (2.80–4.60)	<0.001	1.23 (0.89–1.70)	0.207
**Work-Life balance** ^[^^3^^]^											
Bottom tertile	445 (42.7)	345 (34.3)	Ref.		Ref.		200 (19.7)	Ref.		Ref.	
Middle tertile	288 (27.6)	364 (36.1)	1.63 (1.32–2.01)	<0.001	1.20 (0.95–1.52)	0.129	323 (31.8)	2.50 (1.98–3.14)	<0.001	1.35 (1.03–1.78)	0.030
Top tertile	310 (29.7)	298 (29.6)	1.24 (1.00–1.53)	<0.047	0.95 (0.72–1.25)	0.710	492 (48.5)	3.53 (2.84–4.40)	<0.001	1.12 (0.84–1.51)	0.438
**Political preference** (last elections)											
Did not vote	370 (35.5)	298 (29.6)	Ref.		Ref.		370 (35.5)	Ref.		Ref.	
Opposition parties	282 (27.0)	234 (23.2)	1.03 (0.82–1.30)	0.800	0.98 (0.75–1.29)	0.909	282 (27.0)	1.63 (1.29–2.08)	<0.001	1.39 (1.02–1.88)	0.035
Governing parties	391 (37.5)	475 (47.2)	1.51 (1.23–1.85)	<0.001	1.25 (0.99–1.58)	0.065	391 (37.5)	2.49 (2.01–3.09)	<0.001	1.61 (1.23–2.11)	<0.001
**Participation at religious meetings**											
At least once a month	111 (10.6)	163 (16.2)	Ref.		Ref.		111 (10.6)	Ref.		Ref.	
Less than once a month	132 (12.7)	158 (15.7)	0.82 (0.58–1.13)	0.230	0.77 (0.54–1.10)	0.155	132 (12.7)	1.09 (0.78–1.52)	0.619	0.79 (0.54–1.18)	0.251
Never, or almost never	800 (76.7)	686 (68.1)	0.58 (0.45–0.76)	<0.001	0.66 (0.49–0.89)	0.006	800 (76.7)	0.69 (0.53–0.91)	0.008	0.61 (0.44–0.84)	0.003
**Contact with a close person (except children)**											
Less than once a week	132 (12.7)	122 (12.1)	Ref.				132 (12.7)	Ref.			
At least once a week	193 (18.5)	181 (18.0)	1.01 (0.74–1.40)	0.929			193 (18.5)	2.41 (1.65–3.50)	<0.001		
Daily	718 (68.8)	704 (69.9)	1.06 (0.81–1.39)	0.665			718 (68.8)	2.62 (1.88–3.66)	<0.001		
**In conversations I consider myself a:**											
“No, but…” type	380 (36.4)	269 (26.7)	Ref.		Ref.		380 (36.4)	Ref.		Ref.	
“Yes, and…” type	663 (63.6)	738 (73.3)	1.57 (1.30–1.90)	<0.001	1.24 (1.01–1.52)	0.039	663 (63.6)	2.13 (1.75–2.60)	<0.001	1.25 (0.99–1.58)	0.058
**Optimism**_[__4__]_											
Bottom tertile	569 (54.6)	403 (40.0)	Ref.		Ref.		569 (54.6)	Ref.		Ref.	
Middle tertile	271 (26.0)	323 (32.1)	1.68 (1.37–2.07)	<0.001	1.54 (1.22–1.93)	<0.001	271 (26.0)	2.68 (2.10–3.43)	<0.001	2.01 (1.53–2.64)	<0.001
Top tertile	203 (19.4)	281 (27.9)	1.95 (1.57–2.44)	<0.001	1.95 (1.49–2.55)	<0.001	203 (19.4)	9.78 (7.75–12.3)	<0.001	5.75 (4.33–7.64)	<0.001
**Empathy**_[__5__]_											
Bottom tertile	406 (39.0)	332 (33.0)	Ref.				406 (39.0)	Ref.			
Middle tertile	259 (24.8)	325 (32.3)	1.53 (1.23–1.91)	<0.001			259 (24.8)	2.38 (1.89–2.99)	<0.001		
Top tertile	378 (36.2)	350 (34.7)	1.13 (0.92–1.39)	0.236			378 (36.2)	2.14 (1.73–2.65)	<0.001		
**Perspective taking**_[__5__]_											
Bottom tertile	446 (42.8)	333 (33.1)	Ref.		Ref.		446 (42.8)	Ref.		Ref.	
Middle tertile	236 (22.6)	291 (28.9)	1.65 (1.32–2.06)	<0.001	1.41 (1.10–1.80)	0.007	236 (22.6)	2.41 (1.90–3.05)	<0.001	1.60 (1.21–2.12)	0.001
Top tertile	361 (34.6)	383 (38.0)	1.42 (1.16–1.74)	0.001	1.24 (0.97–1.58)	0.091	361 (34.6)	2.84 (2.30–3.50)	<0.001	1.46 (1.11–1.91)	0.006
**Conscientiousness**_[__6__]_											
Bottom tertile	403 (38.6)	429 (42.6)	Ref.		Ref.		403 (38.6)	Ref.		Ref.	
Middle tertile	277 (26.5)	283 (28.1)	0.96 (0.77–1.19)	0.707	0.75 (0.59–0.96)	0.022	277 (26.5)	2.07 (1.65–2.60)	<0.001	1.05 (0.80–1.38)	0.740
Top tertile	363 (34.8)	295 (29.3)	0.76 (0.62–0.94)	0.010	0.55 (0.42–0.71)	<0.001	363 (34.8)	2.10 (1.70–2.60)	<0.001	0.68 (0.51–0.91)	0.009
**Extroversion**_[__6__]_											
Bottom tertile	410 (39.3)	275 (27.3)	Ref.			Ref.	410 (39.3)	Ref.		Ref.	
Middle tertile	338 (32.4)	423 (42.0)	1.87 (1.51–2.30)	<0.001	1.67 (1.33–2.09)	<0.001	338 (32.4)	1.67 (1.34–2.09)	<0.001	1.71 (1.32–2.22)	<0.001
Top tertile	295 (28.3)	309 (30.7)	1.56 (1.25–1.95)	<0.001	1.48 (1.16–1.90)	0.002	295 (28.3)	2.80 (2.26–3.48)	<0.001	1.99 (1.53–2.59)	<0.001
**Agreeableness**_[__6__]_											
Bottom tertile	491 (47.1)	402 (39.9)	Ref.		Ref.		491 (47.1)	Ref.		Ref.	
Middle tertile	304 (29.1)	351 (34.9)	1.41 (1.15–1.73)	0.001	1.32 (1.05–1.66)	0.019	304 (29.1)	2.72 (2.18–3.38)	<0.001	1.74 (1.34–2.25)	<0.001
Top tertile	248 (23.8)	254 (25.2)	1.25 (1.00–1.56)	0.045	1.32 (1.00–1.73)	0.052	248 (23.8)	3.82 (3.06–4.78)	<0.001	1.95 (1.46–2.61)	<0.001
**Openness**_[__6__]_											
Bottom tertile	448 (43.0)	384 (38.1)	Ref.				448 (43.0)	Ref.			
Middle tertile	284 (27.2)	298 (29.6)	1.22 (0.99–1.51)	0.062			284 (27.2)	1.65 (1.33–2.05)	<0.001		
Top tertile	311 (29.8)	325 (32.3)	1.22 (0.99–1.50)	0.060			311 (29.8)	1.93 (1.56–2.37)	<0.001		
**Neuroticism**_[__6__]_											
Bottom tertile	261 (25.0)	225 (22.3)	Ref.		Ref.		261 (25.0)	Ref.		Ref.	
Middle tertile	156 (14.9)	203 (20.2)	1.51 (1.15–1.99)	0.003	1.58 (1.17–1.51)	0.003	156 (14.9)	0.71 (0.54–0.92)	0.010	1.14 (0.83–1.56)	0.264
Top tertile	627 (60.1)	579 (57.5)	1.07 (0.87–1.33)	0.514	1.36 (1.05–1.75)	0.020	627 (60.1)	0.38 (0.31–0.46)	<0.001	0.89 (0.68–1.16)	0.007
**COVID-19 infection (positive test)**	67 (6.4)	67 (6.4)	1.27 (0.91–1.78)	0.157			67 (6.4)	1.05 (0.74–1.48)	0.801		
**Approval of the COVID-19 measures implemented by the government**											
No, they were unnecessary/unjustified	199 (19.1)	199 (19.1)	Ref.			Ref.	199 (19.1)	Ref.		Ref.	
Yes, partially	394 (37.8)	394 (37.8)	1.46 (1.12–1.89)	0.005	1.14 (0.85–1.51)	0.390	394 (37.8)	1.72 (1.28–2.31)	<0.001	1.22 (0.86–1.73)	0.264
Yes, mainly or totally	450 (43.1)	450 (43.1)	1.58 (1.23–2.04)	<0.001	1.11 (0.83–1.50)	0.485	450 (43.1)	3.27 (2.47–4.32)	<0.001	1.61 (1.14–2.30)	0.007
**Vaccinated against COVID-19**											
Fully immunized (second shot or Johnson&Johnson)	653 (62.6)	653 (62.6)	Ref.				653 (62.6)	Ref.			
Partially immunized (first shot)	72 (6.9)	72 (6.9)	1.10 (0.79–1.54)	0.578			72 (6.9)	0.74 (0.52–1.05)	0.089		
Not yet, but made an appointment to get vaccinated	66 (6.3)	66 (6.3)	0.93 (0.65–1.34)	0.708			66 (6.3)	0.63 (0.43–0.93)	0.020		
No, won´t get vaccinated	252 (21.2)	252 (21.2)	0.76 (0.61–0.94)	0.011			252 (21.2)	0.47 (0.37–0.59)	<0.001		
**BMI** [kg/m^2^] _[__7__]_											
Normal weight [BMI≥18·5 & <25]	414 (39.7)	427 (42.4)	Ref.				414 (39.7)	Ref.			
Underweight [BMI<18·5]	36 (3.5)	34 (3.4)	0.92 (0.56–1.49)	0.723			36 (3.5)	0.68 (0.41–1.15)	0.154		
Overweight [BMI≥25 & <30]	289 (27.7)	301 (29.9)	1.01 (0.82–1.25)	0.928			289 (27.7)	1.03 (0.84–1.27)	0.786		
Obesity [BMI≥30]	202 (19.4)	164 (16.3)	0.79 (0.62–1.01)	0.057			202 (19.4)	0.85 (0.67–1.09)	0.198		
**Frequency of physical activity done for at least 10 minutes that raises the heartbeat or the respiratory rate**											
Less than once a week	276 (26.5)	177 (17.6)	Ref.		Ref.		276 (26.5)	Ref.		Ref.	
1–2 days a week	269 (25.8)	280 (27.8)	1.62 (1.26–2.09)	<0.001	1.34 (1.02–1.77)	0.037	269 (25.8)	1.53 (1.18–1.98)	<0.001	1.21 (0.89–1.64)	0.235
3–4 days a week	243 (23.3)	284 (28.2)	1.82 (1.41–2.35)	<0.001	1.43 (1.08–1.91)	0.014	243 (23.3)	1.90 (1.47–2.46)	<0.001	1.12 (0.82–1.54)	0.467
5–7 days a week	255 (24.4)	266 (26.4)	1.63 (1.26–2.10)	<0.001	1.25 (0.94–1.66)	0.124	255 (24.4)	1.99 (1.55–2.57)	<0.001	0.94 (0.69–1.28)	0.685
**Smoking status**											
Never	385 (36.9)	443 (44.0)	Ref.		Ref.		385 (36.9)	Ref.		Ref.	
Former	269 (25.8)	282 (28.0)	0.91 (0.73–1.13)	0.398	0.92 (0.72–1.15)	0.442	269 (25.8)	0.91 (0.73–1.13)	0.378	0.91 (0.71–1.18)	0.476
Current	389 (37.2)	282 (28.0)	0.63 (0.51–0.77)	<0.001	0.69 (0.55–0.86)	0.001	389 (37.2)	0.60 (0.48–0.73)	<0.001	0.68 (0.53–0.87)	0.002
**Chronic disease**_[__8__]_	421 (40.4)	397 (39.4)	0.96 (0.81–1.15)	0.664			425 (41.9)	1.06 (0.89–1.27)	0.487		
**Depression** (ever)	225 (21.6)	159 (15.8)	0.68 (0.54–0.85)	0.001			123 (12.1)	0.50 (0.39–0.64)	<0.001		
**Sleep problems in the last 4 weeks**_[__9__]_											
None	206 (19.8)	285 (28.3)	Ref.		Ref.		318 (31.3)	Ref.		Ref.	
Once a week	40 (3.8)	49 (4.9)	0.89 (0.56–1.39)	0.600	0.79 (0.49–1.28)	0.346	77 (7.6)	1.25 (0.82–1.90)	0.303	0.96 (0.60–1.55)	0.883
1–2 times a week	246 (23.6)	232 (23.0)	0.68 (0.53–0.88)	0.003	0.63 (0.48–0.82)	0.001	233 (23.0)	0.61 (0.48–0.79)	<0.001	0.66 (0.49–0.88)	0.005
3–4 times a week	272 (26.1)	231 (22.9)	0.61 (0.48–0.79)	<0.001	0.62 (0.47–0.81)	<0.001	227 (22.3)	0.54 (0.42–0.79)	<0.001	0.67 (0.50–0.89)	0.007
More than 5 times a week	279 (26.8)	210 (20.9)	0.54 (0.42–0.70)	<0.001	0.61 (0.47–0.81)	0.001	160 (17.8)	0.37 (0.29–0.48)	<0.001	0.48 (0.36–0.66)	<0.001
**Duration of sleep problems** (regarding the abovementioned) > 3 months _[__9__]_	569 (67.9)	425 (58.9)	0.67 (0.55–0.83)	<0.001			426 (1.0)	0.74 (0.60–0.91)	0.005		
**Complex real problems require the collaboration between scientists and practitioners in problem solving**											
Do not agree at all or rather not agree	151 (14.5)	104 (10.3)	Ref.		Ref.		47 (4.6)	Ref.		Ref.	
Rather agree	538 (51.6)	582 (57.8)	1.57 (1.19–2.07)	0.001	1.38 (1.01–1.88)	0.043	474 (46.7)	2.83 (1.99–4.02)	<0.001	1.52 (1.02–2.28)	0.042
Agree	354 (33.9)	321 (31.9)	1.32 (0.98–1.76)	0.065	1.18 (0.84–1.67)	0.346	494 (48.7)	4.48 (3.15–6.39)	<0.001	1.62 (1.06–2.47)	0.027
**I have heard of the SDGs and consider them to be important**											
Do not agree at all	282 (27.0)	199 (19.8)	Ref.		Ref.		225 (22.2)	Ref.		Ref.	
Rather not agree	347 (33.3)	309 (30.7)	1.26 (1.00–1.60)	0.055	1.10 (0.85–1.43)	0.459	238 (23.5)	0.86 (0.68–1.09)	0.218	0.91 (0.69–1.21)	0.538
Rather agree	339 (32.5)	409 (40.6)	1.71 (1.36–2.16)	<0.001	1.28 (0.99–1.65)	0.059	384 (37.8)	1.42 (1.13–1.78)	0.003	1.07 (0.81–1.41)	0.626
Agree	75 (7.2)	90 (8.9)	1.70 (1.19–2.42)	0.003	1.43 (0.97–2.12)	0.073	168 (16.5)	2.81 (2.03–3.88)	<0.001	1.84 (1.24–2.71)	0.002
**Conspiracy score**_[__10__]_											
Bottom tertile	320 (30.7)	373 (37.0)	Ref.		Ref.		555 (54.7)	Ref.		Ref.	
Middle tertile	212 (20.3)	179 (17.8)	0.72 (0.56–0.93)	0.011	0.69 (0.53–0.91)	0.008	176 (17.3)	0.48 (0.38–0.61)	<0.001	0.56 (0.42–0.74)	<0.001
Top tertile	511 (49.0)	455 (45.2)	0.76 (0.63–0.93)	0.007	0.74 (0.58–0.94)	0.013	284 (28.0)	0.32 (0.26–0.39)	<0.001	0.53 (0.41–0.69)	<0.001
**Complexity score**_[__11__]_											
Bottom tertile	466 (44.7)	430 (42.7)	Ref.		Ref.		302 (29.8)	Ref.		Ref.	
Middle tertile	289 (27.7)	292 (29.0)	1.09 (0.89–1.35)	0.395	0.93 (0.73–1.16)	0.504	310 (30.5)	1.66 (1.33–2.05)	<0.001	1.18 (0.91–1.52)	0.213
Top tertile	288 (27.6)	285 (28.3)	1.07 (0.87–1.32)	0.513	0.90 (0.71–1.15)	0.415	403 (39.7)	2.16 (1.75–2.66)	<0.001	1.32 (1.01–1.72)	0.042
**Weight loss**											
Yes, I have tried losing weight and I lost the weight I wanted to lose	306 (29.3)	307 (30.5)	Ref.				340 (33.4)	Ref.			
Yes, I have tried losing weight but I have not lost the weight I wanted to lose	355 (34.0)	333 (33.1)	0.93 (0.75–1.16)	0.545			315 (31.0)	0.80 (0.64–0.99)	0.042		
Yes, I have tried losing weight but I have not lost any	100 (9.6)	89 (8.8)	0.89 (0.64–1.23)	0.472			79 (7.8)	0.71 (0.51–0.99)	0.045		
No, I never have tried to lose weight	282 (27.0)	278 (27.6)	0.98 (0.78–1.24)	0.881			281 (27.7)	0.90 (0.72–1.12)	0.345		

[1] mutually adjusted for all variables for which adjusted odds ratios with 95% confidence intervals and adjusted p-values are reported.

[2] citizenship was excluded from multivariable models due to multicollinearity

[3] TKS-WLB (13)

[4] LOT-R (14)

[5] Questionnaire for empathy and perspective taking, German version (16)

[6] BFI-S (17)

[7] 239 missing values. Missing indicators were used in multivariable models.

[8] Asthma, COPD, chronical bronchitis, emphysema, heart attack, angina pectoris or coronary heart disease, cancer, hypertension, stroke or diabetes

[9] Report of difficulty initiating sleep and/or difficulty maintaining sleep and/or waking up earlier than desired.

[10] For derivation see S1 File

[11] For derivation see S1 File

Although results differed slightly by gender (see S1 and S2 Tables) and country (see S3–S5 Tables), patterns of associations remained largely unchanged in these substrata.

## Discussion

In the present sample of adult residents of the D-A-CH region, we found that optimism, complexity thinking, as well as several sociodemographic and lifestyle factors strongly correlated with level of interpersonal trust. Those associations demonstrated independence in multivariable models (e.g., with mutual adjustment of the variables for one another). Further, although we observed some variation in levels of interpersonal trust reported by gender and country of residence, the association of other variables with level of interpersonal trust remained largely consistent across substrata defined by those factors. With the exception of complexity thinking, for which, to our knowledge, no prior evidence existed, earlier literature on predictors of interpersonal trust among different populations is mostly in line with our findings.

Age-related changes in trust behavior have previously been documented by cross-sectional studies [21], and findings largely agree with our results suggesting that interpersonal trust increases with older age. This is further substantiated by neuroscience, which has linked certain age-related changes in brain circuitry to changes in trust behavior [22]. Similarly, gender differences in interpersonal trust behavior have long been noted, suggesting that men have higher trust than women due to their lower sensitivity to risk and betrayal. A recent study using functional magnetic resonance imaging (fMRI) to distinguish neural signatures of trust by gender corroborates these findings [23]. In our data, we also observed slightly higher levels of interpersonal trust among the participants from Switzerland, versus Germany and Austria. Other studies have described variation in interpersonal trust across regions [24], but reasons are complex and they need to be put into the context of the respective political and societal context of a given region that could modify known associates of interpersonal trust (e.g., income). For example, one might speculate that the higher levels of trust observed in Switzerland could be related to the higher average income in Switzerland; nonetheless, our results were robust to adjustment for a variety of socio-demographic variables across the three D-A-CH regions. Higher education [25] and income [26] are two other well established predictors of higher interpersonal trust; reassuringly, our findings are very consistent with those earlier reports. Also in line with our findings, trust and work satisfaction have previously been shown to positively correlate; however, the direction of the relationship is less clear given that higher trust may induce higher engagement at work [27] and not the other way around. Longitudinal data would be needed to clarify the direction of this association; we are unable to contribute this insight with our cross-sectional survey.

Apart from the noted demographic factors, other traits and attributes emerged in our study as associated with interpersonal trust. Optimism exhibited the strongest association with trust, suggesting that persons with higher levels of optimism are more than 5 times as likely to also exhibit higher interpersonal trust, compared to those with comparatively low levels of optimism. The temporal directionality of this association cannot be addressed due to the cross-sectional nature of our data; but previous studies have suggested a strong correlation between these two factors [11, 28, 29]. Jones [30] argued that trust constitutes an attitude of optimism, suggesting that optimism antecedes trust. Additional factors that were positively associated with trust in our data can be viewed as facets of trust (e.g., the trait of agreeableness) and cheerfulness (e.g., the trait of extroversion).

Few previous studies examined the association between voting behavior and trust, and the scarce literature that exists suggests that generalized interpersonal trust is associated with a higher willingness to engage in political and social activities [5, 31]. Alternatively, a study by Albright et al. [32] implicated smoking behavior to be associated with voting behavior, and thus, indirectly with trust. Interestingly, we too, found a significant association between both current smoking and non-voting with lower levels of trust. A possible causal explanation relates to the increasing stigmatization of smokers [33], which, through the close alignment of social and political engagement, could lead to lower voting behavior among smokers. That we also see lower levels of trust among those not participating in religious meetings could be another indication of such social disengagement. However, of note, in our analyses, each of these factors (smoking, non-voting, no participation in religious meetings) remained significant after mutual adjustment. Further, we found that scoring higher for belief in COVID-19 conspiracies was associated with lower interpersonal trust,—a finding that has consistently been shown during the COVID-19 pandemic [34, 35].

An interesting and potentially important finding of our study is that increasing ability for complex thinking was associated with higher levels of trust. Complexity thinking allows one to see deep similarities between the structures and dynamics of different phenomena. While trust itself represents a highly complex construct, the ability to engage in complex thinking was positively associated with trust in our analyses (and in contrast, being more conscientious, a facet of achievement striving [36], was inversely associated with trust). There is little to no literature on the relationship between complexity thinking, optimism, and interpersonal trust; however, in an increasingly complex and crisis-ridden world, it seems of utmost importance to pay careful attention to these aspects beginning even in the earliest years in school. Our data may help to encourage the development of effective strategies for nurturing these edifying traits from an early age.

Lastly, sleep problems were significantly linked with lower interpersonal trust in our study. Krueger and Meyer-Lindenberg [9] postulated trust as a central component to mental health disorders such as schizophrenia or borderline disorder; and sleeping difficulties frequently co-occur with health disorders, particularly mental health problems [37]. In contrast, optimists have been shown to sleep better [12]. In a South Korean/Taiwanese study of social determinants of self-reported sleep problems, the authors found interpersonal trust not to be significantly associated with sleep problems in their multivariable models [38]. This contrasts somewhat with our findings, though cultural differences cannot be excluded. It would seem interesting to examine effect modification of the association between sleeping problems and trust by mental health disorders, such as depression, but we did not collect information on mental health in our survey.

Our study has several strengths, including the large number of socio-demographic variables and personality traits, as well as newly developed complexity and COVID-19 conspiracy belief scores, that were assessed; the population representativeness of our large sample, which allowed for good generalizability of our results and comparisons among three different German-speaking countries; and the use of multiple validated tools to assess a variety of personality traits and psychological constructs. Some limitations also deserve noting. The cross-sectional nature of our survey precludes assessing the temporality of several associations (e.g., whether work satisfaction increases trust or vice-versa). Even though we used validated instruments where possible, the purely self-reported nature of some items (e.g., sleeping difficulties) may have introduced some misclassification bias. However, our main outcome measure to assess interpersonal trust was the validated “*Kurzskala für interpersonales Vertrauen*” (KUSIV3). This instrument has subsequently been shown to correlate well with other trust scales such as e.g., the Interpersonal Trust Scenario Questionnaire (ITSQ) (29). As noted earlier, we did not query information on mental health status, which prohibited us examining different strata (with or without mental health conditions). That our survey was implemented in German precluded participation of non-German speaking participants, especially in Switzerland but also in Germany and Austria and limited the generalizability of our findings to these population groups.

In summary, our study suggests several factors that could serve as potential targets for interventions aimed at strengthening interpersonal trust and thereby people’s engagement in society. We found several previously established associates of interpersonal trust, such as age, gender, education and work-related variables; but also several new and potentially actionable associations, most importantly optimism (which can be increased through simple interventions [39]) and complexity thinking. The latter may provide an intervention point to implement complexity thinking training in schools and universities, as the world is facing numerous crises with increasing needs for complex thinking and higher levels of engagement in society, which in turn relies on higher interpersonal trust.

## Supporting information

S1 File Derivation of complexity and conspiracy score. (DOCX)

S1 Table Factors cross-sectionally associated with interpersonal trust among men (N = 1,498). (DOCX)

S2 Table Factors cross-sectionally associated with interpersonal trust among women (N = 1,567). (DOCX)

S3 Table Factors cross-sectionally associated with interpersonal trust in Austria (N = 1,019). (DOCX)

S4 Table Factors cross-sectionally associated with interpersonal trust in Germany (N = 1,023). (DOCX)

S5 Table Factors cross-sectionally associated with interpersonal trust in Switzerland (N = 1,023). (DOCX)
