# Supplementary material for: Determinants of trust in times of crises: A cross-sectional study of 3,065 German-speaking adults from the D-A-CH region
Source: PLoS One. 2023 Oct 12;18(10):e0286488. doi: 10.1371/journal.pone.0286488 (PMC10569553; doi:10.1371/journal.pone.0286488)
Supplement: S1 File — (DOCX) [file pone.0286488.s001.docx]

**Supporting Information:**

**Derivation of complexity and conspiracy score:**

We used principal component analysis (PCA) to identify the questions that we ultimately included to derive scores for belief in pandemic-related conspiracies (hereafter referred to as a ‘conspiracy belief score’) and for degree of complexity thinking. Initially, we examined the factorability of the 12 survey questions intended for the construction of the two scores (Table S1). All 12 of the questions had correlations of at least 0.3 with at least one other item, suggesting reasonable factorability (Figure S1). Additionally, the Kaiser-Meyer-Olkin measure of sampling adequacy (1) was 0.86, which is greater than the commonly recommended value of 0.6, and Bartlett’s test of sphericity (2) was significant (χ2 (66) = 13143.96, p < 0.05). We therefore conducted the PCA using all 12 survey items. Eigenvalues of the first two principal components were 4.199 and 2.126, respectively, and explained 35% and 18% of the variance, respectively (Table S1). The third, fourth and fifth factors had eigenvalues of approximately 1.0 and explained 9%, 7%, and 6% of the variance, respectively. Solutions for three, four, five, and six factors were each examined using oblimin rotations of the factor loading matrix. The two-factor solution, which explained nearly 53% of the variance, was preferred because of its previous theoretical support (13) and the leveling of eigenvalues on the scree plot after two factors. (Figures S2, S3) For the final stage, we compared two-factor solutions from PCAs that used varimax and oblimin rotations and found that an oblimin rotation provided the best-defined factor structure, and thus we utilized that solution to derive the final two scores for analysis (Table S3), as elaborated below. We note that the estimates of communality, which capture the percentage of variation of each variable, indicated that the higher the communality was of a PC, the better it is described by the variable (Table S3). Communality refers to a measure of the percentage of variation of a variable (in this case either conspiracy belief or complexity thinking) explained by each of the corresponding survey questions.

We examined the internal consistency for each of the scales using Cronbach’s alpha. This alpha was moderate for the complexity score at 0.69 and high for the conspiracy score at 0.87. By eliminating the item “Pandemics like COVID-19 are connected to climate change.” from the complexity score, the Cronbach’s alpha would have increased to 0.74. Nonetheless, we retained that item because we considered the statement an example of complex thinking.

We created composite scores for belief in conspiracy theories and complexity thinking, based on the sum of the respective items that had a factor loading of >0.55 for the corresponding principal component (Table S3). Each score had possible values from 5 to 20; higher scores indicated more belief in conspiracy theories or more complex thinking.

To guide the analyses to identify factors associated with belief in pandemic-related conspiracies, we assessed multicollinearity with the variation inflation factor (VIF) and identified two variables, “empathy” and “main job task,” with high collinearity with one or more other variables based on a VIF >5. Due to the non-normal distributions of derived conspiracy belief and complexity thinking scores (Shapiro Wilk’s p<0.001, Figure S4), we classified participants into approximate tertile (T) for each score and qualitatively interpreted the tertiles as ‘low’ (T1), ‘medium’ (T2) or ‘high’ (T3) for the respective score.

**Tables and Figures, Legends:**

Table S1. Factor loadings, communalities, eigenvalues and proportion of variance explained for the first two principal components from a principal components analysis with oblimin rotation for 12 items from the D-A-CH survey (N = 3067).

Table S2. Comparison of AICs from subset models considered during the process of best subset selection.

Table S3. Confusion matrix comparing observed and predicted category of score for belief in pandemic-related conspiracies among 3,065 participants in the survey of D-A-CH region residents.

Table S4. Distributions of responses to questions that contributed to the complexity and conspiracy scores, overall and stratified by country of residence.

Figure S1. Pairwise correlations of variables evaluated for use in the derived complexity and conspiracy scores.

Figure S2. Principal component analysis, loading plot of variables evaluated for use in the derived complexity and conspiracy scores

Figure S3. Scree plot.

Figure S4. Frequency of survey participants based on their conspiracy and complexity scores.

**Table S1. Factor loadings, communalities, eigenvalues and proportion of variance explained for the first two principal components from a principal components analysis with oblimin rotation for 12 items from the D-A-CH survey (N = 3067).^†^**

|  | PC1**^‡^** | PC2* | Communality^§^ |
| --- | --- | --- | --- |
| Coronaviruses are only invented and do not exist. | **0.821** | 0.150 | 0.70 |
| A virus will be developed to infect and kill already vaccinated persons. | **0.816** | 0.202 | 0.71 |
| The coronavirus is just used as a pretext to oppress people. | **0.810** | 0.094 | 0.67 |
| Microchips are implanted with the vaccination. | **0.797** | 0.204 | 0.68 |
| The vaccine against the corona virus is transmissible from person to person. | **0.639** | 0.233 | 0.46 |
| The climate is changing more and more through the influence of humans. | -0.417 | **0.670** | 0.62 |
| If I eat less meat, it has a positive effect on my health and the environment/climate. | -0.272 | **0.649** | 0.49 |
| Climate change exists. | -0.503 | **0.609** | 0.62 |
| If I walk and cycle more, it has a positive effect on my health and the environment/climate. | -0.382 | **0.581** | 0.48 |
| Pandemics like COVID-19 are connected to climate change. | 0.261 | **0.560** | 0.38 |
| The coronavirus came from a Chinese laboratory. | 0.550 | 0.155 | 0.33 |
| Coronaviruses are similar to common flu viruses. | 0.376 | 0.205 | 0.18 |
|  |  |  |  |
| Eigenvalue | 4.199 | 2.126 | - |
| Proportion of variance explained^¥^ | 0.350 | 0.177 | - |

Abbreviations: D-A-CH: Germany-Austria-Switzerland; PC: principal component.

^†^ Participants indicated their degree of agreement with each of the 12 items on a 5-level Likert scale.

^‡^ The survey items with factor loadings in bold type in this column had factor loadings of >0.55 for the first principal component and suggested belief in pandemic-related conspiracies. Those items were included in the derivation of the composite score for belief in conspiracies.

*The survey items with factor loadings in bold type in this column had factor loadings of >0.55 for the second principal component and suggested stronger complexity thinking. Those items were included in the derivation of the composite score for complexity thinking.

^§^ Communality refers to a measure of the percentage of variation of a variable (in this case either conspiracy belief or complexity thinking) explained by each of the corresponding survey questions.

^¥^ Collectively, the first two principal components explain 52.7% of the variance in these data.

**Table S2. Comparison of AICs from subset models considered during the process of best subset selection.**

| Model | Variables | AIC |
| --- | --- | --- |
| Full model with 32 variables | Age, gender, ethnicity, migration history, country of residence, residential area, highest education, income tertile, living alone, marital status, number of children under 16 years, voting behavior at last election, frequency of attending religious meetings, work status, change in work status, number of people one talks to daily, number of close contacts, smoking status, chronic disease, tested positive for COVID-19, approval of COVID-19 measures, trust, optimism, perspective taking, work life balance, conscientiousness, extraversion, agreeableness, openness, neuroticism, cross-classification of participant and close contacts vaccination status, complexity thinking score | 3770.453 |
| Reduced model | Full model minus the following variables: marital status, work status, change in work status | 3773.210 |
| Subset model 1 | Full model minus the following variables: country of residence, residential area, living alone, marital status, work status, change in work status, number of people one talks to daily, chronic disease | 3758.896 |
| Subset model 2 = Best subset model = Final model | Full model minus the following variables: migration history, country of residence, residential area, living alone, marital status, work status, change in work status, number of people one talks to daily, chronic disease, neuroticism | **3755.613** |

Abbreviations: AIC: Akaike information criterion.

**Table S3. Confusion matrix comparing observed and predicted category of score for belief in pandemic-related conspiracies among 3,065 participants in the survey of D-A-CH region residents. ^†^**

|  | Predicted low conspiracy belief | Predicted medium conspiracy belief | Predicted high conspiracy belief | n of total participant per tertile of conspiracy belief |
| --- | --- | --- | --- | --- |
| Tertile of low conspiracy belief score | 953 | 200 | 95 | 1248 |
| Tertile of medium conspiracy belief score | 428 | 355 | 198 | 981 |
| Tertile of high conspiracy belief score | 125 | 174 | 537 | 836 |
| n of total predicted groups | 1506 | 729 | 830 | Total: 3065 |

**^†^**Misclassification of model by the final combined dataset: 39.80% (Misclassification by training dataset was 38.70% and by replication dataset 37.00%, respectively). Misclassification by tertile category of belief in pandemic-related conspiracies: low: 23.64%; medium: 63.81%; high: 35.77%.

**Table S4. Distributions of responses to survey questions that contributed to the derived complexity and conspiracy scores, overall and stratified by country of residence.**

|  |  | **Country of residence** | | |
| --- | --- | --- | --- | --- |
|  | **Overall (N=3067)** | **Germany (N=1025)** | **Austria (N=1019)** | **Switzerland (N=1023)** |
| **Questions included for the derived complexity thinking score** | | | | |
| **If I eat less meat, it has a positive effect on my health and the environment/climate.** | | | | |
| I disagree | 307 (10.0%) | 100 (9.8%) | 87 (8.5%) | 120 (11.7%) |
| I rather disagree | 665 (21.7%) | 206 (20.1%) | 215 (21.1%) | 244 (23.9%) |
| I rather agree | 1357 (44.2%) | 477 (46.5%) | 446 (43.8%) | 434 (42.4%) |
| I agree | 738 (24.1%) | 242 (23.6%) | 271 (26.6%) | 225 (22.0%) |
| **If I walk and cycle more, it has a positive effect on my health and the environment/climate.** | | | | |
| I disagree | 112 (3.7%) | 49 (4.8%) | 35 (3.4%) | 28 (2.7%) |
| I rather disagree | 198 (6.5%) | 84 (8.2%) | 52 (5.1%) | 62 (6.1%) |
| I rather agree | 1225 (39.9%) | 405 (39.5%) | 383 (37.6%) | 437 (42.7%) |
| I agree | 1532 (50.0%) | 487 (47.5%) | 549 (53.9%) | 496 (48.5%) |
| **Climate change exists.** | | | | |
| I disagree | 125 (4.1%) | 46 (4.5%) | 37 (3.6%) | 42 (4.1%) |
| I rather disagree | 233 (7.6%) | 82 (8.0%) | 68 (6.7%) | 83 (8.1%) |
| I rather agree | 884 (28.8%) | 302 (29.5%) | 269 (26.4%) | 313 (30.6%) |
| I agree | 1825 (59.5%) | 595 (58.0%) | 645 (63.3%) | 585 (57.2%) |
| **Pandemics like COVID-19 are connected to climate change.** | | | | |
| I disagree | 1194 (38.9%) | 373 (36.4%) | 404 (39.6%) | 417 (40.8%) |
| I rather disagree | 1046 (34.1%) | 326 (31.8%) | 352 (34.5%) | 368 (36.0%) |
| I rather agree | 633 (20.6%) | 235 (22.9%) | 213 (20.9 %) | 185 (18.1%) |
| I agree | 194 (6.3%) | 91 (8.9%) | 50 (4.9.%) | 53 (5.2%) |
| **The climate is changing more and more through the influence of humans.** | | | | |
| I disagree | 146 (4.8%) | 61 (6.0%) | 40 (3.9%) | 45 (4.4%) |
| I rather disagree | 296 (9.7%) | 98 (9.6%) | 98 (9.6%) | 100 (9.8%) |
| I rather agree | 1086 (35.4%) | 374 (36.5%) | 332 (32.6%) | 380 (37.1%) |
| I agree | 1539 (50.2%) | 492 (48.0%) | 549 (53.9%) | 498 (48.7%) |
| **Questions included for the derived conspiracy belief score** | | | | |
| **Microchips are implanted with the vaccination.** | | | | |
| I disagree | 2259 (73.7%) | 723 (70.5%) | 791 (77.6%) | 745 (72.8%) |
| I rather disagree | 438 (14.3%) | 139 (13.6%) | 131 (12.9%) | 168 (16.4%) |
| I rather agree | 262 (8.5%) | 116 (11.3%) | 69 (6.8%) | 77 (7.5%) |
| I agree | 108 (3.5%) | 47 (4.6%) | 28 (2.7%) | 33 (3.2%) |
| **Corona viruses are only invented and do not exist.** | | | | |
| I disagree | 2165 (70.6%) | 715 (69.8%) | 763 (74.9%) | 687 (67.2%) |
| I rather disagree | 513 (16.7%) | 147 (14.3%) | 158 (15.5%) | 208 (20.3%) |
| I rather agree | 264 (8.6%) | 103 (10.0%) | 70 (6.9%) | 91 (8.9%) |
| I agree | 125 (4.1%) | 60 (5.9%) | 28 (2.7%) | 37 (3.6%) |
| **A virus will be developed to infect and kill already vaccinated persons.** | | | | |
| I disagree | 1906 (62.1%) | 631 (61.6%) | 668 (65.6%) | 607 (59.3%) |
| I rather disagree | 633 (20.6%) | 192 (18.7%) | 197 (19.3%) | 244 (23.9%) |
| I rather agree | 363 (11.8%) | 134 (13.1%) | 102 (10.0%) | 127 (12.4%) |
| I agree | 165 (5.4%) | 68 (6.6%) | 52 (5.1%) | 45 (4.4%) |
| **The corona virus is just used as a pretext to oppress people.** | | | | |
| I disagree | 1664 (54.3%) | 587 (57.3%) | 555 (54.5%) | 522 (51.0%) |
| I rather disagree | 599 (19.5%) | 185 (18.0%) | 201 (19.7%) | 213 (20.8%) |
| I rather agree | 478 (15.6%) | 140 (13.7%) | 157 (15.4%) | 181 (17.7%) |
| I agree | 326 (10.6%) | 113 (11.0%) | 106 (10.4%) | 107 (10.5%) |
| **The vaccine against the corona virus is transmissible from person to person.** | | | | |
| I disagree | 1966 (64.1%) | 617 (60.2%) | 708 (69.5%) | 641 (62.7%) |
| I rather disagree | 558 (18.2%) | 166 (16.2%) | 180 (17.7%) | 212 (20.7%) |
| I rather agree | 341 (11.1%) | 140 (13.7%) | 89 (8.7%) | 112 (10.9%) |
| I agree | 202 (6.6%) | 102 (10.0%) | 42 (4.1%) | 58 (5.7%) |

**Figure S1. Pairwise correlations of variables evaluated for use in the derived complexity and conspiracy scores.**


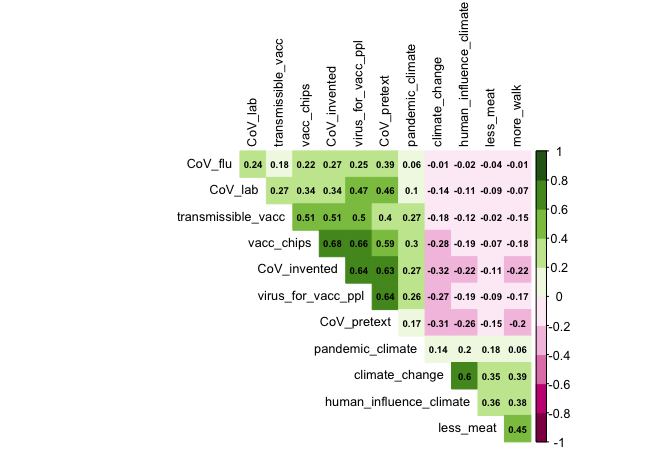


CoV_flu: Coronaviruses are similar to common flu viruses.; CoV_lab: The coronavirus came from a Chinese laboratory.; transmissible_vacc: The vaccine against the corona virus is transmissible from person to person.; vacc_chips: Microchips are implanted with the vaccination.; CoV_invented: Coronaviruses are only invented and do not exist.; virus_for_vacc_ppl: A virus will be developed to infect and kill already vaccinated persons.; CoV_pretext: The coronavirus is just used as a pretext to oppress people.; pandemic_climate: Pandemics like COVID-19 are connected to climate change.; climate_change: Climate change exists.; human_influence_climate: The climate is changing more and more through the influence of humans.; less_meat: If I eat less meat, it has a positive effect on my health and the environment/climate.; walk_more: If I walk and cycle more, it has a positive effect on my health and the environment/climate.

**Figure S2. Principal component analysis, loading plot of variables evaluated for use in the derived complexity and conspiracy scores**


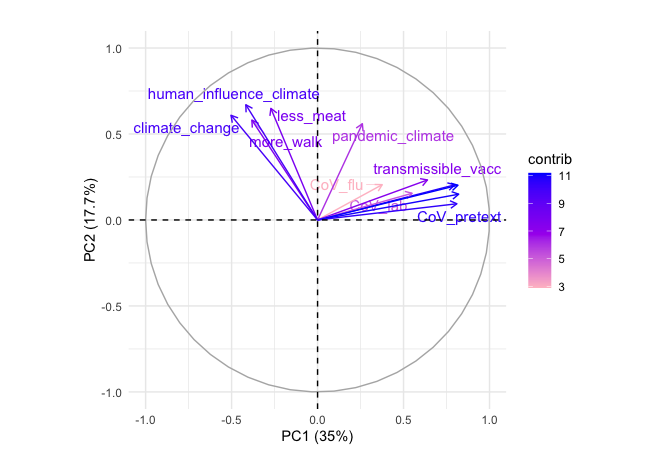


The factor loadings plot shows the variables as arrows of different length and orientation. The darker blue color indicates a higher contribution to the PC. The arrows of longer length and closer proximity suggest a higher correlation between the variables. PC1 accounts for 35.0%, while PC2 accounts for 17.7% of the total variance, and are distinct, as they have different orientations.

PC: principal component; contrib: contribution; CoV_flu: Coronaviruses are similar to common flu viruses.; CoV_lab: The coronavirus came from a Chinese laboratory.; transmissible_vacc: The vaccine against the corona virus is transmissible from person to person.; vacc_chips: Microchips are implanted with the vaccination.; CoV_invented: Coronaviruses are only invented and do not exist.; virus_for_vacc_ppl: A virus will be developed to infect and kill already vaccinated persons.; CoV_pretext: The coronavirus is just used as a pretext to oppress people.; pandemic_climate: Pandemics like COVID-19 are connected to climate change.; climate_change: Climate change exists.; human_influence_climate: The climate is changing more and more through the influence of humans.; less_meat: If I eat less meat, it has a positive effect on my health and the environment/climate.; walk_more: If I walk and cycle more, it has a positive effect on my health and the environment/climate.

**Figure S3. Scree plot.**


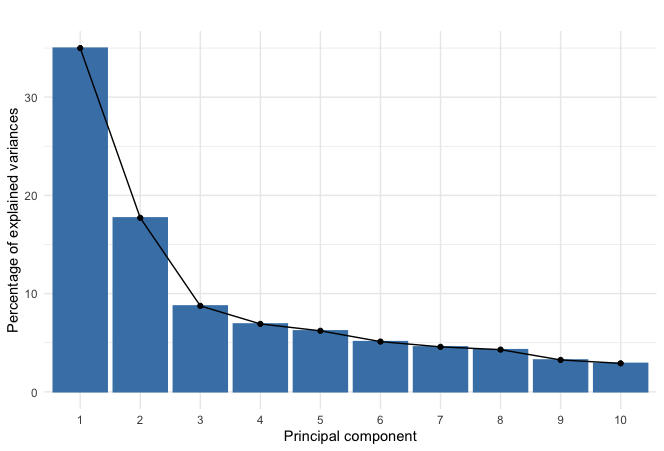


The scree plot shows the proportion of explained variation by each principal component. Accumulatively, the first two principal components explain 52.7% of the variance of the of variables evaluated for use in the derived complexity and conspiracy scores.

**Figure S4. Distribution of survey participant scores for belief in pandemic-related conspiracies and complexity thinking.**


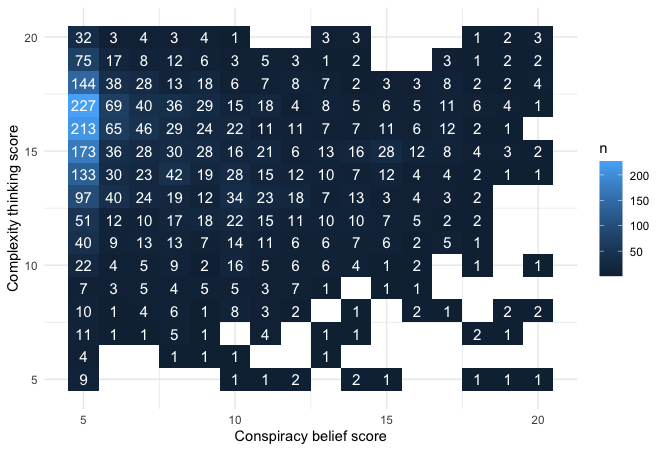


Number in cells equal counts of participants with the corresponding scores

References:

1. H. F. Kaiser, A second generation Little Jiffy. *Psychometrika* **35**, 401-415 (1970).

2. M. S. Bartlett, Properties of sufficiency and statistical tests. *Proceedings of the Royal Statistical Society* **Series A 160**, 268-282 (1937).
