# Supplementary material for: Determinants of trust in times of crises: A cross-sectional study of 3,065 German-speaking adults from the D-A-CH region
Source: PLoS One. 2023 Oct 12;18(10):e0286488. doi: 10.1371/journal.pone.0286488 (PMC10569553; doi:10.1371/journal.pone.0286488)
Supplement: S1 Table — (DOCX) [file pone.0286488.s002.docx]

| **S1 Table. Factors cross-sectionally associated with interpersonal trust among men (N=1,498).** | | | | | | | | | | | |
| --- | --- | --- | --- | --- | --- | --- | --- | --- | --- | --- | --- |
|  | Interpersonal trust | | | | | | | | | | |
|  | Lowest tertile (N=476) | Middle tertile (N=489) | | | | | Highest tertile  (N=533) | | | | |
|  | N (%) | N (%) | OR_crude_ (95% CI) | p | OR_adj._ (95% CI) ^[1]^ | p ^[1]^ | N (%) | OR_crude_ (95% CI) | p-value | OR_adj._ (95% CI) ^[1]^ | p-value ^[1]^ |
| **Age** |  |  |  |  |  |  |  |  |  |  |  |
| 18-25 | 29 (6.1) | 38 (7.8) | Ref. |  |  |  | 33 (6.2) | Ref. |  |  |  |
| 26-35 | 60 (12.6) | 63 (12.9) | 0.80 (0.44-1.46) | 0.468 |  |  | 48 (9.0) | 0.70 (0.38-1.32) | 0.271 |  |  |
| 36-45 | 90 (18.9) | 99 (20.2) | 0.84 (0.48-1.47) | 0.541 |  |  | 72 (13.5) | 0.70 (0.39-1.26) | 0.240 |  |  |
| 46-55 | 92 (19.3) | 82 (16.8) | 0.68 (0.39-1.20) | 0.183 |  |  | 106 (19.9) | 1.01 (0.57-1.79) | 0.966 |  |  |
| 56-65 | 109 (22.9) | 99 (20.2) | 0.69 (0.40-1.21) | 0.195 |  |  | 134 (25.1) | 1.08 (0.62-1.89) | 0.787 |  |  |
| ≥66 | 96 (20.2) | 108 (22.1) | 0.86 (0.49-1.50) | 0.591 |  |  | 140 (26.3) | 1.28 (0.73-2.25) | 0.387 |  |  |
| **Country of residence** |  |  |  |  |  |  |  |  |  |  |  |
| Austria | 174 (36.5) | 162 (33.2) | Ref. |  | Ref. |  | 162 (30.4) | Ref. |  | Ref. |  |
| Germany | 157 (33.0) | 163 (33.3) | 1.12 (0.82-1.51) | 0.486 | 1.08 (0.77-1.53) | 0.644 | 172 (32.3) | 1.18 (0.87-1.60) | 0.295 | 1.31 (0.91-1.90) | 0.147 |
| Switzerland | 145 (30.5) | 164 (33.5) | 1.21 (0.89-1.66) | 0.218 | 1.29 (0.91-1.84) | 0.157 | 199 (37.3) | 1.47 (1.09-2.00) | 0.012 | 1.61 (1.10-2.34) | 0.013 |
| **Citizenship** _[2]_ |  |  |  |  |  |  |  |  |  |  |  |
| Austrian | 161 (33.8) | 155 (31.7) | Ref. |  |  |  | 155 (29.1) | Ref. |  |  |  |
| German | 172 (36.1) | 169 (34.6) | 1.02 (0.75-1.39) | 0.896 |  |  | 181 (33.9) | 1.09 (0.81-1.48) | 0.566 |  |  |
| Swiss | 125 (26.3) | 143 (29.2) | 1.19 (0.86-1.65) | 0.300 |  |  | 182 (34.2) | 1.51 (1.10-2.08) | 0.011 |  |  |
| Other, EU | 9 (1.9) | 20 (4.1) | 2.31 (1.02-5.23) | 0.045 |  |  | 8 (1.5) | 0.92 (0.35-2.45) | 0.873 |  |  |
| Other, Non-EU | 9 (1.9) | 2 (0.4) | 0.23 (0.05-1.09) | 0.063 |  |  | 7 (1.3) | 0.81 (0.29-2.22) | 0.679 |  |  |
| **Ethnicity** |  |  |  |  |  |  |  |  |  |  |  |
| White | 444 (93.3) | 454 (92.8) | Ref. |  |  |  | 502 (94.2) | Ref. |  |  |  |
| Other than white | 32 (6.7) | 35 (7.2) | 1.07 (0.65-1.76) | 0.791 |  |  | 31 (5.8) | 0.86 (0.51-1.43) | 0.553 |  |  |
| **Migration history** |  |  |  |  |  |  |  |  |  |  |  |
| First generation | 113 (23.7) | 148 (30.3) | Ref. |  | Ref. |  | 140 (26.3) | Ref. |  | Ref. |  |
| Second generation | 51 (10.7) | 41 (8.4) | 0.61 (0.38-0.99) | 0.046 | 0.52 (0.31-0.87) | 0.014 | 44 (8.2) | 0.70 (0.43-1.12) | 0.134 | 0.57 (0.32-1.00) | 0.050 |
| More than second generation/none | 312 (65.6) | 300 (61.3) | 0.73 (0.55-0.98) | 0.038 | 0.78 (0.56-1.08) | 0.134 | 349 (65.5) | 0.90 (0.67-1.21) | 0.491 | 0.81 (0.57-1.15) | 0.240 |
| **Mother tongue** |  |  |  |  |  |  |  |  |  |  |  |
| German | 446 (93.7) | 460 (94.1) | Ref. |  |  |  | 504 (94.6) | Ref. |  |  |  |
| Other than German | 30 (6.3) | 29 (5.9) | 0.94 (0.55-1.59) | 0.809 |  |  | 29 (5.4) | 0.86 (0.51-1.45) | 0.561 |  |  |
| **Living area** |  |  |  |  |  |  |  |  |  |  |  |
| Urban | 263 (55.3) | 300 (61.4) | Ref. |  |  |  | 304 (57.0) | Ref. |  |  |  |
| Rural | 213 (44.7) | 189 (39.6) | 1.14 (0.97-1.35) | 0.055 |  |  | 229 (43.0) | 1.16 (0.98-1.36) | 0.085 |  |  |
| **Marital status** |  |  |  |  |  |  |  |  |  |  |  |
| Single | 138 (29.0) | 136 (27.8) | Ref. |  |  |  | 136 (25.5) | Ref. |  |  |  |
| Married/partnership | 278 (58.4) | 292 (59.7) | 1.07 (0.80-1.42) | 0.665 |  |  | 336 (63.1) | 1.23 (0.92-1.63) | 0.161 |  |  |
| Divorced | 52 (10.9) | 51 (10.4) | 1.00 (0.63-1.57) | 0.983 |  |  | 45 (8.4) | 0.88 (0.55-1.40) | 0.583 |  |  |
| Widowed | 8 (1.7) | 10 (2.0) | 1.27 (0.49-3.31) | 0.627 |  |  | 16 (3.0) | 2.03 (0.84-4.90) | 0.115 |  |  |
| **Educational attainment** |  |  |  |  |  |  |  |  |  |  |  |
| No university degree | 371 (77.9) | 361 (73.8) | Ref. |  |  |  | 382 (71.7) | Ref. |  |  |  |
| University degree | 105 (22.1) | 128 (26.2) | 1.25 (0.93-1.68) | 0.136 |  |  | 151 (28.3) | 1.40 (1.05-1.86) | 0.023 |  |  |
| **Household income** |  |  |  |  |  |  |  |  |  |  |  |
| Bottom tertile | 159 (33.4) | 155 (31.7) | Ref. |  |  |  | 125 (23.5) | Ref. |  |  |  |
| Middle tertile | 132 (27.7) | 127 (26.0) | 0.99 (0.71-1.37) | 0.938 |  |  | 136 (25.5) | 1.31 (0.94-1.83) | 0.114 |  |  |
| Highest tertile | 185 (38.9) | 207 (42.3) | 1.15 (0.85-1.55) | 0.363 |  |  | 272 (51.0) | 1.87 (1.39-2.52) | <0.001 |  |  |
| **Work status** |  |  |  |  |  |  |  |  |  |  |  |
| Full- (part-) time employed | 196 (41.2) | 194 (39.7) | Ref. |  |  |  | 210 (39.4) | Ref. |  |  |  |
| Full- (part-) time self-employed | 37 (7.8) | 33 (6.8) | 0.90 (0.54-1.50) | 0.689 | 0.94 (0.55-1.63) | 0.833 | 42 (7.9) | 1.06 (0.65-1.72) | 0.815 | 1.08 (0.62-1.89) | 0.789 |
| Unemployed | 24 (5.0) | 31 (6.3) | 1.30 (0.74-2.30) | 0.359 | 2.65 (1.36-5.15) | 0.004 | 13 (2.4) | 0.51 (0.25-1.02) | 0.057 | 1.73 (0.74-4.07) | 0.209 |
| Retired | 141 (29.6) | 140 (28.6) | 1.00 (0.74-1.36) | 0.984 | 1.28 (0.90-1.82) | 0.168 | 171 (32.1) | 1.13 (0.84-1.52) | 0.412 | 1.06 (0.74-1.53) | 0.749 |
| Student/in training/civil-/military-service | 6 (1.3) | 15 (3.1) | 2.52 (0.96-6.65) | 0.060 | 3.20 (1.13-9.09) | 0.029 | 19 (3.6) | 2.96 (1.16-7.55) | 0.024 | 4.52 (1.54-13.2) | 0.006 |
| Household | 8 (1.7) | 7 (1.4) | 0.88 (0.31-2.49) | 0.815 | 1.24 (0.41-3.80) | 0.704 | 4 (0.8) | 0.47 (0.14-1.57) | 0.219 | 0.55 (0.13-2.32) | 0.419 |
| Temporary contract | 12 (2.5) | 7 (1.4) | 0.59 (0.23-1.53) | 0.277 | 0.47 (0.17-1.34) | 0.157 | 10 (1.9) | 0.78 (0.33-1.84) | 0.567 | 0.55 (0.19-1.55) | 0.258 |
| Permanent contract | 52 (10.9) | 62 (12.7) | 1.20 (0.79-1.83) | 0.383 | 1.10 (0.70-1.73) | 0.674 | 64 (12.0) | 1.15 (0.76-1.74) | 0.512 | 1.33 (0.82-2.15) | 0.242 |
| **Satisfaction with work** |  |  |  |  |  |  |  |  |  |  |  |
| No, does not or does rather not apply | 174 (36.5) | 111 (22.7) | Ref. |  | Ref. |  | 78 (14.6) | Ref. |  | Ref. |  |
| Yes, does rather apply | 196 (41.2) | 267 (54.6) | 2.14 (1.58-2.89) | <0.001 | 1.87 (1.32-2.64) | <0.001 | 254 (47.7) | 2.89 (2.09-4.00) | <0.001 | 1.78 (1.19-2.65) | 0.005 |
| Yes, does totally apply | 106 (22.3) | 111 (22.7) | 1.64 (1.15-2.35) | 0.007 | 1.45 (0.93-2.25) | 0.099 | 201 (37.7) | 4.23 (2.96-6.04) | <0.001 | 1.41 (0.88-2.26) | 0.150 |
| **Work-Life balance** ^[3]^ |  |  |  |  |  |  |  |  |  |  |  |
| Bottom tertile | 200 (42.0) | 162 (33.1) | Ref. |  | Ref. |  | 91 (17.1) | Ref. |  | Ref. |  |
| Middle tertile | 127 (26.7) | 188 (38.5) | 1.83 (1.35-2.48) | <0.001 | 1.34 (0.95-2.08) | 0.099 | 158 (29.6) | 2.73 (1.95-3.84) | <0.001 | 1.51 (1.01-2.26) | 0.046 |
| Top tertile | 149 (31.3) | 139 (28.4) | 1.15 (0.84-1.57) | 0.372 | 0.95 (0.63-1.42) | 0.801 | 284 (53.3) | 4.19 (3.05-5.75) | <0.001 | 1.43 (0.93-2.19) | 0.106 |
| **Political preference** (last elections) |  |  |  |  |  |  |  |  |  |  |  |
| Did not vote | 119 (25.0) | 90 (18.4) | Ref. |  | Ref. |  | 75 (14.1) | Ref. |  | Ref. |  |
| Opposition parties | 162 (34.0) | 155 (31.7) | 1.27 (0.89-1.80) | 0.190 | 1.38 (0.91-2.08) | 0.132 | 163 (30.6) | 1.60 (1.11-2.29) | 0.011 | 1.26 (0.80-2.00) | 0.319 |
| Governing parties | 195 (41.0) | 244 (49.9) | 1.65 (1.19-2.31) | 0.003 | 1.56 (1.06-2.29) | 0.023 | 295 (55.3) | 2.40 (1.71-3.38) | <0.001 | 1.55 (1.02-2.36) | 0.040 |
| **Participation at religious meetings** |  |  |  |  |  |  |  |  |  |  |  |
| At least once a month | 59 (12.4) | 85 (17.4) | Ref. |  | Ref. |  | ,,,, | Ref. |  | Ref. |  |
| Less than once a month | 61 (12.8) | 81 (16.6) | 0.92 (0.58-1.47) | 0.734 | 0.82 (0.49-1.38) | 0.465 | 81 (15.2) | 0.89 (0.56-1.42) | 0.627 | 0.59 (0.34-1.02) | 0.059 |
| Never, or almost never | 356 (74.8) | 323 (66.0) | 0.63 (0.44-0.91) | 0.013 | 0.80 (0.53-1.22) | 0.301 | 364 (68.3) | 0.69 (0.48-0.98) | 0.040 | 0.62 (0.39-0.96) | 0.034 |
| **Contact with a close person (except children)** |  |  |  |  |  |  |  |  |  |  |  |
| Less than once a week | 77 (16.2) | 68 (13.9) | Ref. |  |  |  | 38 (7.1) | Ref. |  |  |  |
| At least once a week | 83 (17.4) | 90 (18.4) | 1.23 (0.79-1.91) | 0.363 |  |  | 93 (17.5) | 2.27 (1.39-3.70) | 0.001 |  |  |
| Daily | 316 (66.4) | 331 (67.7) | 1.19 (0.83-1.70) | 0.354 |  |  | 402 (75.4) | 2.58 (1.70-3.91) | <0.001 |  |  |
| **In conversations I consider myself a:** |  |  |  |  |  |  |  |  |  |  |  |
| *“No, but…” type* | 177 (37.2) | 122 (24.9) | Ref. |  | Ref. |  | 107 (20.1) | Ref. |  | Ref. |  |
| *“Yes, and…” type* | 299 (62.8) | 367 (75.1) | 1.78 (1.35-2.35) | <0.001 | 1.35 (1.00-1.84) | 0.053 | 426 (79.9) | 2.36 (1.78-3.12) | <0.001 | 1.48 (1.05-2.07) | 0.023 |
| **Optimism** _[4]_ |  |  |  |  |  |  |  |  |  |  |  |
| Bottom tertile | 240 (50.4) | 199 (40.7) | Ref. |  | Ref. |  | 93 (17.5) | Ref. |  | Ref. |  |
| Middle tertile | 137 (28.8) | 162 (33.1) | 1.43 (1.06-1.92) | 0.018 | 1.32 (0.94-1.85) | 0.108 | 125 (23.5) | 2.35 (1.67-3.31) | <0.001 | 1.60 (1.08-2.80) | 0.019 |
| Top tertile | 99 (20.8) | 128 (26.2) | 1.56 (1.13-2.15) | 0.007 | 1.47 (0.98-2.20) | 0.063 | 315 (59.0) | 8.21 (5.91-11.4) | <0.001 | 4.23 (2.78-6.43) | <0.001 |
| **Empathy** _[5]_ |  |  |  |  |  |  |  |  |  |  |  |
| Bottom tertile | 235 (49.4) | 194 (39.6) | Ref. |  |  |  | 146 (27.4) | Ref. |  |  |  |
| Middle tertile | 112 (23.5) | 167 (34.2) | 1.81 (1.33-2.45) | <0.001 |  |  | 189 (35.5) | 2.72 (1.99-3.71) | <0.001 |  |  |
| Top tertile | 129 (27.1) | 128 (26.2) | 1.20 (0.88-1.64) | 0.244 |  |  | 198 (37.1) | 2.47 (1.82-3.35) | <0.001 |  |  |
| **Perspective taking** _[5]_ |  |  |  |  |  |  |  |  |  |  |  |
| Bottom tertile | 232 (48.7) | 178 (36.4) | Ref. |  | Ref. |  | 129 (24.2) | Ref. |  | Ref. |  |
| Middle tertile | 96 (20.2) | 141 (28.8) | 1.91 (1.38-2.65) | <0.001 | 1.64 (1.13-2.35) | 0.008 | 148 (27.8) | 2.77 (1.98-3.88) | <0.001 | 1.88 (1.26-2.80) | 0.002 |
| Top tertile | 148 (31.1) | 170 (34.8) | 1.50 (1.12-2.01) | 0.007 | 1.19 (0.83-0.86) | 0.346 | 256 (48.0) | 3.11 (2.32-4.18) | <0.001 | 1.34 (0.91-1.97) | 0.133 |
| **Conscientiousness** _[6]_ |  |  |  |  |  |  |  |  |  |  |  |
| Bottom tertile | 197 (41.4) | 224 (45.8) | Ref. |  | Ref. |  | 138 (25.9) | Ref. |  | Ref. |  |
| Middle tertile | 134 (28.1) | 138 (28.2) | 0.91 (0.67-1.22) | 0.525 | 0.74 (0.52-1.05) | 0.093 | 179 (33.6) | 1.91 (1.40-2.61) | <0.001 | 0.99 (0.67-1.45) | 0.958 |
| Top tertile | 145 (30.5) | 127 (26.0) | 0.77 (0.57-1.05) | 0.094 | 0.58 (0.39-0.86) | 0.006 | 216 (40.5) | 2.13 (1.57-2.88) | <0.001 | 0.69 (0.46-1.04) | 0.076 |
| **Extroversion** _[6]_ |  |  |  |  |  |  |  |  |  |  |  |
| Bottom tertile | 199 (41.8) | 132 (27.0) | Ref. |  | Ref. |  | 121 (22.7) | Ref. |  | Ref. |  |
| Middle tertile | 164 (34.5) | 221 (45.2) | 2.03 (1.51-2.74) | <0.001 | 1.63 (1.17-2.26) | 0.004 | 179 (33.6) | 1.80 (1.32-2.45) | <0.001 | 1.74 (1.20-2.51) | 0.003 |
| Top tertile | 113 (23.7) | 136 (27.8) | 1.81 (1.30-2.53) | <0.001 | 1.70 (1.17-2.47) | 0.005 | 233 (43.7) | 3.39 (2.47-4.66) | <0.001 | 2.24 (1.53-3.29) | <0.001 |
| **Agreeableness** _[6]_ |  |  |  |  |  |  |  |  |  |  |  |
| Bottom tertile | 247 (51.9) | 222 (45.4) | Ref. |  | Ref. |  | 120 (22.5) | Ref. |  | Ref. |  |
| Middle tertile | 133 (27.9) | 171 (35.0) | 1.43 (1.07-1.91) | 0.016 | 1.35 (0.96-1.90) | 0.081 | 206 (38.7) | 3.19 (2.34-4.34) | <0.001 | 2.16 (1.49-3.12) | <0.001 |
| Top tertile | 96 (20.2) | 96 (19.6) | 1.11 (0.80-1.56) | 0.534 | 1.19 (0.77-1.83) | 0.428 | 207 (38.8) | 4.43 (3.20-6.15) | <0.001 | 2.08 (1.35-3.21) | 0.001 |
| **Openness** _[6]_ |  |  |  |  |  |  |  |  |  |  |  |
| Bottom tertile | 219 (46.0) | 192 (39.3) | Ref. |  |  |  | 172 (32.3) | Ref. |  |  |  |
| Middle tertile | 127 (26.7) | 147 (30.0) | 1.32 (0.97-1.79) | 0.076 |  |  | 161 (30.2) | 1.61 (1.19-2.19) | 0.002 |  |  |
| Top tertile | 130 (27.3) | 150 (30.7) | 1.32 (0.97-1.78) | 0.077 |  |  | 200 (37.5) | 1.96 (1.45-2.64) | <0.001 |  |  |
| **Neuroticism** _[6]_ |  |  |  |  |  |  |  |  |  |  |  |
| Bottom tertile | 147 (30.9) | 124 (25.3) | Ref. |  | Ref. |  | 270 (50.7) | Ref. |  | Ref. |  |
| Middle tertile | 76 (16.0) | 104 (21.3) | 1.62 (1.11-2.37) | 0.013 | 1.74 (1.13-2.66) | 0.011 | 105 (19.7) | 0.75 (0.53-1.07) | 0.118 | 1.26 (0.82-1.94) | 0.292 |
| Top tertile | 253 (53.1) | 261 (53.4) | 1.22 (0.91-1.64) | 0.181 | 1.43 (0.99-2.05) | 0.054 | 158 (29.6) | 0.34 (0.26-0.45) | <0.001 | 0.72 (0.50-1.04) | 0.083 |
| **COVID-19 infection (positive test)** | 26 (5.5) | 43 (8.8) | 1.67 (1.01-2.76) | 0.047 |  |  | 37 (6.9) | 1.29 (0.77-2.17) | 0.333 |  |  |
| **Approval of the COVID-19 measures implemented by the government** |  |  |  |  |  |  |  |  |  |  |  |
| No, they were unnecessary/  unjustified | 90 (18.9) | 58 (11.9) | Ref. |  |  |  | 44 (8.3) | Ref. |  |  |  |
| Yes, partially | 174 (36.6) | 184 (37.6) | 1.64 (1.11-2.42) | 0.013 |  |  | 156 (29.3) | 1.83 (1.20-2.79) | 0.005 |  |  |
| Yes, mainly or totally | 212 (44.5) | 247 (50.5) | 1.81 (1.24-2.64) | 0.002 |  |  | 333 (62.4) | 3.21 (2.15-4.79) | <0.001 |  |  |
| **Vaccinated against COVID-19** |  |  |  |  |  |  |  |  |  |  |  |
| Fully immunized (second shot or Johnson&Johnson) | 321 (67.4) | 354 (72.4) | Ref. |  |  |  | 424 (79.6) | Ref. |  |  |  |
| Partially immunized (first shot) | 29 (6.1) | 34 (7.0) | 1.06 (0.63-1.78) | 0.817 |  |  | 32 (6.00) | 0.84 (0.50-1.41) | 0.500 |  |  |
| Not yet, but made an appointment to get vaccinated | 27 (5.7) | 29 (5.9) | 0.97 (0.56-1.68) | 0.924 |  |  | 17 (3.2) | 0.48 (0.26-0.89) | 0.020 |  |  |
| No, won´t get vaccinated | 99 (20.8) | 72 (14.7) | 0.66 (0.47-0.93) | 0.016 |  |  | 60 (11.2) | 0.46 (0.32-0.65) | <0.001 |  |  |
| **BMI** [kg/m²] _[7]_ |  |  |  |  |  |  |  |  |  |  |  |
| Normal weight [BMI≥18·5 & <25] | 167 (36.6) | 187 (39.9) | Ref. |  |  |  | 203 (38.9) | Ref. |  |  |  |
| Underweight [BMI<18·5] | 4 (0.9) | 5 (1.1) | 1.12 (0.29-4.23) | 0.871 |  |  | 5 (1.0) | 1.03 (0.27-3.89) | 0.967 |  |  |
| Overweight [BMI≥25 & <30] | 176 (38.6) | 183 (39.0) | 0.93 (0.69-1.25) | 0.621 |  |  | 200 (38.3) | 0.93 (0.70-1.25) | 0.647 |  |  |
| Obesity [BMI≥30] | 109 (23.9) | 94 (20.0) | 0.77 (0.54-1.09) | 0.139 |  |  | 114 (21.8) | 0.86 (0.62-1.20) | 0.376 |  |  |
| **Frequency of physical activity done for at least 10 minutes that raises the heartbeat or respiratory rate** |  |  |  |  |  |  |  |  |  |  |  |
| Less than once a week | 127 (26.7) | 77 (15.8) | Ref. |  | Ref. |  | 93 (17.4) | Ref. |  | Ref. |  |
| 1-2 days a week | 108 (22.7) | 134 (27.4) | 2.05 (1.40-2.99) | <0.001 | 1.80 (1.18-2.75) | 0.007 | 116 (21.8) | 1.47 (1.01-2.13) | 0.045 | 1.24 (0.78-1.96) | 0.359 |
| 3-4 days a week | 101 (21.2) | 155 (31.7) | 2.53 (1.73-3.69) | <0.001 | 2.05 (1.33-3.14) | 0.001 | 154 (28.9) | 2.08 (1.44-3.00) | <0.001 | 1.24 (0.79-1.96) | 0.356 |
| 5-7 days a week | 140 (29.4) | 123 (25.1) | 1.45 (1.00-2.10) | 0.051 | 1.19 (0.78-1.81) | 0.422 | 170 (31.9) | 1.66 (1.17-2.35) | 0.004 | 0.80 (0.52-1.25) | 0.332 |
| **Smoking status** |  |  |  |  |  |  |  |  |  |  |  |
| Never | 172 (36.1) | 206 (42.1) | Ref. |  |  |  | 207 (38.8) | Ref. |  |  |  |
| Former | 138 (29.0) | 152 (31.1) | 0.92 (0.68-1.25) | 0.593 |  |  | 163 (30.6) | 0.98 (0.72-1.33) | 0.904 |  |  |
| Current | 166 (34.9) | 131 (26.8) | 0.66 (0.49-0.89) | 0.007 |  |  | 163 (30.6) | 0.82 (0.61-1.10) | 0.178 |  |  |
| **Chronic disease** _[8]_ | 222 (46.6) | 242 (49.5) | 1.12 (0.87-1.44) | 0.376 |  |  | 257 (48.2) | 1.07 (0.83-1.36) | 0.616 |  |  |
| **Depression** (ever) | 85 (17.9) | 62 (12.7) | 0.67 (0.47-0.95) | 0.026 |  |  | 54 (10.1) | 0.52 (0.36-0.75) | <0.001 |  |  |
| **Sleep problems in the last 4 weeks** ^[9]^ |  |  |  |  |  |  |  |  |  |  |  |
| None | 114 (24.0) | 169 (34.6) | Ref. |  | Ref. |  | 193 (36.2) | Ref. |  | Ref. |  |
| Once a week | 20 (4.2) | 29 (5.8) | 0.98 (0.53-1.81) | 0.944 | 0.92 (0.48-1.76) | 0.791 | 49 (9.2) | 1.45 (0.82-2.56) | 0.203 | 1.21 (0.64-2.29) | 0.554 |
| 1-2 times a week | 119 (25.0) | 108 (22.1) | 0.61 (0.43-0.87) | 0.006 | 0.57 (0.39-0.84) | 0.004 | 114 (21.4) | 0.57 (0.40-0.80) | 0.001 | 0.68 (0.45-1.02) | 0.065 |
| 3-4 times a week | 112 (23.5) | 102 (20.9) | 0.61 (0.43-0.88) | 0.008 | 0.60 (0.40-0.87) | 0.010 | 100 (18.8) | 0.53 (0.37-0.75) | <0.001 | 0.74 (0.49-1.12) | 0.158 |
| More than 5 times a week | 111 (23.3) | 81 (16.6) | 0.49 (0.33-0.71) | <0.001 | 0.62 (0.40-0.94) | 0.026 | 77 (14.4) | 0.41 (0.28-0.59) | <0.001 | 0.68 (0.43-1.06) | 0.091 |
| **Duration of sleep problems** (regarding the abovementioned) > 3 months | 246 (68.0) | 198 (61.09) | 0.77 (0.56-1.05) | 0.097 |  |  | 213 (62.6) | 0.79 (0.58-1.08) | 0.140 |  |  |
| **Complex real problems require the collaboration between scientists and practitioners in problem solving** |  |  |  |  |  |  |  |  |  |  |  |
| Do not agree at all or rather not agree | 64 (13.4) | 45 (9.2) | Ref. |  |  |  | 25 (4.7) | Ref. |  |  |  |
| Rather agree | 234 (49.2) | 267 (54.6) | 1.62 (1.07-2.47) | 0.024 |  |  | 220 (41.3) | 2.41 (1.46-3.96) | 0.001 |  |  |
| Agree | 178 (37.4) | 177 (36.2) | 1.41 (0.92-2.18) | 0.118 |  |  | 288 (54.0) | 4.14 (2.52-6.82) | <0.001 |  |  |
| **I have heard of the SDGs and consider them to be important** |  |  |  |  |  |  |  |  |  |  |  |
| Do not agree at all | 137 (28.8) | 101 (20.7) | Ref. |  | Ref. |  | 120 (22.5) | Ref. |  | Ref. |  |
| Rather not agree | 157 (33.0) | 132 (27.0) | 1.14 (0.81-1.61) | 0.456 | 0.96 (0.65-1.40) | 0.817 | 116 (21.8) | 0.84 (0.60-1.19) | 0.331 | 1.03 (0.68-1.55) | 0.893 |
| Rather agree | 150 (31.5) | 200 (40.9) | 1.81 (1.30-2.52) | <0.001 | 1.22 (0.84-1.77) | 0.298 | 199 (37.3) | 1.51 (1.10-2.09) | 0.012 | 1.08 (0.73-1.60) | 0.685 |
| Agree | 32 (6.7) | 56 (11.4) | 2.37 (1.43-3.93) | 0.001 | 1.72 (0.98-3.03) | 0.059 | 98 (18.4) | 3.50 (2.19-5.58) | <0.001 | 2.25 (1.28-3.94) | 0.005 |
| **Conspiracy score** ^[10]^ |  |  |  |  |  |  |  |  |  |  |  |
| Bottom tertile | 165 (34.7) | 185 (37.8) | Ref. |  | Ref. |  | 295 (55.3) | Ref. |  | Ref. |  |
| Middle tertile | 102 (21.4) | 88 (18.0) | 0.77 (0.54-1.10) | 0.147 | 0.69 (0.47-1.02) | 0.065 | 81 (15.2) | 0.44 (0.31-0.63) | <0.001 | 0.52 (0.35-0.78) | 0.002 |
| Top tertile | 209 (43.9) | 216 (44.2) | 0.92 (0.69-1.22) | 0.573 | 0.80 (0.57-1.12) | 0.189 | 157 (29.5) | 0.42 (0.32-0.56) | <0.001 | 0.61 (0.43-0.87) | 0.007 |
| **Complexity score** ^[11]^ |  |  |  |  |  |  |  |  |  |  |  |
| Bottom tertile | 255 (53.6) | 237 (48.5) | Ref. |  | Ref. |  | 181 (34.0) | Ref. |  | Ref. |  |
| Middle tertile | 112 (23.5) | 131 (26.8) | 1.26 (0.92-1.71) | 0.144 | 1.09 (0.78-1.54) | 0.616 | 155 (29.0) | 1.95 (1.43-2.66) | <0.001 | 1.54 (1.07-2.23) | 0.021 |
| Top tertile | 109 (22.9) | 121 (24.7) | 1.19 (0.87-1.63) | 0.267 | 1.01 (0.70-1.47) | 0.938 | 197 (37.0) | 2.55 (1.88-3.44) | <0.001 | 1.83 (1.25-2.69) | 0.002 |
| **Weight loss** |  |  |  |  |  |  |  |  |  |  |  |
| Yes, I have tried losing weight and I lost the weight I wanted to lose | 119 (25.0) | 139 (28.4) | Ref. |  |  |  | 168 (31.5) | Ref. |  |  |  |
| Yes, I have tried losing weight but I have not lost the weight I wanted to lose | 159 (33.4) | 154 (31.5) | 0.83 (0.60-1.15) | 0.266 |  |  | 142 (26.7) | 0.63 (0.46-0.88) | 0.006 |  |  |
| Yes, I have tried losing weight but I have not lost any | 44 (9.2) | 37 (7.6) | 0.72 (0.44-1.19) | 0.199 |  |  | 39 (7.3) | 0.63 (0.38-1.03) | 0.063 |  |  |
| No, I never have tried to lose weight | 154 (32.4) | 159 (32.5) | 0.88 (0.64-1.23) | 0.464 |  |  | 184 (34.5) | 0.85 (0.62-1.16) | 0.303 |  |  |
| [1] mutually adjusted for all variables for which adjusted odds ratios with 95% confidence intervals and adjusted p-values are reported.  [2] citizenship was excluded from multivariable models due to multicollinearity  [3] TKS-WLB^1^  [4] LOT-R^2^  [5] questionnaire for empathy and perspective taking, German version^3^  [6] BFI-S^4^  [7] 51 missing values. Missing indicators were used in multivariable models.  [8] Asthma, COPD, chronical bronchitis, emphysema, heart attack, angina pectoris or coronary heart disease, cancer, hypertension, stroke or diabetes  [9] Report of difficulty initiating sleep and/or difficulty maintaining sleep and/or waking up earlier than desired.  [10] For derivation see supplementary materials  [11] For derivation see supplementary materials | | | | | | | | | | | |

References for Tables:

1 Syrek C, Bauer-Emmel C, Antoni C, Klusemann J. Entwicklung und Validierung der Trierer Kurzskala zur Messung von Work-Life Balance (TKS-WLB). *http://dx.doi.org/101026/0012-1924/a000044* 2011; **57**: 134–45.

2 Hinz A, Sander C, Glaesmer H, *et al.* Optimism and pessimism in the general population: Psychometric properties of the Life Orientation Test (LOT-R). *Int J Clin Heal Psychol* 2017; **17**: 161–70.

3 Maes, Schmitt, Schmal. Fragebogen für Empathie und Perspektivenübernahme. 1995.

4 Gerlitz J-Y, Schupp J. Research Notes Zur Erhebung der Big-Five-basierten Persönlichkeitsmerkmale im SOEP. 2014.
