# Supplementary material for: Determinants of trust in times of crises: A cross-sectional study of 3,065 German-speaking adults from the D-A-CH region
Source: PLoS One. 2023 Oct 12;18(10):e0286488. doi: 10.1371/journal.pone.0286488 (PMC10569553; doi:10.1371/journal.pone.0286488)
Supplement: S2 Table — (DOCX) [file pone.0286488.s003.docx]

| **S2 Table. Factors cross-sectionally associated with interpersonal trust among women (N=1,567).** | | | | | | | | | | | |
| --- | --- | --- | --- | --- | --- | --- | --- | --- | --- | --- | --- |
|  | Interpersonal trust | | | | | | | | | | |
|  | Lowest tertile (N=567) | Middle tertile (N=518) | | | | | Highest tertile  (N=482) | | | | |
|  | N (%) | N (%) | OR_crude_ (95% CI) | p | OR_adj._ (95% CI) ^[1]^ | p ^[1]^ | N (%) | OR_crude_ (95% CI) | p-value | OR_adj._ (95% CI) ^[1]^ | p-value ^[1]^ |
| **Age** |  |  |  |  |  |  |  |  |  |  |  |
| 18-25 | 91 (16.1) | 85 (16.4) | Ref. |  |  |  | 62 (12.9) | Ref. |  |  |  |
| 26-35 | 128 (22.6) | 102 (19.7) | 0.85 (0.58-1.26) | 0.429 |  |  | 76 (15.8) | 0.87 (0.57-1.34) | 0.530 |  |  |
| 36-45 | 96 (16.9) | 106 (20.5) | 1.18 (0.79-1.77) | 0.418 |  |  | 106 (22.0) | 1.62 (1.06-2-48) | 0.026 |  |  |
| 46-55 | 100 (17.6) | 88 (17.0) | 0.94 (0.62-1.42) | 0.777 |  |  | 87 (18.1) | 1.28 (0.83-1.97) | 0.268 |  |  |
| 56-65 | 102 (18.0) | 69 (13.3) | 0.72 (0.47-1.11) | 0.137 |  |  | 83 (17.2) | 1.19 (0.77-1.84) | 0.422 |  |  |
| ≥66 | 50 (8.8) | 68 (13.1) | 1.46 (0.91-2.33) | 0.117 |  |  | 68 (14.1) | 2.00 (1.23-3.25) | 0.005 |  |  |
| **Country of residence** |  |  |  |  |  |  |  |  |  |  |  |
| Austria | 197 (34.7) | 167 (32.2) | Ref. |  |  |  | 157 (32.6) | Ref. |  |  |  |
| Germany | 205 (36.2) | 175 (33.8) | 1.01 (0.75-1.34) | 0.962 |  |  | 151 (31.3) | 0.92 (0.69-1.24) | 0.603 |  |  |
| Switzerland | 165 (29.1) | 176 (34.0) | 1.26 (0.94-1.69) | 0.128 |  |  | 174 (36.1) | 1.32 (0.98-1.78) | 0.066 |  |  |
| **Citizenship** _[2]_ |  |  |  |  |  |  |  |  |  |  |  |
| Austrian | 172 (30.3) | 155 (29.9) | Ref. |  | Ref. |  | 144 (29.9) | Ref. |  | Ref. |  |
| German | 212 (37.4) | 183 (35.3) | 0.96 (0.71-1.28) | 0.774 | 1.01 (0.73-1.39) | 0.956 | 161 (33.4) | 0.91 (0.67-1.23) | 0.526 | 1.23 (0.86-1.77) | 0.251 |
| Swiss | 137 (24.2) | 151 (29.2) | 1.22 (0.89-1.68) | 0.213 | 1.26 (0.88-1.80) | 0.204 | 158 (32.8) | 1.38 (1.00-1.89) | 0.049 | 1.75 (1.17-2.60) | 0.006 |
| Other, EU | 30 (5.3) | 14 (2.7) | 0.52 (0.26-1.01) | 0.054 | 0.77 (0.34-1.74) | 0.534 | 13 (2.7) | 0.52 (0.26-1.03) | 0.060 | 1.24 (0.49-3.10) | 0.652 |
| Other, Non-EU | 16 (2.8) | 15 (2.9) | 1.04 (0.50-2.17) | 0.916 | 1.52 (0.62-3.75) | 0.360 | 6 (1.2) | 0.45 (0.17-1.17) | 0.102 | 0.87 (0.26-2.90) | 0.819 |
| **Ethnicity** |  |  |  |  |  |  |  |  |  |  |  |
| White | 494 (87.1) | 463 (89.4) | Ref. |  |  |  | 447 (92.7) | Ref. |  |  |  |
| Other than white | 72 (12.9) | 55 (10.6) | 0.80 (0.55-1.17) | 0.250 |  |  | 35 (7.3) | 0.53 (0.35-0.81) | 0.003 |  |  |
| **Migration history** |  |  |  |  |  |  |  |  |  |  |  |
| First generation | 149 (26.3) | 158 (30.5) | Ref. |  |  |  | 124 (25.7) | Ref. |  |  |  |
| Second generation | 69 (12.2) | 52 (10.0) | 0.71 (0.47-1.09) | 0.114 |  |  | 40 (8.3) | 0.70 (0.44-1.10) | 0.121 |  |  |
| More than second generation/none | 349 (61.5) | 308 (59.5) | 0.83 (0.63-1.09) | 0.185 |  |  | 318 (66.0) | 1.09 (0.83-1.45) | 0.530 |  |  |
| **Mother tongue** |  |  |  |  |  |  |  |  |  |  |  |
| German | 489 (86.2) | 468 (90.3) | Ref. |  | Ref. |  | 444 (92.1) | Ref. |  | Ref. |  |
| Other than German | 78 (13.8) | 50 (9.7) | 0.67 (0.46-0.98) | 0.037 | 0.57 (0.35-0.95) | 0.030 | 38 (7.9) | 0.54 (0.36-0.81) | 0.003 | 0.56 (0.32-0.99) | 0.048 |
| **Living area** |  |  |  |  |  |  |  |  |  |  |  |
| Urban | 295 (52.0) | 266 (51.3) | Ref. |  |  |  | 228 (47.3) | Ref. |  |  |  |
| Rural | 272 (48.0) | 252 (48.7) | 1.03 (0.81-1.30) | 0.824 |  |  | 254 (52.7) | 1.21 (0.95-1.54) | 0.127 |  |  |
| **Marital status** |  |  |  |  |  |  |  |  |  |  |  |
| Single | 196 (34.6) | 160 (30.9) | Ref. |  |  |  | 147 (30.5) | Ref. |  |  |  |
| Married/partnership | 278 (49.0) | 276 (53.3) | 1.22 (0.93-1.59) | 0.151 |  |  | 268 (55.6) | 1.29 (0.98-1.69) | 0.070 |  |  |
| Divorced | 78 (13.8) | 58 (11.2) | 0.91 (0.61-1.36) | 0.647 |  |  | 49 (10.2) | 0.84 (0.55-1.27) | 0.404 |  |  |
| Widowed | 15 (2.6) | 24 (4.6) | 1.96 (1.00-3.86) | 0.052 |  |  | 18 (3.7) | 1.60 (0.78-3.28) | 0.199 |  |  |
| **Educational attainment** |  |  |  |  |  |  |  |  |  |  |  |
| No university degree | 464 (81.8) | 411 (79.3) | Ref. |  |  |  | 347 (72.0) | Ref. |  |  |  |
| University degree | 103 (18.2) | 107 (20.7) | 1.17 (0.87-1.59) | 0.073 |  |  | 135 (28.0) | 1.75 (1.31-2.35) | <0.001 |  |  |
| **Household income** |  |  |  |  |  |  |  |  |  |  |  |
| Bottom tertile | 300 (52.9) | 230 (44.4) | Ref. |  |  |  | 172 (35.7) | Ref. |  |  |  |
| Middle tertile | 122 (21.5) | 135 (26.1) | 1.44 (1.07-1.95) | 0.016 |  |  | 134 (27.8) | 1.92 (1.41-2.61) | <0.001 |  |  |
| Highest tertile | 145 (25.6) | 153 (29.5) | 1.38 (1.04-1.83) | 0.028 |  |  | 176 (36.5) | 2.12 (1.59-2.83) | <0.001 |  |  |
| **Work status** |  |  |  |  |  |  |  |  |  |  |  |
| Full- (part-) time employed | 223 (39.3) | 202 (39.0) | Ref. |  |  |  | 196 (40.7) | Ref. |  |  |  |
| Full- (part-) time self-employed | 34 (6.0) | 28 (5.4) | 0.91 (0.53-1.55) | 0.727 |  |  | 29 (6.0) | 0.97 (0.57-1.65) | 0.912 |  |  |
| Unemployed | 36 (6.4) | 31 (6.0) | 0.95 (0.57-1.59) | 0.848 |  |  | 23 (4.8) | 0.73 (0.42-1.27) | 0.262 |  |  |
| Retired | 93 (16.4) | 110 (21.2) | 1.31 (0.93-1.83) | 0.119 |  |  | 89 (18.5) | 1.09 (0.77-1.54) | 0.632 |  |  |
| Student/in training/civil-/military-service | 44 (7.8) | 46 (8.9) | 1.15 (0.73-1.82) | 0.537 |  |  | 44 (9.1) | 1.14 (0.72-1.80) | 0.582 |  |  |
| Household | 48 (8.5) | 41 (7.9) | 0.94 (0.60-1.49) | 0.802 |  |  | 42 (8.7) | 1.00 (0.63-1.57) | 0.985 |  |  |
| Temporary contract | 13 (2.3) | 6 (1.2) | 0.51 (0.19-1.37) | 0.180 |  |  | 9 (1.9) | 0.79 (0.33-1.88) | 0.591 |  |  |
| Permanent contract | 76 (13.4) | 54 (10.4) | 0.78 (0.53-1.17) | 0.231 |  |  | 50 (10.4) | 0.75 (0.50-1.12) | 0.161 |  |  |
| **Satisfaction with work** |  |  |  |  |  |  |  |  |  |  |  |
| No, does not or does rather not apply | 203 (35.8) | 149 (28.8) | Ref. |  |  |  | 95 (19.7) | Ref. |  |  |  |
| Yes, does rather apply | 260 (45.9) | 269 (51.9) | 1.41 (1.07-1.85) | 0.013 |  |  | 242 (50.2) | 1.99 (1.47-2.68) | <0.001 |  |  |
| Yes, does totally apply | 104 (18.3) | 100 (19.3) | 1.31 (0.93-1.85) | 0.127 |  |  | 145 (30.1) | 2.98 (2.10-4.23) | <0.001 |  |  |
| **Work-Life balance** ^[3]^ |  |  |  |  |  |  |  |  |  |  |  |
| Bottom tertile | 245 (43.2) | 183 (35.3) | Ref. |  |  |  | 109 (22.6) | Ref. |  |  |  |
| Middle tertile | 161 (28.4) | 176 (34.0) | 1.46 (1.10-1.95) | 0.009 |  |  | 165 (34.2) | 2.30 (1.68-3.15) | <0.001 |  |  |
| Top tertile | 161 (28.4) | 159 (30.7) | 1.32 (0.99-1.77) | 0.060 |  |  | 208 (43.2) | 2.90 (2.14-3.94) | <0.001 |  |  |
| **Political preference** (last elections) |  |  |  |  |  |  |  |  |  |  |  |
| Did not vote | 251 (44.3) | 208 (40.2) | Ref. |  | Ref. |  | 133 (27.6) | Ref. |  | Ref. |  |
| Opposition parties | 120 (21.1) | 79 (15.2) | 0.79 (0.57-1.11) | 0.182 | 0.77 (0.53-1.12) | 0.167 | 96 (19.9) | 1.51 (1.07-2.12) | 0.018 | 1.27 (0.83-1.95) | 0.278 |
| Governing parties | 196 (34.6) | 231 (44.6) | 1.42 (1.09-1.85) | 0.009 | 1.22 (0.90-1.66) | 0.207 | 253 (52.5) | 2.44 (1.84-3.23) | <0.001 | 1.57 (1.10-2.25) | 0.013 |
| **Participation at religious meetings** |  |  |  |  |  |  |  |  |  |  |  |
| At least once a month | 52 (9.2) | 78 (15.0) | Ref. |  | Ref. |  | 51 (10.6) | Ref. |  | Ref. |  |
| Less than once a month | 71 (12.5) | 77 (14.9) | 0.72 (0.45-1.16) | 0.182 | 0.68 (0.41-1.14) | 0.145 | 99 (20.5) | 1.42 (0.87-2.33) | 0.161 | 1.08 (0.60-1.95) | 0.787 |
| Never, or almost never | 444 (78.3) | 363 (70.1) | 0.55 (0.37-0.79) | 0.002 | 0.52 (0.34-0.80) | 0.003 | 332 (68.9) | 0.76 (0.51-1.15) | 0.196 | 0.56 (0.34-0.93) | 0.025 |
| **Contact with a close person (except children)** |  |  |  |  |  |  |  |  |  |  |  |
| Less than once a week | 55 (9.7) | 54 (10.4) | Ref. |  |  |  | 16 (3.3) | Ref. |  |  |  |
| At least once a week | 110 (19.4) | 91 (17.6) | 0.84 (0.53-1.34) | 0.472 |  |  | 97 (20.1) | 3.03 (1.63-5.64) | <0.001 |  |  |
| Daily | 402 (70.9) | 373 (72.0) | 0.95 (0.63-1.41) | 0.782 |  |  | 369 (76.6) | 3.16 (1.78-5.60) | <0.001 |  |  |
| **In conversations I consider myself a:** |  |  |  |  |  |  |  |  |  |  |  |
| *“No, but…” type* | 203 (35.8) | 147 (28.4) | Ref. |  |  |  | 108 (22.4) | Ref. |  |  |  |
| *“Yes, and…” type* | 364 (64.2) | 371 (71.6) | 1.41 (1.09-1.82) | 0.009 |  |  | 374 (77.6) | 1.93 (1.47-2.54) | <0.001 |  |  |
| **Optimism** _[4]_ |  |  |  |  |  |  |  |  |  |  |  |
| Bottom tertile | 329 (58.0) | 204 (39.4) | Ref. |  | Ref. |  | 83 (17.2) | Ref. |  | Ref. |  |
| Middle tertile | 134 (23.6) | 161 (31.1) | 1.94 (1.45-2.58) | <0.001 | 2.00 (1.46-2.73) | <0.001 | 100 (20.8) | 2.96 (2.08-4.21) | <0.001 | 2.62 (1.77-3.86) | <0.001 |
| Top tertile | 104 (18.4) | 153 (29.5) | 2.37 (1.75-3.22) | <0.001 | 2.59 (1.80-3.73) | <0.001 | 299 (62.0) | 11.40 (8.21-15.8) | <0.001 | 8.61 (5.78-12.9) | <0.001 |
| **Empathy** _[5]_ |  |  |  |  |  |  |  |  |  |  |  |
| Bottom tertile | 171 (30.2) | 138 (26.6) | Ref. |  |  |  | 79 (16.4) | Ref. |  |  |  |
| Middle tertile | 147 (25.9) | 158 (30.5) | 1.33 (0.97-1.83) | 0.077 |  |  | 152 (31.5) | 2.23 (1.58-3.18) | <0.001 |  |  |
| Top tertile | 249 (43.9) | 222 (42.9) | 1.10 (0.83-1.47) | 0.498 |  |  | 251 (52.1) | 2.18 (1.59-3.00) | <0.001 |  |  |
| **Perspective taking** _[5]_ |  |  |  |  |  |  |  |  |  |  |  |
| Bottom tertile | 214 (37.7) | 155 (29.9) | Ref. |  | Ref. |  | 93 (19.3) | Ref. |  | Ref. |  |
| Middle tertile | 140 (24.7) | 150 (29.0) | 1.48 (1.09-2.02) | 0.013 | 1.29 (0.92-1.81) | 0.134 | 135 (28.0) | 2.22 (1.58-3.12) | <0.001 | 1.57 (1.05-2.35) | 0.028 |
| Top tertile | 213 (37.6) | 213 (41.1) | 1.38 (1.04-1.83) | 0.024 | 1.32 (0.95-1.84) | 0.098 | 254 (52.7) | 2.74 (2.02-3.72) | <0.001 | 1.78 (1.21-2.61) | 0.003 |
| **Conscientiousness** _[6]_ |  |  |  |  |  |  |  |  |  |  |  |
| Bottom tertile | 206 (36.3) | 205 (39.6) | Ref. |  | Ref. |  | 97 (20.1) | Ref. |  | Ref. |  |
| Middle tertile | 143 (25.2) | 145 (28.0) | 1.02 (0.75-1.38) | 0.903 | 0.75 (0.53-1.05) | 0.095 | 156 (32.4) | 2.32 (1.66-3.23) | <0.001 | 1.06 (0.71-1.59) | 0.767 |
| Top tertile | 218 (38.5) | 168 (32.4) | 0.77 (0.59-1.02) | 0.073 | 0.51 (0.36-0.73) | <0.001 | 229 (47.5) | 2.23 (1.65-3.02) | <0.001 | 0.69 (0.46-1.03) | 0.071 |
| **Extroversion** _[6]_ |  |  |  |  |  |  |  |  |  |  |  |
| Bottom tertile | 211 (37.2) | 143 (27.6) | Ref. |  | Ref. |  | 110 (22.8) | Ref. |  | Ref. |  |
| Middle tertile | 174 (30.7) | 202 (39.0) | 1.71 (1.28-2.30) | <0.001 | 1.79 (1.30-2.46) | <0.001 | 139 (28.8) | 1.53 (1.11-2.11) | 0.009 | 1.80 (1.23-2.63) | 0.003 |
| Top tertile | 182 (32.1) | 173 (33.4) | 1.40 (1.04-1.89) | 0.026 | 1.36 (0.98-1.90) | 0.069 | 233 (48.4) | 2.46 (1.82-3.32) | <0.001 | 1.84 (1.28-2.66) | 0.001 |
| **Agreeableness** _[6]_ |  |  |  |  |  |  |  |  |  |  |  |
| Bottom tertile | 244 (43.0) | 180 (34.8) | Ref. |  | Ref. |  | 100 (20.8) | Ref. |  | Ref. |  |
| Middle tertile | 171 (30.2) | 180 (34.8) | 1.43 (1.07-1.90) | 0.014 | 1.41 (1.02-1.95) | 0.036 | 164 (34.0) | 2.34 (1.71-3.21) | <0.001 | 1.53 (1.05-2.24) | 0.026 |
| Top tertile | 152 (26.8) | 158 (30.4) | 1.41 (1.05-1.89) | 0.022 | 1.53 (1.07-2.21) | 0.021 | 218 (45.2) | 3.50 (2.56-4.78) | <0.001 | 1.80 (1.20-2.70) | 0.005 |
| **Openness** _[6]_ |  |  |  |  |  |  |  |  |  |  |  |
| Bottom tertile | 229 (40.4) | 192 (37.1) | Ref. |  |  |  | 128 (26.6) | Ref. |  |  |  |
| Middle tertile | 157 (27.7) | 151 (29.1) | 1.15 (0.85-1.54) | 0.361 |  |  | 153 (31.7) | 1.74 (1.28-2.38) | <0.001 |  |  |
| Top tertile | 181 (31.9) | 175 (33.8) | 1.15 (0.87-1.53) | 0.323 |  |  | 201 (41.7) | 1.99 (1.48-2.67) | <0.001 |  |  |
| **Neuroticism** _[6]_ |  |  |  |  |  |  |  |  |  |  |  |
| Bottom tertile | 114 (20.1) | 101 (19.5) | Ref. |  | Ref. |  | 165 (34.2) | Ref. |  | Ref. |  |
| Middle tertile | 80 (14.1) | 99 (19.1) | 1.40 (0.94-2.08) | 0.100 | 1.56 (1.00-2.43) | 0.049 | 79 (16.4) | 0.68 (0.46-1.01) | 0.056 | 1.16 (0.72-1.88) | 0.541 |
| Top tertile | 373 (65.8) | 318 (61.4) | 0.96 (0.71-1.31) | 0.806 | 1.31 (0.91-1.88 | 0.147 | 238 (49.4) | 0.44 (0.33-0.59) | <0.001 | 1.21 (0.83-1.77) | 0.331 |
| **COVID-19 infection (positive test)** | 41 (97.2) | 38 (7.3) | 1.02 (0.64-1.61) | 0.947 |  |  | 31 (6.4) | 0.88 (0.54-1.43) | 0.610 |  |  |
| **Approval of the COVID-19 measures implemented by the government** |  |  |  |  |  |  |  |  |  |  |  |
| No, they were unnecessary/  unjustified | 109 (19.2) | 77 (14.9) | Ref. |  | Ref. |  | 42 (8.7) | Ref. |  | Ref. |  |
| Yes, partially | 220 (38.8) | 205 (39.6) | 1.32 (0.93-1.87) | 0.119 | 1.13 (0.77-1.65) | 0.533 | 137 (28.4) | 1.62 (1.07-2.45) | 0.023 | 1.43 (0.87-2.34) | 0.154 |
| Yes, mainly or totally | 238 (42.0) | 236 (45.7) | 1.40 (1.00-1.98) | 0.053 | 1.04 (0.70-1.56) | 0.831 | 303 (62.9) | 3.30 (2.23-4.90) | <0.001 | 1.98 (1.20-3.25) | 0.007 |
| **Vaccinated against COVID-19** |  |  |  |  |  |  |  |  |  |  |  |
| Fully immunized (second shot or Johnson&Johnson) | 332 (58.5) | 314 (60.6) | Ref. |  |  |  | 341 (70.8) | Ref. |  |  |  |
| Partially immunized (first shot) | 43 (7.6) | 47 (9.1) | 1.16 (0.74-1.80) | 0.521 |  |  | 30 (6.2) | 0.68 (0.42-1.11) | 0.122 |  |  |
| Not yet, but made an appointment to get vaccinated | 39 (6.9) | 34 (6.6) | 0.92 (0.57-1.50) | 0.742 |  |  | 32 (6.6) | 0.80 (0.49-1.31) | 0.370 |  |  |
| No, won´t get vaccinated | 153 (27.0) | 123 (23.7) | 0.85 (0.64-1.13) | 0.261 |  |  | 79 (16.4) | 0.50 (0.37-0.69) | <0.001 |  |  |
| **BMI** [kg/m²] _[7]_ |  |  |  |  |  |  |  |  |  |  |  |
| Normal weight [BMI≥18·5 & <25] | 247 (50.9) | 240 (52.5) | Ref. |  |  |  | 234 (53.5) | Ref. |  |  |  |
| Underweight [BMI<18·5] | 32 (6.6) | 29 (6.4) | 0.93 (0.54-1.59) | 0.798 |  |  | 21 (4.8) | 0.69 (0.39-1.24) | 0.214 |  |  |
| Overweight [BMI≥25 & <30] | 113 (23.3) | 118 (25.8) | 1.07 (0.79-1.47) | 0.652 |  |  | 114 (26.1) | 1.06 (0.78-1.46) | 0.696 |  |  |
| Obesity [BMI≥30] | 93 (19.2) | 70 (15.3) | 0.77 (0.54-1.11) | 0.161 |  |  | 68 (15.6) | 0.78 (0.54-1.11) | 0.159 |  |  |
| **Frequency of physical activity done for at least 10 minutes that raises the heartbeat or respiratory rate** |  |  |  |  |  |  |  |  |  |  |  |
| Less than once a week | 149 (26.3) | 100 (19.3) | Ref. |  |  |  | 76 (15.8) | Ref. |  |  |  |
| 1-2 days a week | 161 (28.4) | 146 (28.2) | 1.35 (0.96-1.89) | 0.081 |  |  | 136 (28.2) | 1.66 (1.16-2.37) | 0.006 |  |  |
| 3-4 days a week | 142 (25.0) | 129 (24.9) | 1.35 (0.96-1.92) | 0.088 |  |  | 129 (26.8) | 1.78 (1.24-2.57) | 0.002 |  |  |
| 5-7 days a week | 115 (20.3) | 143 (27.6) | 1.85 (1.30-2.64) | 0.001 |  |  | 141 (29.2) | 2.40 (1.66-3.48) | <0.001 |  |  |
| **Smoking status** |  |  |  |  |  |  |  |  |  |  |  |
| Never | 213 (37.6) | 237 (45.8) | Ref. |  | Ref. |  | 247 (51.3) | Ref. |  | Ref. |  |
| Former | 131 (23.1) | 130 (25.1) | 0.89 (0.66-1.21) | 0.462 | 0.88 (0.63-1.21) | 0.424 | 125 (25.9) | 0.82 (0.61-1.12) | 0.212 | 0.73 (0.51-1.05) | 0.092 |
| Current | 223 (39.3) | 151 (29.1) | 0.61 (0.46-0.80) | <0.001 | 0.63 (0.47-0.85) | 0.003 | 110 (22.8) | 0.43 (0.32-0.57) | <0.001 | 0.44 (0.31-0.63) | <0.001 |
| **Chronic disease** _[8]_ | 199 (35.1) | 155 (29.9) | 0.79 (0.61-1.02) | 0.070 |  |  | 168 (34.9) | 0.99 (0.77-1.28) | 0.935 |  |  |
| **Depression** (ever) | 140 (24.7) | 97 (18.7) | 0.70 (0.52-0.94) | 0.018 |  |  | 69 (14.3) | 0.51 (0.37-0.70) | <0.001 |  |  |
| **Sleep problems in the last 4 weeks** ^[9]^ |  |  |  |  |  |  |  |  |  |  |  |
| None | 92 (16.2) | 116 (22.4) | Ref. |  | Ref. |  | 125 (25.9) | Ref. |  | Ref. |  |
| Once a week | 20 (3.6) | 20 (3.9) | 0.79 (0.40-1.56) | 0.502 | 0.75 (0.37-1.55) | 0.441 | 28 (5.8) | 1.03 (0.55-1.94) | 0.926 | 0.86 (0.40-1.83) | 0.698 |
| 1-2 times a week | 127 (22.4) | 124 (23.9) | 0.77 (0.54-1.12) | 0.174 | 0.71 (0.48-1.06) | 0.096 | 119 (24.7) | 0.69 (0.48-1.00) | 0.047 | 0.75 (0.48-1.16) | 0.199 |
| 3-4 times a week | 160 (28.2) | 129 (24.9) | 0.63 (0.45-0.92) | 0.015 | 0.63 (0.43-0.92) | 0.018 | 127 (26.4) | 0.58 (0.41-0.83) | 0.003 | 0.67 (0.44-1.02) | 0.062 |
| More than 5 times a week | 168 (29.6) | 129 (24.9) | 0.61 (0.43-0.87) | 0.006 | 0.67 (0.45-0.98) | 0.039 | 83 (17.2) | 0.36 (0.25-0.53) | <0.001 | 0.44 (0.28-0.69) | <0.001 |
| **Duration of sleep problems** (regarding the abovementioned) > 3 months | 323 (68.0) | 227 (56.5) | 0.61 (0.46-0.80) | <0.001 |  |  | 213 (59.7) | 0.70 (0.52-0.93) | 0.013 |  |  |
| **Complex real problems require the collaboration between scientists and practitioners in problem solving** |  |  |  |  |  |  |  |  |  |  |  |
| Do not agree at all or rather not agree | 87 (15.3) | 59 (11.4) | Ref. |  | Ref. |  | 22 (4.6) | Ref. |  | Ref. |  |
| Rather agree | 304 (53.6) | 315 (60.8) | 1.53 (1.06-2.20) | 0.023 | 1.48 (0.99-2.21) | 0.058 | 254 (52.7) | 3.30 (2.01-5.43) | <0.001 | 2.19 (1.23-3.89) | 0.008 |
| Agree | 176 (31.1) | 144 (27.8) | 1.21 (0.81-1.79) | 0.354 | 0.61 (0.44-0.85) | 0.413 | 206 (42.7) | 4.63 (2.78-7.70) | <0.001 | 2.27 (1.24-4.18) | 0.008 |
| **I have heard of the SDGs and consider them to be important** |  |  |  |  |  |  |  |  |  |  |  |
| Do not agree at all | 145 (25.6) | 98 (18.9) | Ref. |  |  |  | 105 (21.8) | Ref. |  |  |  |
| Rather not agree | 190 (33.5) | 177 (34.2) | 1.38 (0.99-1.91) | 0.055 |  |  | 122 (25.3) | 0.89 (0.63-1.24) | 0.487 |  |  |
| Rather agree | 189 (33.3) | 209 (40.4) | 1.64 (1.18-2.26) | 0.003 |  |  | 185 (38.4) | 1.35 (0.98-1.87) | 0.067 |  |  |
| Agree | 43 (7.6) | 34 (6.5) | 1.17 (0.70-1.96) | 0.552 |  |  | 70 (14.5) | 2.25 (1.43-3.54) | <0.001 |  |  |
| **Conspiracy score** ^[10]^ |  |  |  |  |  |  |  |  |  |  |  |
| Bottom tertile | 155 (27.3) | 188 (36.3) | Ref. |  | Ref. |  | 260 (53.9) | Ref. |  | Ref. |  |
| Middle tertile | 110 (19.4) | 91 (17.6) | 0.68 (0.48-0.97) | 0.032 | 0.65 (0.44-0.94) | 0.024 | 95 (19.7) | 0.51 (0.37-0.72) | <0.001 | 0.51 (0.34-0.76) | 0.001 |
| Top tertile | 302 (53.3) | 239 (46.1) | 0.65 (0.50-0.86) | 0.002 | 0.61 (0.44-0.85) | 0.003 | 127 (26.4) | 0.25 (0.19-0.33) | <0.001 | 0.38 (0.27-0.56) | <0.001 |
| **Complexity score** ^[11]^ |  |  |  |  |  |  |  |  |  |  |  |
| Bottom tertile | 211 (37.2) | 193 (37.3) | Ref. |  |  |  | 121 (25.1) | Ref. |  |  |  |
| Middle tertile | 177 (31.2) | 161 (31.1) | 0.99 (0.74-1.33) | 0.970 |  |  | 155 (32.2) | 1.53 (1.12-2.08) | 0.008 |  |  |
| Top tertile | 179 (31.6) | 164 (31.6) | 1.01 (0.75-1.34) | 0.991 |  |  | 206 (42.7) | 2.01 (1.49-2.71) | <0.001 |  |  |
| **Weight loss** |  |  |  |  |  |  |  |  |  |  |  |
| Yes, I have tried losing weight and I lost the weight I wanted to lose | 187 (33.0) | 168 (32.4) | Ref. |  |  |  | 172 (35.7) | Ref. |  |  |  |
| Yes, I have tried losing weight but I have not lost the weight I wanted to lose | 196 (34.6) | 179 (34.6) | 1.02 (0.76-1.36) | 0.912 |  |  | 173 (35.9) | 0.96 (0.71-1.28) | 0.781 |  |  |
| Yes, I have tried losing weight but I have not lost any | 56 (9.8) | 52 (10.0) | 1.04 (0.67-1.59) | 0.881 |  |  | 40 (8.3) | 0.78 (0.49-1.22) | 0.277 |  |  |
| No, I never have tried to lose weight | 128 (22.6) | 119 (23.0) | 1.03 (0.75-1.43) | 0.836 |  |  | 97 (20.1) | 0.82 (0.59-1.15) | 0.258 |  |  |
| [1] mutually adjusted for all variables for which adjusted odds ratios with 95% confidence intervals and adjusted p-values are reported.  [2] citizenship was excluded from multivariable models due to multicollinearity  [3] TKS-WLB^1^  [4] LOT-R^2^  [5] questionnaire for empathy and perspective taking, German version^3^  [6] BFI-S^4^  [7] 188 missing values. Missing indicators were used in multivariable models.  [8] Asthma, COPD, chronical bronchitis, emphysema, heart attack, angina pectoris or coronary heart disease, cancer, hypertension, stroke or diabetes  [9] Report of difficulty initiating sleep and/or difficulty maintaining sleep and/or waking up earlier than desired.  [10] For derivation see supplementary materials  [11] For derivation see supplementary materials | | | | | | | | | | | |

References for Tables:

1 Syrek C, Bauer-Emmel C, Antoni C, Klusemann J. Entwicklung und Validierung der Trierer Kurzskala zur Messung von Work-Life Balance (TKS-WLB). *http://dx.doi.org/101026/0012-1924/a000044* 2011; **57**: 134–45.

2 Hinz A, Sander C, Glaesmer H, *et al.* Optimism and pessimism in the general population: Psychometric properties of the Life Orientation Test (LOT-R). *Int J Clin Heal Psychol* 2017; **17**: 161–70.

3 Maes, Schmitt, Schmal. Fragebogen für Empathie und Perspektivenübernahme. 1995.

4 Gerlitz J-Y, Schupp J. Research Notes Zur Erhebung der Big-Five-basierten Persönlichkeitsmerkmale im SOEP. 2014.
