# Supplementary material for: Determinants of trust in times of crises: A cross-sectional study of 3,065 German-speaking adults from the D-A-CH region
Source: PLoS One. 2023 Oct 12;18(10):e0286488. doi: 10.1371/journal.pone.0286488 (PMC10569553; doi:10.1371/journal.pone.0286488)
Supplement: S3 Table — (DOCX) [file pone.0286488.s004.docx]

| **S3 Table.** **Factors cross-sectionally associated with interpersonal trust in Austria (N=1,019).** | | | | | | | | | | | |
| --- | --- | --- | --- | --- | --- | --- | --- | --- | --- | --- | --- |
|  | Interpersonal trust | | | | | | | | | | |
|  | Lowest tertile (N=371) | Middle tertile (N=329) | | | | | Highest tertile  (N=319) | | | | |
|  | N (%) | N (%) | OR_crude_ (95% CI) | p | OR_adj._ (95% CI) ^[1]^ | p ^[1]^ | N (%) | OR_crude_ (95% CI) | p-value | OR_adj._ (95% CI) ^[1]^ | p-value ^[1]^ |
| **Age** |  |  |  |  |  |  |  |  |  |  |  |
| 18-25 | 43 (11.6) | 43 (13.1) | Ref. |  |  |  | 43 (13.5) | Ref. |  |  |  |
| 26-35 | 65 (17.5) | 54 (16.4) | 0.83 (0.48-1.45) | 0.513 |  |  | 38 (11.9) | 0.59 (0.33-1.05) | 0.071 |  |  |
| 36-45 | 72 (19.4) | 57 (17.3) | 0.79 (0.46-1.37) | 0.403 |  |  | 58 (18.2) | 0.81 (0.47-1.39) | 0.438 |  |  |
| 46-55 | 68 (18.3) | 59 (17.9) | 0.87 (0.50-1.50) | 0.612 |  |  | 70 (21.9) | 1.03 (0.60-1.76) | 0.916 |  |  |
| 56-65 | 70 (18.9) | 66 (20.1) | 0.94 (0.55-1.62) | 0.831 |  |  | 62 (19.4) | 0.89 (0.51-1.53) | 0.662 |  |  |
| ≥66 | 53 (14.3) | 50 (15.2) | 0.94 (0.53-1.67) | 0.842 |  |  | 48 (15.1) | 0.91 (0.51-1.61) | 0.736 |  |  |
| **Men** | 197 (53.1) | 167 (50.8) | Ref. |  |  |  | 157 (49.2) | Ref. |  |  |  |
| **Women** | 174 (46.9) | 162 (49.2) | 1.10 (0.82-1.48) | 0.536 |  |  | 162 (50.8) | 1.17 (0.87-1.58) | 0.309 |  |  |
| **Ethnicity** |  |  |  |  |  |  |  |  |  |  |  |
| White | 335 (90.3) | 302 (91.8) | Ref. |  |  |  | 294 (92.2) | Ref. |  |  |  |
| Other than white | 36 (9.7) | 27 (8.2) | 0.83 (0.49-1.40) | 0.490 |  |  | 25 (7.8) | 0.79 (0.46-1.35) | 0.390 |  |  |
| **Migration history** |  |  |  |  |  |  |  |  |  |  |  |
| First generation | 87 (23.5) | 80 (24.3) | Ref. |  | Ref. |  | 57 (17.9) | Ref. |  | Ref. |  |
| Second generation | 45 (12.1) | 22 (6.7) | 0.53 (0.29-0.96) | 0.037 | 0.46 (0.24-0.88) | 0.019 | 25 (7.8) | 0.85 (0.47-1.53) | 0.585 | 0.76 (0.37-1.55) | 0.450 |
| More than second generation/none | 239 (64.4) | 227 (69.0) | 1.03 (0.73-1.47) | 0.858 | 0.99 (0.67-1.46) | 0.950 | 237 (74.3) | 1.51 (1.04-2.21) | 0.032 | 1.04 (0.66-1.63) | 0.873 |
| **Mother tongue** |  |  |  |  |  |  |  |  |  |  |  |
| German | 328 (88.4) | 303 (92.1) | Ref. |  |  |  | 296 (92.8) | Ref. |  |  |  |
| Other than German | 43 (11.6) | 26 (7.9) | 0.65 (0.39-1.09) |  |  |  | 23 (7.2) | 0.59 (0.35-1.01) | 0.053 |  |  |
| **Living area** ^[2]^ |  |  |  |  |  |  |  |  |  |  |  |
| Urban | 198 (53.4) | 204 (62.0) | Ref. |  | Ref. |  | 156 (48.9) | Ref. |  | Ref. |  |
| Rural | 173 (46.6) | 125 (38.0) | 0.70 (0.52-0.95) | 0.021 | 0.66 (0.47-0.92) | 0.014 | 163 (51.1) | 1.20 (0.89-1.61) | 0.242 | 1.20 (0.83-1.73) | 0.328 |
| **Marital status** |  |  |  |  |  |  |  |  |  |  |  |
| Single | 107 (28.9) | 91 (27.7) | Ref. |  |  |  | 83 (26.0) | Ref. |  |  |  |
| Married/partnership | 212 (57.1) | 194 (59.0) | 1.08 (0.77-1.51) | 0.673 |  |  | 197 (61.8) | 1.20 (0.85-1.69) | 0.306 |  |  |
| Divorced | 43 (11.6) | 39 (11.8) | 1.07 (0.64-1.79) | 0.807 |  |  | 29 (9.1) | 0.87 (0.50-1.51) | 0.619 |  |  |
| Widowed | 9 (2.4) | 5 (1.5) | 0.65 (0.21-2.02) | 0.460 |  |  | 10 (3.1) | 1.43 (0.56-3.69) | 0.456 |  |  |
| **Educational attainment** |  |  |  |  |  |  |  |  |  |  |  |
| No university degree | 304 (81.9) | 266 (80.9) | Ref. |  |  |  | 249 (78.1) | Ref. |  |  |  |
| University degree | 67 (18.1) | 63 (19.1) | 1.07 (0.73-1.57) | 0.711 |  |  | 70 (21.9) | 1.28 (0.88-1.86) | 0.203 |  |  |
| **Household income** |  |  |  |  |  |  |  |  |  |  |  |
| Bottom tertile | 170 (45.8) | 147 (44.7) | Ref. |  | Ref. |  | 93 (29.1) | Ref. |  | Ref. |  |
| Middle tertile | 84 (22.6) | 80 (24.3) | 1.10 (0.76-1.61) | 0.616 | 0.89 (0.59-1.35) | 0.585 | 71 (22.3) | 1.55 (1.03-2.32) | 0.035 | 1.10 (0.69-1.77) | 0.686 |
| Highest tertile | 117 (31.6) | 102 (31.0) | 1.01 (0.71-1.42) | 0.963 | 0.75 (0.50-1.10) | 0.139 | 155 (48.6) | 2.42 (1.71-3.43) | <0.001 | 1.54 (1.01-2.34) | 0.045 |
| **Work status** |  |  |  |  |  |  |  |  |  |  |  |
| Full- (part-) time employed | 140 (37.7) | 118 (35.9) | Ref. |  |  |  | 125 (39.2) | Ref. |  |  |  |
| Full- (part-) time self-employed | 24 (6.5) | 23 (7.0) | 1.14 (0.61-2.12) | 0.686 |  |  | 25 (7.8) | 1.17 (0.63-2.15) | 0.620 |  |  |
| Unemployed | 26 (7.0) | 25 (7.6) | 1.14 (0.63-2.08) | 0.668 |  |  | 16 (5.0) | 0.69 (0.35-1.34) | 0.275 |  |  |
| Retired | 98 (26.4) | 91 (27.7) | 1.10 (0.76-1.60) | 0.614 |  |  | 74 (23.2) | 0.85 (0.57-1.25) | 0.395 |  |  |
| Student/in training/civil-/military-service | 21 (5.7) | 22 (6.7) | 1.24 (0.65-2.37) | 0.509 |  |  | 27 (8.5) | 1.44 (0.78-2.67) | 0.248 |  |  |
| Household | 13 (3.5) | 10 (3.0) | 0.91 (0.39-2.16) | 0.835 |  |  | 8 (2.5) | 0.69 (0.28-1.72) | 0.424 |  |  |
| Temporary contract | 8 (2.2) | 4 (1.3) | 0.59 (0.17-2.02) | 0.403 |  |  | 4 (1.3) | 0.56 (0.16-1.90) | 0.353 |  |  |
| Permanent contract | 41 (11.1) | 40 (12.5) | 1.04 (0.63-1.74) | 0.875 |  |  | 40 (12.5) | 1.09 (0.66-1.80) | 0.727 |  |  |
| **Satisfaction with work** |  |  |  |  |  |  |  |  |  |  |  |
| No, does not or does rather not apply | 142 (38.3) | 96 (29.2) | Ref. |  |  |  | 61 (19.1) | Ref. |  |  |  |
| Yes, does rather apply | 154 (41.5) | 162 (49.2) | 1.56 (1.11-2.19) | 0.011 |  |  | 156 (48.9) | 2.36 (1.62-3.43) | <0.001 |  |  |
| Yes, does totally apply | 75 (20.2) | 71 (21.6) | 1.40 (0.92-2.12) | 0.112 |  |  | 102 (32.0) | 3.17 (2.07-4.83) | <0.001 |  |  |
| **Work-Life balance** ^[3]^ |  |  |  |  |  |  |  |  |  |  |  |
| Bottom tertile | 175 (47.2) | 119 (36.2) | Ref. |  | Ref. |  | 69 (21.6) | Ref. |  | Ref. |  |
| Middle tertile | 88 (23.7) | 106 (32.2) | 1.77 (1.23-2.55) | 0.002 | 1.62 (1.07-2.45) | 0.022 | 97 (30.4) | 2.80 (1.87-4.18) | <0.001 | 1.66 (1.03-2.68) | 0.037 |
| Top tertile | 108 (29.1) | 104 (31.6) | 1.42 (0.99-2.02) | 0.055 | 1.20 (0.78-1.86) | 0.406 | 153 (48.0) | 3.59 (2.48-5.21) | <0.001 | 1.27 (0.79-2.04) | 0.332 |
| **Political preference** (last elections) |  |  |  |  |  |  |  |  |  |  |  |
| Did not vote | 101 (27.2) | 55 (16.7) | Ref. |  | Ref. |  | 39 (12.2) | Ref. |  | Ref. |  |
| Opposition parties | 120 (32.4) | 97 (29.5) | 1.48 (0.97-2.27) | 0.068 | 1.39 (0.87-2.21) | 0.167 | 110 (34.5) | 2.37 (1.51-3.73) | <0.001 | 1.55 (0.90-2.66) | 0.112 |
| Governing parties | 150 (40.4) | 177 (53.8) | 2.17 (1.46-3.21) | <0.001 | 1.87 (1.20-2.91) | 0.006 | 170 (53.3) | 2.94 (1.91-4.51) | <0.001 | 1.55 (0.92-2.62) | 0.100 |
| **Participation at religious meetings** |  |  |  |  |  |  |  |  |  |  |  |
| At least once a month | 35 (9.4) | 44 (13.4) | Ref. |  |  |  | 46 (14.4) | Ref. |  |  |  |
| Less than once a month | 49 (13.2) | 62 (18.8) | 1.01 (0.56-1.80) | 0.983 |  |  | 71 (22.3) | 1.10 (0.62-1.95) | 0.738 |  |  |
| Never, or almost never | 287 (77.4) | 223 (67.8) | 0.62 (0.38-1.00) | 0.048 |  |  | 202 (63.3) | 0.54 (0.33-0.86) | 0.010 |  |  |
| **Contact with a close person (except children)** |  |  |  |  |  |  |  |  |  |  |  |
| Less than once a week | 37 (10.0) | 26 (7.9) | Ref. |  |  |  | 11 (3.5) | Ref. |  |  |  |
| At least once a week | 68 (18.3) | 53 (16.1) | 1.11 (0.60-2.06) | 0.742 |  |  | 43 (13.5) | 2.13 (0.98-4.61) | 0.056 |  |  |
| Daily | 266 (71.7) | 250 (76.0) | 1.34 (0.79-2.27) | 0.283 |  |  | 265 (83.0) | 3.35 (1.67-6.71) | 0.001 |  |  |
| **In conversations I consider myself a:** |  |  |  |  |  |  |  |  |  |  |  |
| *“No, but…” type* | 131 (35.3) | 80 (24.3) | Ref. |  |  |  | 66 (20.7) | Ref. |  |  |  |
| *“Yes, and…” type* | 240 (64.7) | 249 (75.7) | 1.70 (1.22-2.36) | 0.002 |  |  | 253 (79.3) | 2.09 (1.48-2.95) | <0.001 |  |  |
| **Optimism** _[4]_ |  |  |  |  |  |  |  |  |  |  |  |
| Bottom tertile | 199 (53.7) | 109 (33.1) | Ref. |  | Ref. |  | 54 (16.9) | Ref. |  | Ref. |  |
| Middle tertile | 94 (25.3) | 109 (33.1) | 2.12 (1.47-3.04) | <0.001 | 2.06 (1.38-3.08) | <0.001 | 54 (16.9) | 2.12 (1.35-3.32) | 0.001 | 1.62 (0.98-2.68) | 0.058 |
| Top tertile | 78 (21.0) | 111 (33.8) | 2.60 (1.79-3.77) | <0.001 | 2.78 (1.79-4.31) | <0.001 | 211 (66.2) | 9.97 (6.70-14.8) | <0.001 | 6.19 (3.85-9.96) | <0.001 |
| **Empathy** _[5]_ |  |  |  |  |  |  |  |  |  |  |  |
| Bottom tertile | 146 (39.3) | 115 (35.0) | Ref. |  |  |  | 75 (23.5) | Ref. |  |  |  |
| Middle tertile | 86 (23.2) | 94 (28.6) | 1.39 (0.95-2.03) | 0.092 |  |  | 106 (33.2) | 2.40 (1.61-3.57) | <0.001 |  |  |
| Top tertile | 139 (37.5) | 120 (36.4) | 1.10 (0.78-1.54) | 0.603 |  |  | 138 (43.3) | 1.93 (1.34-2.78) | <0.001 |  |  |
| **Perspective taking** _[5]_ |  |  |  |  |  |  |  |  |  |  |  |
| Bottom tertile | 168 (45.3) | 115 (35.0) | Ref. |  | Ref. |  | 71 (22.3) | Ref. |  | Ref. |  |
| Middle tertile | 81 (21.8) | 90 (27.4) | 1.62 (1.11-2.38) | 0.013 | 1.47 (0.97-2.24) | 0.072 | 91 (28.5) | 2.66 (1.77-4.00) | <0.001 | 1.96 (1.21-3.18) | 0.006 |
| Top tertile | 122 (32.9) | 124 (37.6) | 1.48 (1.05-2.10) | 0.025 | 1.16 (0.77-1.74) | 0.485 | 157 (49.2) | 3.05 (2.11-4.38) | <0.001 | 1.72 (1.09-2.72) | 0.021 |
| **Conscientiousness** _[6]_ |  |  |  |  |  |  |  |  |  |  |  |
| Bottom tertile | 151 (40.7) | 140 (42.6) | Ref. |  | Ref. |  | 66 (20.7) | Ref. |  | Ref. |  |
| Middle tertile | 102 (27.5) | 83 (25.2) | 0.88 (0.61-1.27) | 0.489 | 0.66 (0.43-1.01) | 0.058 | 109 (34.2) | 2.44 (1.65-3.63) | <0.001 | 1.20 (0.74-1.95) | 0.457 |
| Top tertile | 118 (31.8) | 106 (32.2) | 0.97 (0.68-1.37) | 0.859 | 0.57 (0.37-0.88) | 0.012 | 144 (45.1) | 2.79 (1.91-4.07) | <0.001 | 0.87 (0.53-1.42) | 0.576 |
| **Extroversion** _[6]_ |  |  |  |  |  |  |  |  |  |  |  |
| Bottom tertile | 158 (42.6) | 87 (26.4) | Ref. |  | Ref. |  | 74 (23.2) | Ref. |  | Ref. |  |
| Middle tertile | 107 (28.8) | 123 (37.4) | 2.09 (1.44-3.02) | <0.001 | 1.96 (1.31-2.93) | 0.001 | 96 (30.1) | 1.92 (1.30-2.83) | 0.001 | 1.51 (0.95-2.40) | 0.080 |
| Top tertile | 106 (28.6) | 119 (36.2) | 2.04 (1.41-2.95) | <0.001 | 1.84 (1.21-2.82) | 0.005 | 149 (46.7) | 3.00 (2.07-4.35) | <0.001 | 1.68 (1.07-2.65) | 0.025 |
| **Agreeableness** _[6]_ |  |  |  |  |  |  |  |  |  |  |  |
| Bottom tertile | 178 (48.0) | 136 (41.3) | Ref. |  | Ref. |  | 55 (17.3) | Ref. |  | Ref. |  |
| Middle tertile | 106 (28.6) | 102 (31.0) | 1.26 (0.89-1.79) | 0.199 | 1.05 (0.70-1.58) | 0.802 | 120 (37.6) | 3.66 (2.46-5.46) | <0.001 | 2.28 (1.43-3.64) | 0.001 |
| Top tertile | 87 (23.4) | 91 (27.7) | 1.37 (0.95-1.98) | 0.095 | 1.28 (0.80-2.05) | 0.299 | 144 (45.1) | 5.36 (3.58-8.01) | <0.001 | 3.17 (1.89-5.33) | <0.001 |
| **Openness** _[6]_ |  |  |  |  |  |  |  |  |  |  |  |
| Bottom tertile | 154 (41.5) | 118 (35.9) | Ref. |  |  |  | 98 (30.7) | Ref. |  |  |  |
| Middle tertile | 102 (27.5) | 98 (29.8) | 1.25 (0.87-1.81) | 0.226 |  |  | 89 (27.9) | 1.37 (0.94-2.01) | 0.104 |  |  |
| Top tertile | 115 (31.0) | 113 (34.3) | 1.28 (0.90-1.83) | 0.168 |  |  | 132 (41.4) | 1.80 (1.26-2.57) | 0.001 |  |  |
| **Neuroticism** _[6]_ |  |  |  |  |  |  |  |  |  |  |  |
| Bottom tertile | 85 (22.9) | 82 (24.9) | Ref. |  |  |  | 126 (39.5) | Ref. |  |  |  |
| Middle tertile | 54 (14.6) | 63 (19.2) | 1.21 (0.75-1.94) | 0.431 |  |  | 60 (18.8) | 0.75 (0.47-1.19) | 0.219 |  |  |
| Top tertile | 232 (62.5) | 184 (55.9) | 0.82 (0.57-1.18) | 0.286 |  |  | 133 (41.7) | 0.39 (0.27-0.55) | <0.001 |  |  |
| **COVID-19 infection (positive test)** | 27 (7.3) | 37 (11.3) | 1.61 (0.96-2.71) | 0.071 |  |  | 23 (7.2) | 0.99 (0.56-1.76) | 0.973 |  |  |
| **Approval of the COVID-19 measures implemented by the government** |  |  |  |  |  |  |  |  |  |  |  |
| No, they were unnecessary/  unjustified | 68 (18.3) | 40 (12.2) | Ref. |  |  |  | 31 (9.7) | Ref. |  |  |  |
| Yes, partially | 149 (40.2) | 122 (37.1) | 1.39 (0.88-2.20) | 0.157 |  |  | 105 (32.9) | 1.55 (0.94-2.53) | 0.083 |  |  |
| Yes, mainly or totally | 154 (41.5) | 167 (50.7) | 1.84 (1.18-2.89) | 0.007 |  |  | 183 (57.4) | 2.61 (1.62-4.19) | <0.001 |  |  |
| **Vaccinated against COVID-19** |  |  |  |  |  |  |  |  |  |  |  |
| Fully immunized (second shot or Johnson&Johnson) | 258 (69.5) | 231 (70.2) | Ref. |  | Ref. |  | 241 (75.6) | Ref. |  | Ref. |  |
| Partially immunized (first shot) | 14 (3.8) | 21 (6.4) | 1.68 (0.83-3.37) | 0.148 | 2.87 (1.29-6.37) | 0.010 | 18 (5.6) | 1.38 (0.67-2.83) | 0.385 | 4.23 (1.75-10.2) | 0.001 |
| Not yet, but made an appointment to get vaccinated | 17 (4.6) | 17 (5.2) | 1.12 (0.56-2.24) | 0.755 | 1.63 (0.76-3.53) | 0.210 | 19 (6.0) | 1.20 (0.61-2.36) | 0.604 | 2.19 (0.96-4.98) | 0.062 |
| No, won´t get vaccinated | 82 (22.1) | 60 (18.2) | 0.82 (0.56-1.19) | 0.294 | 0.99 (0.63-1.55) | 0.969 | 41 (12.8) | 0.54 (0.35-0.81) | 0.003 | 0.88 (0.52-1.50) | 0.647 |
| **BMI** [kg/m²] _[7]_ |  |  |  |  |  |  |  |  |  |  |  |
| Normal weight [BMI≥18·5 & <25] | 144 (41.9) | 130 (42.6) | Ref. |  |  |  | 135 (44.5) | Ref. |  |  |  |
| Underweight [BMI<18·5] | 18 (5.2) | 13 (4.2) | 0.80 (0.38-1.70) | 0.561 |  |  | 12 (4.0) | 0.71 (0.33-1.53) | 0.384 |  |  |
| Overweight [BMI≥25 & <30] | 102 (29.6) | 99 (32.5) | 1.08 (0.75-1.55) | 0.697 |  |  | 99 (32.7) | 1.04 (0.72-1.49) | 0.851 |  |  |
| Obesity [BMI≥30] | 80 (23.3) | 63 (20.7) | 0.87 (0.58-1.31) | 0.510 |  |  | 57 (18.8) | 0.76 (0.50-1.15) | 0.193 |  |  |
| **Frequency of physical activity done for at least 10 minutes that raises the heartbeat or respiratory rate** |  |  |  |  |  |  |  |  |  |  |  |
| Less than once a week | 90 (24.2) | 54 (16.4) | Ref. |  |  |  | 49 (15.4) | Ref. |  |  |  |
| 1-2 days a week | 91 (24.5) | 89 (27.0) | 1.63 (1.04-2.55) | 0.032 |  |  | 67 (21.0) | 1.35 (0.85-2.16) | 0.208 |  |  |
| 3-4 days a week | 90 (24.3) | 90 (27.4) | 1.67 (1.07-2.60) | 0.025 |  |  | 98 (30.7) | 2.00 (1.27-3.14) | 0.003 |  |  |
| 5-7 days a week | 100 (27.0) | 96 (29.2) | 1.60 (1.03-2.48) | 0.036 |  |  | 105 (32.9) | 1.93 (1.24-3.00) | 0.004 |  |  |
| **Smoking status** |  |  |  |  |  |  |  |  |  |  |  |
| Never | 147 (39.6) | 130 (39.5) | Ref. |  |  |  | 145 (45.5) | Ref. |  |  |  |
| Former | 104 (28.0) | 105 (31.9) | 1.14 (0.80-1.64) | 0.470 |  |  | 99 (31.0) | 0.97 (0.67-1.38) | 0.846 |  |  |
| Current | 120 (32.4) | 94 (28.6) | 0.89 (0.62-1.27) | 0.507 |  |  | 75 (23.5) | 0.63 (0.44-0.92) | 0.015 |  |  |
| **Chronic disease** _[8]_ | 147 (39.6) | 136 (41.3) | 1.07 (0.79-1.45) | 0.645 |  |  | 117 (36.7) | 0.88 (0.65-1.20) | 0.427 |  |  |
| **Depression** (ever) | 83 (22.4) | 66 (20.1) | 0.87 (0.61-1.25) | 0.456 |  |  | 36 (11.3) | 0.44 (0.29-0.67) | <0.001 |  |  |
| **Sleep problems in the last 4 weeks** ^[9]^ |  |  |  |  |  |  |  |  |  |  |  |
| None | 59 (15.9) | 78 (23.7) | Ref. |  | Ref. |  | 97 (30.4) | Ref. |  | Ref. |  |
| Once a week | 26 (7.0) | 15 (4.6) | 0.44 (0.21-0.89) | 0.024 | 0.34 (0.16-0.74) | 0.006 | 27 (8.5) | 0.63 (0.34-1.18) | 0.152 | 0.41 (0.20-0.85) | 0.017 |
| 1-2 times a week | 92 (24.8) | 67 (20.4) | 0.55 (0.35-0.87) | 0.011 | 0.45 (0.27-0.75) | 0.002 | 74 (23.2) | 0.49 (0.31-0.76) | 0.002 | 0.44 (0.26-0.75) | 0.003 |
| 3-4 times a week | 87 (23.5) | 84 (25.5) | 0.73 (0.46-1.15) | 0.173 | 0.85 (0.52-1.39) | 0.525 | 69 (21.6) | 0.48 (0.31-0.76) | 0.002 | 0.66 (0.39-1.13) | 0.132 |
| More than 5 times a week | 107 (28.8) | 85 (25.8) | 0.60 (0.39-0.93) | 0.024 | 0.77 (0.47-1.26) | 0.302 | 52 (16.3) | 0.30 (0.19-0.47) | <0.001 | 0.41 (0.24-0.72) | 0.002 |
| **Duration of sleep problems** (regarding the abovementioned) > 3 months | 206 (66.0) | 151 (60.2) | 0.78 (0.55-1.1.0) | 0.151 |  |  | 132 (59.5) | 0.75 (0.53-1.08) | 0.121 |  |  |
| **Complex real problems require the collaboration between scientists and practitioners in problem solving** |  |  |  |  |  |  |  |  |  |  |  |
| Do not agree at all or rather not agree | 48 (12.9) | 25 (7.6) | Ref. |  |  |  | 12 (3.8) | Ref. |  |  |  |
| Rather agree | 184 (49.6) | 181 (55.0) | 1.89 (1.12-3.19) | 0.018 |  |  | 140 (43.9) | 3.04 (1.56-5.95) | 0.001 |  |  |
| Agree | 139 (37.5) | 123 (37.4) | 1.70 (0.99-2.92) | 0.055 |  |  | 167 (52.3) | 4.81 (2.46-9.40) | <0.001 |  |  |
| **I have heard of the SDGs and consider them to be important** |  |  |  |  |  |  |  |  |  |  |  |
| Do not agree at all | 115 (31.0) | 78 (23.7) | Ref. |  | Ref. |  | 179 (56.1) | Ref. |  | Ref. |  |
| Rather not agree | 117 (31.6) | 85 (25.8) | 1.07 (0.72-1.60) | 0.737 | 1.11 (0.72-1.71) | 0.637 | 63 (19.8) | 1.04 (0.69-1.55) | 0.871 | 1.15 (0.71-1.87) | 0.570 |
| Rather agree | 107 (28.8) | 136 (41.4) | 1.87 (1.28-2.75) | 0.001 | 1.85 (1.22-2.81) | 0.004 | 77 (24.1) | 1.55 (1.05-2.29) | 0.029 | 1.44 (0.91-2.29) | 0.122 |
| Agree | 32 (8.6) | 30 (9.1) | 1.38 (0.78-2.46) | 0.270 | 1.35 (0.73-2.51) | 0.340 |  | 2.33 (1.37-3.96) | 0.002 | 2.04 (1.09-3.81) | 0.026 |
| **Conspiracy score** ^[10]^ |  |  |  |  |  |  |  |  |  |  |  |
| Bottom tertile | 117 (31.5) | 128 (38.9) | Ref. |  | Ref. |  | 179 (56.1) | Ref. |  | Ref. |  |
| Middle tertile | 92 (24.8) | 64 (19.5) | 0.64 (0.42-0.95) | 0.029 | 0.65 (0.40-1.02) | 0.061 | 63 (19.8) | 0.45 (0.30-0.67) | <0.001 | 0.46 (0.29-0.74) | 0.001 |
| Top tertile | 162 (43.7) | 137 (41.6) | 0.77 (0.55-1.08) | 0.136 | 0.89 (0.60-1.32) | 0.561 | 77 (24.1) | 0.31 (0.22-0.44) | <0.001 | 0.48 (0.31-0.75) | 0.001 |
| **Complexity score** ^[11]^ |  |  |  |  |  |  |  |  |  |  |  |
| Bottom tertile | 153 (41.2) | 125 (38.0) | Ref. |  |  |  | 97 (30.4) | Ref. |  |  |  |
| Middle tertile | 100 (27.0) | 85 (25.8) | 1.04 (0.72-1.51) | 0.835 |  |  | 97 (30.4) | 1.53 (1.05-2.23) | 0.027 |  |  |
| Top tertile | 118 (31.8) | 119 (36.2) | 1.23 (0.87-1.75) | 0.235 |  |  | 125 (39.2) | 1.67 (1.17-2.39) | 0.005 |  |  |
| **Weight loss** |  |  |  |  |  |  |  |  |  |  |  |
| Yes, I have tried losing weight and I lost the weight I wanted to lose | 113 (30.5) | 111 (33.8) | Ref. |  |  |  | 113 (35.4) | Ref. |  |  |  |
| Yes, I have tried losing weight but I have not lost the weight I wanted to lose | 141 (38.0) | 112 (34.0) | 0.81 (0.56-1.16) | 0.249 |  |  | 103 (32.3) | 0.73 (0.51-1.05) | 0.091 |  |  |
| Yes, I have tried losing weight but I have not lost any | 31 (8.3) | 28 (8.5) | 0.92 (0.51-1.05) | 0.775 |  |  | 19 (6.0) | 0.61 (0.32-1.15) | 0.126 |  |  |
| No, I never have tried to lose weight | 86 (23.2) | 78 (23.7) | 0.92 (0.62-1.38) | 0.698 |  |  | 84 (26.3) | 0.98 (0.66-1.45) | 0.908 |  |  |
| [1] mutually adjusted for all variables for which adjusted odds ratios with 95% confidence intervals and adjusted p-values are reported.  [2] citizenship was excluded from multivariable models due to multicollinearity  [3] TKS-WLB^1^  [4] LOT-R^2^  [5] questionnaire for empathy and perspective taking, German version^3^  [6] BFI-S^4^  [7] 67 missing values. Missing indicators were used in multivariable models.  [8] Asthma, COPD, chronical bronchitis, emphysema, heart attack, angina pectoris or coronary heart disease, cancer, hypertension, stroke or diabetes  [9] Report of difficulty initiating sleep and/or difficulty maintaining sleep and/or waking up earlier than desired.  [10] For derivation see supplementary materials  [11] For derivation see supplementary materials | | | | | | | | | | | |

References for Tables:

1 Syrek C, Bauer-Emmel C, Antoni C, Klusemann J. Entwicklung und Validierung der Trierer Kurzskala zur Messung von Work-Life Balance (TKS-WLB). *http://dx.doi.org/101026/0012-1924/a000044* 2011; **57**: 134–45.

2 Hinz A, Sander C, Glaesmer H, *et al.* Optimism and pessimism in the general population: Psychometric properties of the Life Orientation Test (LOT-R). *Int J Clin Heal Psychol* 2017; **17**: 161–70.

3 Maes, Schmitt, Schmal. Fragebogen für Empathie und Perspektivenübernahme. 1995.

4 Gerlitz J-Y, Schupp J. Research Notes Zur Erhebung der Big-Five-basierten Persönlichkeitsmerkmale im SOEP. 2014.
