# Supplementary material for: Determinants of trust in times of crises: A cross-sectional study of 3,065 German-speaking adults from the D-A-CH region
Source: PLoS One. 2023 Oct 12;18(10):e0286488. doi: 10.1371/journal.pone.0286488 (PMC10569553; doi:10.1371/journal.pone.0286488)
Supplement: S4 Table — (DOCX) [file pone.0286488.s005.docx]

| **S4 Table.** **Factors cross-sectionally associated with interpersonal trust in Germany (N=1,023).** | | | | | | | | | | | |
| --- | --- | --- | --- | --- | --- | --- | --- | --- | --- | --- | --- |
|  | Interpersonal trust | | | | | | | | | | |
|  | Lowest tertile (N=362) | Middle tertile (N=338) | | | | | Highest tertile  (N=323) | | | | |
|  | N (%) | N (%) | OR_crude_ (95% CI) | p | OR_adj._ (95% CI) ^[1]^ | p ^[1]^ | N (%) | OR_crude_ (95% CI) | p-value | OR_adj._ (95% CI) ^[1]^ | p-value ^[1]^ |
| **Age** |  |  |  |  |  |  |  |  |  |  |  |
| 18-25 | 43 (11.9) | 33 (9.7) | Ref. |  | Ref. |  | 21 (6.5) | Ref. |  | Ref. |  |
| 26-35 | 56 (15.5) | 57 (16.9) | 1.33 (0.74-2.38) | 0.344 | 1.28 (0.67-2.46) | 0.454 | 37 (11.4) | 1.35 (0.69-2.64) | 0.374 | 1.12 (0.51-2.46) | 0.782 |
| 36-45 | 51 (14.1) | 74 (21.9) | 1.89 (1.06-3.37) | 0.031 | 2.65 (1.38-5.09) | 0.003 | 51 (15.8) | 2.05 (1.07-3.92) | 0.031 | 2.05 (0.93-4.52) | 0.075 |
| 46-55 | 71 (19.6) | 52 (15.4) | 0.95 (0.54-1.70) | 0.874 | 1.27 (0.66-2.45) | 0.472 | 62 (19.2) | 1.79 (0.96-3.33) | 0.068 | 1.48 (0.69-3.15) | 0.316 |
| 56-65 | 81 (22.4) | 57 (16.9) | 0.92 (0.52-1.62) | 0.764 | 1.35 (0.70-2.61) | 0.377 | 69 (21.4) | 1.74 (0.95-3.22) | 0.075 | 1.22 (0.57-2.64) | 0.609 |
| ≥66 | 60 (16.5) | 65 (19.2) | 1.41 (0.80-2.50) | 0.239 | 2.01 (1.01-4.01) | 0.046 | 83 (25.7) | 2.83 (1.53-5.26) | 0.001 | 1.52 (0.69-3.34) | 0.298 |
| **Men** | 205 (56.6) | 175 (51.8) | Ref. |  |  |  | 151 (46.8) | Ref. |  |  |  |
| **Women** | 157 (43.4) | 163 (48.2) | 1.22 (0.90-1.64) | 0.198 |  |  | 172 (53.2) | 1.49 (1.10-2.01) | 0.010 |  |  |
| **Ethnicity** |  |  |  |  |  |  |  |  |  |  |  |
| White | 327 (90.3) | 318 (94.1) | Ref. |  | Ref. |  | 311 (96.3) | Ref. |  | Ref. |  |
| Other than white | 35 (9.7) | 20 (5.9) | 0.59 (0.33-1.04) | 0.068 | 0.64 (0.33-1.40) | 0.639 | 12 (3.7) | 0.36 (0.18-0.71) | 0.003 | 0.36 (0.16-0.82) | 0.015 |
| **Migration history** |  |  |  |  |  |  |  |  |  |  |  |
| First generation | 107 (29.6) | 125 (37.0) | Ref. |  |  |  | 112 (34.7) | Ref. |  |  |  |
| Second generation | 27 (7.5) | 25 (7.4) | 0.79 (0.43-1.45) | 0.449 |  |  | 15 (4.6) | 0.53 (0.27-1.05) | 0.070 |  |  |
| More than second generation/none | 228 (62.9) | 196 (55.6) | 0.71 (0.51-0.97) | 0.034 |  |  | 196 (60.7) | 0.82 (0.59-1.14) | 0.237 |  |  |
| **Mother tongue** |  |  |  |  |  |  |  |  |  |  |  |
| German | 336 (92.8) | 318 (94.1) | Ref. |  |  |  | 309 (95.7) | Ref. |  |  |  |
| Other than German | 26 (7.2) | 20 (5.9) | 0.81 (0.44-1.49) | 0.500 |  |  | 14 (4.3) | 0.59 (0.30-1.14) | 0.116 |  |  |
| **Living area** ^[2]^ |  |  |  |  |  |  |  |  |  |  |  |
| Urban | 231 (63.8) | 215 (63.6) | Ref. |  |  |  | 216 (66.9) | Ref. |  |  |  |
| Rural | 131 (36.2) | 123 (36.4) | 1.01 (0.74-1.37) | 0.956 |  |  | 107 (33.1) | 0.87 (0.64-1.20) | 0.401 |  |  |
| **Marital status** |  |  |  |  |  |  |  |  |  |  |  |
| Single | 113 (31.2) | 88 (26.0) | Ref. |  |  |  | 93 (28.8) | Ref. |  |  |  |
| Married/partnership | 196 (54.2) | 203 (60.1) | 1.33 (0.95-1.87) | 0.101 |  |  | 188 (58.2) | 1.17 (0.83-1.64) | 0.377 |  |  |
| Divorced | 45 (12.4) | 33 (9.8) | 0.94 (0.56-1.60) | 0.824 |  |  | 27 (8.4) | 0.73 (0.42-1.26) | 0.260 |  |  |
| Widowed | 8 (2.2) | 14 (4.1) | 2.25 (0.90-5.60) | 0.082 |  |  | 15 (4.6) | 2.28 (0.93-5.61) | 0.073 |  |  |
| **Educational attainment** |  |  |  |  |  |  |  |  |  |  |  |
| No university degree | 274 (75.7) | 223 (66.0) | Ref. |  | Ref. |  | 197 (61.0) | Ref. |  | Ref. |  |
| University degree | 88 (24.3) | 115 (34.0) | 1.61 (1.16-2.23) | 0.005 | 1.37 (0.93-1.88) | 0.111 | 126 (39.0) | 1.99 (1.43-2.77) | <0.001 | 1.67 (1.09-2.54) | 0.018 |
| **Household income** |  |  |  |  |  |  |  |  |  |  |  |
| Bottom tertile | 164 (45.3) | 114 (33.7) | Ref. |  | Ref. |  | 111 (34.4) | Ref. |  | Ref. |  |
| Middle tertile | 81 (22.4) | 69 (20.4) | 1.23 (0.82-1.83) | 0.319 | 0.90 (0.58-1.40) | 0.639 | 85 (26.3) | 1.55 (1.05-2.29) | 0.027 | 0.76 (0.47-1.22) | 0.251 |
| Highest tertile | 117 (32.3) | 155 (45.9) | 1.91 (1.36-2.67) | <0.001 | 1.25 (0.83-1.88) | 0.286 | 127 (39.3) | 1.60 (1.13-2.27) | 0.008 | 0.49 (0.31-0.79) | 0.003 |
| **Work status** |  |  |  |  |  |  |  |  |  |  |  |
| Full- (part-) time employed | 143 (39.5) | 130 (38.5) | Ref. |  |  |  | 125 (38.7) | Ref. |  |  |  |
| Full- (part-) time self-employed | 27 (7.5) | 17 (5.0) | 0.69 (0.36-1.33) | 0.269 |  |  | 18 (5.6) | 0.76 (0.40-1.45) | 0.409 |  |  |
| Unemployed | 16 (4.4) | 22 (6.5) | 1.51 (0.76-3.00) | 0.237 |  |  | 12 (3.7) | 0.86 (0.39-1.88) | 0.703 |  |  |
| Retired | 86 (24.8) | 88 (26.0) | 1.23 (0.77-1.65) | 0.542 |  |  | 95 (29.4) | 1.26 (0.87-1.84) | 0.225 |  |  |
| Student/in training/civil-/military-service | 15 (4.1) | 17 (5.0) | 1.25 (0.60-2.60) | 0.556 |  |  | 14 (4.3) | 1.07 (0.50-2.30) | 0.867 |  |  |
| Household | 22 (6.1) | 16 (4.7) | 0.80 (0.40-1.59) | 0.524 |  |  | 18 (5.6) | 0.94 (0.48-1.82) | 0.846 |  |  |
| Temporary contract | 9 (2.5) | 6 (1.8) | 0.73 (0.25-2.12) | 0.566 |  |  | 6 (1.9) | 0.76 (0.26-2.20) | 0.617 |  |  |
| Permanent contract | 44 (12.2) | 42 (12.4) | 1.05 (0.65-1.71) | 0.844 |  |  | 35 (10.8) | 0.91 (0.55-1.51) | 0.714 |  |  |
| **Satisfaction with work** |  |  |  |  |  |  |  |  |  |  |  |
| No, does not or does rather not apply | 146 (40.3) | 98 (29.0) | Ref. |  |  |  | 69 (21.4) | Ref. |  |  |  |
| Yes, does rather apply | 156 (43.1) | 178 (53.7) | 1.70 (1.22-2.37) | 0.002 |  |  | 151 (46.7) | 2.05 (1.42-2.95) | <0.001 |  |  |
| Yes, does totally apply | 60 (16.6) | 62 (18.3) | 1.54 (0.99-2.38) | 0.053 |  |  | 103 (31.9) | 3.63 (2.37-5.57) | <0.001 |  |  |
| **Work-Life balance** ^[3]^ |  |  |  |  |  |  |  |  |  |  |  |
| Bottom tertile | 150 (41.4) | 125 (37.0) | Ref. |  |  |  | 63 (19.5) | Ref. |  |  |  |
| Middle tertile | 102 (28.2) | 125 (37.0) | 1.47 (1.03-2.09) | 0.032 |  |  | 98 (30.3) | 2.29 (1.53-3.42) | <0.001 |  |  |
| Top tertile | 110 (30.4) | 88 (26.0) | 0.96 (0.66-1.39) | 0.828 |  |  | 162 (50.2) | 3.51 (2.40-5.13) | <0.001 |  |  |
| **Political preference** (last elections) |  |  |  |  |  |  |  |  |  |  |  |
| Did not vote | 98 (27.1) | 59 (17.5) | Ref. |  |  |  | 39 (12.1) | Ref. |  |  |  |
| Opposition parties | 131 (36.2) | 104 (30.7) | 1.32 (0.87-1.99) | 0.189 |  |  | 98 (30.3) | 1.88 (1.19-2.96) | 0.006 |  |  |
| Governing parties | 133 (36.7) | 175 (51.8) | 2.19 (1.47-3.24) | <0.001 |  |  | 186 (57.6) | 3.51 (2.28-5.42) | <0.001 |  |  |
| **Participation at religious meetings** |  |  |  |  |  |  |  |  |  |  |  |
| At least once a month | 37 (10.2) | 72 (21.3) | Ref. |  | Ref. |  | 47 (14.6) | Ref. |  | Ref. |  |
| Less than once a month | 39 (10.8) | 42 (12.4) | 0.55 (0.31-0.99) | 0.049 | 0.51 (0.26-0.98) | 0.043 | 46 (14.2) | 0.93 (0.51-1.70) | 0.811 | 0.64 (0.31-1.35) | 0.241 |
| Never, or almost never | 286 (79.0) | 224 (66.3) | 0.40 (0.26-0.62) | <0.001 | 0.42 (0.25-0.72) | 0.001 | 230 (71.2) | 0.63 (0.40-1.01) | 0.054 | 0.44 (0.24-0.82) | 0.009 |
| **Contact with a close person (except children)** |  |  |  |  |  |  |  |  |  |  |  |
| Less than once a week | 51 (14.1) | 54 (16.0) | Ref. |  |  |  | 24 (7.4) | Ref. |  |  |  |
| At least once a week | 63 (17.4) | 60 (17.7) | 0.90 (0.53-1.51) | 0.690 |  |  | 67 (20.8) | 2.26 (1.25-4.10) | 0.007 |  |  |
| Daily | 248 (68.5) | 224 (66.3) | 0.85 (0.56-1.30) | 0.462 |  |  | 232 (71.8) | 1.99 (1.19-3.33) | 0.009 |  |  |
| **In conversations I consider myself a:** |  |  |  |  |  |  |  |  |  |  |  |
| *“No, but…” type* | 132 (36.5) | 92 (27.2) | Ref. |  |  |  | 78 (24.2) | Ref. |  |  |  |
| *“Yes, and…” type* | 230 (63.5) | 246 (72.8) | 1.53 (1.11-2.12) |  |  |  | 245 (75.8) | 1.80 (1.29-2.51) | 0.001 |  |  |
| **Optimism** _[4]_ |  |  |  |  |  |  |  |  |  |  |  |
| Bottom tertile | 217 (59.9) | 166 (49.1) | Ref. |  | Ref. |  | 68 (21.10) | Ref. |  | Ref. |  |
| Middle tertile | 94 (26.0) | 110 (32.6) | 1.53 (1.09-2.15) | 0.015 | 1.72 (1.17-2.54) | 0.006 | 80 (24.8) | 2.72 (1.81-4.07) | <0.001 | 2.54 (1.60-4.04) | <0.001 |
| Top tertile | 51 (14.1) | 62 (18.3) | 1.59 (1.04-2.42) | 0.031 | 2.29 (1.37-3.81) | 0.001 | 175 (54.2) | 11.0 (7.23-16.6) | <0.001 | 10.6 (6.21-17.9) | <0.001 |
| **Empathy** _[5]_ |  |  |  |  |  |  |  |  |  |  |  |
| Bottom tertile | 156 (43.1) | 126 (37.3) | Ref. |  |  |  | 68 (21.1) | Ref. |  |  |  |
| Middle tertile | 83 (22.9) | 103 (30.5) | 1.54 (1.06-2.23) | 0.024 |  |  | 114 (35.3) | 3.15 (2.11-4.71) | <0.001 |  |  |
| Top tertile | 123 (34.0) | 109 (32.2) | 1.10 (0.77-1.55) | 0.602 |  |  | 141 (43.6) | 2.63 (1.81-3.82) | <0.001 |  |  |
| **Perspective taking** _[5]_ |  |  |  |  |  |  |  |  |  |  |  |
| Bottom tertile | 154 (42.5) | 120 (35.5) | Ref. |  |  |  | 71 (22.0) | Ref. |  |  |  |
| Middle tertile | 77 (21.3) | 98 (29.0) | 1.63 (1.11-2.39) | 0.012 |  |  | 89 (27.6) | 2.51 (1.66-3.80) | <0.001 |  |  |
| Top tertile | 131 (36.2) | 120 (35.5) | 1.18 (0.83-1.66) | 0.357 |  |  | 163 (50.4) | 2.70 (1.88-3.88) | <0.001 |  |  |
| **Conscientiousness** _[6]_ |  |  |  |  |  |  |  |  |  |  |  |
| Bottom tertile | 138 (38.1) | 155 (45.9) | Ref. |  | Ref. |  | 75 (23.2) | Ref. |  | Ref. |  |
| Middle tertile | 91 (25.1) | 95 (28.1) | 0.93 (0.64-1.34) | 0.697 | 0.81 (0.52-1.25) | 0.339 | 91 (28.2) | 1.84 (1.23-2.76) | 0.003 | 1.03 (0.62-1.71) | 0.909 |
| Top tertile | 133 (36.8) | 88 (26.0) | 0.59 (0.41-0.84) | 0.003 | 0.47 (0.29-0.76) | 0.002 | 157 (48.6) | 2.17 (1.51-3.13) | <0.001 | 0.71 (0.42-1.22) | 0.213 |
| **Extroversion** _[6]_ |  |  |  |  |  |  |  |  |  |  |  |
| Bottom tertile | 138 (38.1) | 84 (24.8) | Ref. |  | Ref. |  | 62 (19.2) | Ref. |  | Ref. |  |
| Middle tertile | 128 (35.4) | 158 (46.8) | 2.03 (1.42-2.90) | <0.001 | 2.02 (1.36-3.00) | <0.001 | 105 (32.5) | 1.83 (1.23-2.71) | 0.003 | 2.49 (1.55-4.01) | <0.001 |
| Top tertile | 96 (26.5) | 96 (28.4) | 1.64 (1.11-2.43) | 0.013 | 1.64 (1.05-2.55) | 0.029 | 156 (48.3) | 3.62 (2.44-5.36) | <0.001 | 2.71 (1.68-4.39) | <0.001 |
| **Agreeableness** _[6]_ |  |  |  |  |  |  |  |  |  |  |  |
| Bottom tertile | 174 (48.1) | 144 (42.6) | Ref. |  | Ref. |  | 82 (25.4) | Ref. |  | Ref. |  |
| Middle tertile | 102 (28.2) | 114 (33.7) | 1.35 (0.95-1.91) | 0.089 | 1.60 (1.07-2.40) | 0.021 | 111 (34.4) | 2.31 (1.59-3.36) | <0.001 | 1.70 (1.08-2.69) | 0.022 |
| Top tertile | 86 (23.7) | 80 (23.7) | 1.12 (0.77-1.64) | 0.542 | 1.97 (1.22-3.18) | 0.006 | 130 (40.2) | 3.21 (2.20-4.68) | <0.001 | 2.10 (1.25-3.53) | 0.005 |
| **Openness** _[6]_ |  |  |  |  |  |  |  |  |  |  |  |
| Bottom tertile | 175 (48.3) | 133 (39.3) | Ref. |  |  |  | 92 (28.5) | Ref. |  |  |  |
| Middle tertile | 99 (27.4) | 105 (31.1) | 1.40 (0.98-1.99) | 0.066 |  |  | 103 (31.9) | 1.97 (1.36-2.88) | <0.001 |  |  |
| Top tertile | 88 (24.3) | 100 (29.6) | 1.50 (1.04-2.15) | 0.031 |  |  | 128 (39.6) | 2.77 (1.91-4.01) | <0.001 |  |  |
| **Neuroticism** _[6]_ |  |  |  |  |  |  |  |  |  |  |  |
| Bottom tertile | 108 (29.8) | 67 (19.8) | Ref. |  | Ref. |  | 153 (47.4) | Ref. |  | Ref. |  |
| Middle tertile | 52 (14.4) | 64 (18.9) | 1.98 (1.23-3.19) | 0.005 | 2.33 (1.35-4.01) | 0.002 | 52 (16.1) | 0.71 (0.45-1.11) | 0.135 | 1.32 (0.74-2.35) | 0.352 |
| Top tertile | 202 (55.8) | 207 (61.3) | 1.65 (1.15-2.37) | 0.006 | 2.34 (1.51-3.63) | <0.001 | 118 (36.5) | 0.41 (0.29-0.58) | <0.001 | 1.10 (0.69-1.75) | 0.677 |
| **COVID-19 infection (positive test)** | 19 (5.3) | 23 (6.8) | 1.32 (0.70-2.47) | 0.388 |  |  | 15 (4.6) | 0.88 (0.44-1.76) | 0.716 |  |  |
| **Approval of the COVID-19 measures implemented by the government** |  |  |  |  |  |  |  |  |  |  |  |
| No, they were unnecessary/  unjustified | 76 (21.0) | 54 (16.0) | Ref. |  |  |  | 22 (6.8) | Ref. |  |  |  |
| Yes, partially | 129 (35.6) | 142 (42.0) | 1.55 (1.02-2.36) | 0.042 |  |  | 80 (24.8) | 2.14 (1.24-3.71) | 0.007 |  |  |
| Yes, mainly or totally | 157 (43.4) | 142 (42.0) | 1.27 (0.84-1.93) | 0.256 |  |  | 221 (68.4) | 4.86 (2.90-8.15) | <0.001 |  |  |
| **Vaccinated against COVID-19** |  |  |  |  |  |  |  |  |  |  |  |
| Fully immunized (second shot or Johnson&Johnson) | 221 (61.1) | 230 (68.1) | Ref. |  | Ref. |  | 254 (78.6) | Ref. |  | Ref. |  |
| Partially immunized (first shot) | 36 (9.9) | 43 (12.7) | 1.15 (0.71-1.85) | 0.574 | 1.10 (0.64-1.88) | 0.741 | 31 (9.6) | 0.75 (0.45-1.25) | 0.270 | 0.99 (0.53-1.83) | 0.965 |
| Not yet, but made an appointment to get vaccinated | 22 (6.1) | 25 (7.4) | 1.09 (0.60-1.99) | 0.775 | 1.24 (0.63-2.45) | 0.539 | 18 (5.6) | 0.71 (0.37-1.36) | 0.304 | 1.19 (0.53-2.66) | 0.668 |
| No, won´t get vaccinated | 83 (22.9) | 40 (11.8) | 0.46 (0.30-0.70) | <0.001 | 0.52 (0.32-0.85) | 0.009 | 20 (6.2) | 0.21 (0.12-0.35) | <0.001 | 0.23 (0.12-0.44) | <0.001 |
| **BMI** [kg/m²] _[7]_ |  |  |  |  |  |  |  |  |  |  |  |
| Normal weight [BMI≥18·5 & <25] | 134 (41.7) | 145 (45.2) | Ref. |  |  |  | 144 (46.7) | Ref. |  |  |  |
| Underweight [BMI<18·5] | 9 (2.8) | 8 (2.5) | 0.82 (0.31-2.19) | 0.694 |  |  | 6 (2.0) | 0.62 (0.22-1.79) | 0.377 |  |  |
| Overweight [BMI≥25 & <30] | 110 (34.3) | 108 (33.6) | 0.91 (0.64-1.29) | 0.591 |  |  | 98 (31.8) | 0.83 (0.58-1.19) | 0.307 |  |  |
| Obesity [BMI≥30] | 68 (21.2) | 60 (18.7) | 0.82 (0.54-1.24) | 0.340 |  |  | 60 (19.5) | 0.82 (0.54-1.25) | 0.357 |  |  |
| **Frequency of physical activity done for at least 10 minutes that raises the heartbeat or respiratory rate** |  |  |  |  |  |  |  |  |  |  |  |
| Less than once a week | 126 (34.8) | 79 (23.4) | Ref. |  |  |  | 69 (21.4) | Ref. |  |  |  |
| 1-2 days a week | 85 (23.5) | 102 (30.2) | 1.91 (1.28-2.86) | 0.002 |  |  | 68 (21.1) | 1.46 (0.95-2.25) | 0.002 |  |  |
| 3-4 days a week | 71 (19.6) | 95 (28.1) | 2.13 (1.41-3.24) | <0.001 |  |  | 88 (27.2) | 2.26 (1.47-3.48) | <0.001 |  |  |
| 5-7 days a week | 80 (22.1) | 62 (18.3) | 1.23 (0.80-1.91) | 0.339 |  |  | 98 (30.3) | 2.24 (1.48-3.39) | <0.001 |  |  |
| **Smoking status** |  |  |  |  |  |  |  |  |  |  |  |
| Never | 139 (38.4) | 150 (44.4) | Ref. |  |  |  | 129 (39.9) | Ref. |  |  |  |
| Former | 86 (23.8) | 89 (26.3) | 0.96 (0.66-1.40) | 0.827 |  |  | 89 (27.6) | 1.12 (0.76-1.63) | 0.575 |  |  |
| Current | 137 (37.8) | 99 (29.3) | 0.67 (0.47-0.95) | 0.023 |  |  | 105 (32.5) | 0.83 (0.58-1.17) | 0.283 |  |  |
| **Chronic disease** _[8]_ | 158 (43.7) | 145 (42.9) | 0.97 (0.72-1.31) | 0.842 |  |  | 160 (49.5) | 1.27 (0.94-1.71) | 0.123 |  |  |
| **Depression** (ever) | 75 (20.7) | 56 (16.6) | 0.76 (0.52-1.11) | 0.160 |  |  | 40 (12.4) | 0.54 (0.36-0.82) | 0.004 |  |  |
| **Sleep problems in the last 4 weeks** ^[9]^ |  |  |  |  |  |  |  |  |  |  |  |
| None | 87 (24.0) | 107 (31.7) | Ref. |  | Ref. |  | 109 (33.8) | Ref. |  | Ref. |  |
| Once a week | 9 (2.5) | 12 (3.5) | 1.08 (0.44-2.69) | 0.862 | 0.97 (0.37-2.55) | 0.954 | 25 (7.7) | 2.22 (0.98-5.00) | 0.055 | 1.81 (0.71-4.57) | 0.211 |
| 1-2 times a week | 75 (20.7) | 80 (23.7) | 0.87 (0.57-1.32) | 0.510 | 0.74 (0.47-1.18) | 0.213 | 75 (23.2) | 0.80 (0.52-1.22) | 0.300 | 0.73 (0.44-1.24) | 0.244 |
| 3-4 times a week | 97 (26.8) | 76 (22.5) | 0.64 (0.42-0.96) | 0.032 | 0.58 (0.37-0.93) | 0.022 | 67 (20.7) | 0.55 (0.36-0.84) | 0.005 | 0.56 (0.33-0.94) | 0.027 |
| More than 5 times a week | 94 (26.0) | 63 (18.6) | 0.54 (0.36-0.83) | 0.005 | 0.59 (0.37-0.95) | 0.030 | 47 (14.6) | 0.40 (0.25-0.63) | <0.001 | 0.37 (0.21-0.64) | <0.001 |
| **Duration of sleep problems** (regarding the abovementioned) > 3 months | 193 (70.2) | 133 (57.7) | 0.58 (0.40-0.83) | 0.003 |  |  | 121 (56.5) | 0.55 (0.38-0.80) | 0.002 |  |  |
| **Complex real problems require the collaboration between scientists and practitioners in problem solving** |  |  |  |  |  |  |  |  |  |  |  |
| Do not agree at all or rather not agree | 48 (13.3) | 38 (11.2) | Ref. |  |  |  | 20 (6.2) | Ref. |  |  |  |
| Rather agree | 194 (53.6) | 201 (59.5) | 1.31 (0.82-2.09) | 0.261 |  |  | 135 (41.8) | 1.67 (0.95-2.94) | 0.076 |  |  |
| Agree | 120 (33.1) | 99 (29.3) | 1.04 (0.63-1.72) | 0.872 |  |  | 168 (52.0) | 3.36 (1.90-5.95) | <0.001 |  |  |
| **I have heard of the SDGs and consider them to be important** |  |  |  |  |  |  |  |  |  |  |  |
| Do not agree at all | 97 (26.8) | 57 (16.8) | Ref. |  | Ref. |  | 55 (17.0) | Ref. |  | Ref. |  |
| Rather not agree | 121 (33.4) | 103 (30.5) | 1.45 (0.95-2.20) | 0.083 | 1.31 (0.83-2.09) | 0.250 | 72 (22.3) | 1.05 (0.68-1.63) | 0.830 | 1.38 (0.80-2.38) | 0.241 |
| Rather agree | 119 (32.9) | 143 (42.3) | 2.05 (1.36-3.07) | 0.001 | 1.64 (1.04-2.58) | 0.034 | 137 (42.4) | 2.03 (1.34-3.07) | 0.001 | 1.96 (1.17-3.26) | 0.010 |
| Agree | 25 (6.9) | 35 (10.4) | 2.38 (1.30-4.38) | 0.005 | 1.88 (0.95-3.71) | 0.070 | 59 (18.3) | 4.16 (2.35-7.38) | <0.001 | 2.81 (1.38-5.70) | 0.004 |
| **Conspiracy score** ^[10]^ |  |  |  |  |  |  |  |  |  |  |  |
| Bottom tertile | 120 (33.1) | 128 (37.9) | Ref. |  | Ref. |  | 182 (56.4) | Ref. |  | Ref. |  |
| Middle tertile | 68 (18.8) | 55 (16.3) | 0.76 (0.49-1.17) | 0.211 | 0.67 (0.42-1.09) | 0.106 | 42 (13.0) | 0.41 (0.26-0.64) | <0.001 | 0.41 (0.24-0.71) | 0.001 |
| Top tertile | 174 (48.1) | 155 (45.8) | 0.84 (0.60-1.16) | 0.285 | 0.66 (0.43-1.00) | 0.053 | 99 (30.6) | 0.38 (0.27-0.53) | <0.001 | 0.62 (0.39-0.98) | 0.040 |
| **Complexity score** ^[11]^ |  |  |  |  |  |  |  |  |  |  |  |
| Bottom tertile | 169 (46.7) | 154 (45.6) | Ref. |  | Ref. |  | 78 (24.2) | Ref. |  | Ref. |  |
| Middle tertile | 103 (28.4) | 100 (29.6) | 1.07 (0.75-1.51) | 0.724 | 0.83 (0.56-1.23) | 0.358 | 98 (30.3) | 2.06 (1.40-3.03) | <0.001 | 1.49 (0.94-2.36) | 0.090 |
| Top tertile | 90 (24.9) | 84 (24.8) | 1.02 (0.71-1.48) | 0.899 | 0.85 (0.55-1.31) | 0.464 | 147 (45.5) | 3.54 (2.43-5.15) | <0.001 | 2.41 (1.50-3.86) | <0.001 |
| **Weight loss** |  |  |  |  |  |  |  |  |  |  |  |
| Yes, I have tried losing weight and I lost the weight I wanted to lose | 94 (26.0) | 89 (26.3) | Ref. |  |  |  | 97 (30.0) | Ref. |  |  |  |
| Yes, I have tried losing weight but I have not lost the weight I wanted to lose | 114 (31.5) | 116 (34.3) | 1.07 (0.73-1.58) | 0.716 |  |  | 100 (31.0) | 0.85 (0.58-1.26) | 0.415 |  |  |
| Yes, I have tried losing weight but I have not lost any | 39 (10.8) | 35 (10.4) | 0.95 (0.55-1.63) | 0.846 |  |  | 28 (8.7) | 0.70 (0.40-1.22) | 0.206 |  |  |
| No, I never have tried to lose weight | 115 (31.7) | 98 (29.0) | 0.90 (0.61-1.34) | 0.602 |  |  | 98 (30.3) | 0.83 (0.56-1.22) | 0.338 |  |  |
| [1] mutually adjusted for all variables for which adjusted odds ratios with 95% confidence intervals and adjusted p-values are reported.  [2] citizenship was excluded from multivariable models due to multicollinearity  [3] TKS-WLB^1^  [4] LOT-R^2^  [5] questionnaire for empathy and perspective taking, German version^3^  [6] BFI-S^4^  [7] 73 missing values. Missing indicators were used in multivariable models.  [8] Asthma, COPD, chronical bronchitis, emphysema, heart attack, angina pectoris or coronary heart disease, cancer, hypertension, stroke or diabetes  [9] Report of difficulty initiating sleep and/or difficulty maintaining sleep and/or waking up earlier than desired.  [10] For derivation see supplementary materials  [11] For derivation see supplementary materials | | | | | | | | | | | |

References for Tables:

1 Syrek C, Bauer-Emmel C, Antoni C, Klusemann J. Entwicklung und Validierung der Trierer Kurzskala zur Messung von Work-Life Balance (TKS-WLB). *http://dx.doi.org/101026/0012-1924/a000044* 2011; **57**: 134–45.

2 Hinz A, Sander C, Glaesmer H, *et al.* Optimism and pessimism in the general population: Psychometric properties of the Life Orientation Test (LOT-R). *Int J Clin Heal Psychol* 2017; **17**: 161–70.

3 Maes, Schmitt, Schmal. Fragebogen für Empathie und Perspektivenübernahme. 1995.

4 Gerlitz J-Y, Schupp J. Research Notes Zur Erhebung der Big-Five-basierten Persönlichkeitsmerkmale im SOEP. 2014.
