# Supplementary material for: Determinants of trust in times of crises: A cross-sectional study of 3,065 German-speaking adults from the D-A-CH region
Source: PLoS One. 2023 Oct 12;18(10):e0286488. doi: 10.1371/journal.pone.0286488 (PMC10569553; doi:10.1371/journal.pone.0286488)
Supplement: S5 Table — (DOCX) [file pone.0286488.s006.docx]

| **S5 Table . Factors cross-sectionally associated with interpersonal trust in Switzerland (N=1,023).** | | | | | | | | | | | |
| --- | --- | --- | --- | --- | --- | --- | --- | --- | --- | --- | --- |
|  | Interpersonal trust | | | | | | | | | | |
|  | Lowest tertile (N=310) | Middle tertile (N=340) | | | | | Highest tertile  (N=373) | | | | |
|  | N (%) | N (%) | OR_crude_ (95% CI) | p | OR_adj._ (95% CI) ^[1]^ | p ^[1]^ | N (%) | OR_crude_ (95% CI) | p-value | OR_adj._ (95% CI) ^[1]^ | p-value ^[1]^ |
| **Age** |  |  |  |  |  |  |  |  |  |  |  |
| 18-25 | 34 (11.0) | 47 (13.8) | Ref. |  |  |  | 31 (8.3) | Ref. |  |  |  |
| 26-35 | 67 (21.6) | 54 (15.9) | 0.58 (0.33-1.03) | 0.063 |  |  | 49 (13.1) | 0.80 (0.44-1.48) | 0.479 |  |  |
| 36-45 | 63 (20.3) | 74 (21.8) | 0.85 (0.49-1.48) | 0.565 |  |  | 69 (18.5) | 1.20 (0.66-2.18) | 0.546 |  |  |
| 46-55 | 53 (17.1) | 59 (17.4) | 0.81 (0.45-1.43) | 0.462 |  |  | 61 (16.4) | 1.26 (0.69-2.32) | 0.454 |  |  |
| 56-65 | 60 (19.4) | 45 (13.2) | 0.54 (0.30-0.98) | 0.041 |  |  | 86 (23.1) | 1.57 (0.87-2.83) | 0.131 |  |  |
| ≥66 | 33 (10.6) | 61 (17.9) | 1.34 (0.73-2.47) | 0.352 |  |  | 77 (20.6) | 2.56 (1.36-4.83) | 0.004 |  |  |
| **Men** | 165 (53.2) | 176 (51.8) | Ref. |  |  |  | 174 (46.7) | Ref. |  |  |  |
| **Women** | 145 (46.8) | 164 (48.2) | 1.06 (0.78-1.44) | 0.709 |  |  | 199 (53.3) | 1.30 (0.96-1.76) | 0.087 |  |  |
| **Ethnicity** |  |  |  |  |  |  |  |  |  |  |  |
| White | 276 (89.0) | 297 (87.3) | Ref. |  |  |  | 344 (92.2) | Ref. |  |  |  |
| Other than white | 34 (11.0) | 43 (12.7) | 1.18 (0.73-1.90) | 0.508 |  |  | 29 (7.8) | 0.68 (0.41-1.15) | 0.153 |  |  |
| **Migration history** |  |  |  |  |  |  |  |  |  |  |  |
| First generation | 68 (21.9) | 101 (29.7) | Ref. |  | Ref. |  | 95 (25.5) | Ref. |  | Ref. |  |
| Second generation | 48 (15.5) | 46 (13.5) | 0.65 (0.39-1.07) | 0.091 | 0.71 (0.41-1.24) | 0.229 | 44 (11.8) | 0.66 (0.39-1.10) | 0.108 | 0.79 (0.43-1.44) | 0.445 |
| More than second generation/none | 194 (62.6) | 193 (56.8) | 0.67 (0.46-0.97) | 0.032 | 0.65 (0.43-0.97) | 0.037 | 234 (62.7) | 0.86 (0.60-1.24) | 0.430 | 0.69 (0.44-1.06) | 0.090 |
| **Mother tongue** |  |  |  |  |  |  |  |  |  |  |  |
| German | 271 (87.4) | 307 (90.3) | Ref. |  |  |  | 343 (92.0) | Ref. |  |  |  |
| Other than German | 39 (12.6) | 33 (9.7) | 0.75 (0.46-1.22) | 0.245 |  |  | 30 (8.0) | 0.61 (0.37-1.00) | 0.052 |  |  |
| **Living area** ^[2]^ |  |  |  |  |  |  |  |  |  |  |  |
| Urban | 129 (41.6) | 147 (43.2) | Ref. |  |  |  | 160 (42.9) | Ref. |  |  |  |
| Rural | 181 (58.4) | 193 (56.8) | 0.94 (0.69-1.28) | 0.676 |  |  | 213 (57.1) | 0.95 (0.70-1.29) | 0.736 |  |  |
| **Marital status** |  |  |  |  |  |  |  |  |  |  |  |
| Single | 114 (36.8) | 117 (34.4) | Ref. |  |  |  | 107 (28.7) | Ref. |  |  |  |
| Married/partnership | 148 (47.7) | 171 (50.3) | 1.13 (0.80-1.58) | 0.493 |  |  | 219 (58.7) | 1.58 (1.13-2.21) | 0.008 |  |  |
| Divorced | 42 (13.6) | 37 (10.9) | 0.86 (0.51-1.43) | 0.559 |  |  | 38 (10.2) | 0.96 (0.58-1.61) | 0.888 |  |  |
| Widowed | 6 (1.9) | 15 (4.4) | 2.44 (0.91-6.50) | 0.075 |  |  | 9 (2.4) | 1.60 (0.55-4.64) | 0.389 |  |  |
| **Educational attainment** |  |  |  |  |  |  |  |  |  |  |  |
| No university degree | 257 (82.9) | 283 (83.2) | Ref. |  |  |  | 283 (75.9) | Ref. |  |  |  |
| University degree | 53 (17.1) | 57 (16.8) | 0.98 (0.65-1.47) | 0.910 |  |  | 90 (24.1) | 1.54 (1.06-2.25) | 0.025 |  |  |
| **Household income** |  |  |  |  |  |  |  |  |  |  |  |
| Bottom tertile | 125 (40.3) | 124 (36.5) | Ref. |  |  |  | 93 (24.9) | Ref. |  |  |  |
| Middle tertile | 89 (28.7) | 113 (33.2) | 1.28 (0.88-1.86) | 0.194 |  |  | 114 (30.6) | 1.72 (1.17-2.53) | 0.006 |  |  |
| Highest tertile | 96 (31.0) | 103 (30.3) | 1.08 (0.74-1.57) | 0.680 |  |  | 166 (44.5) | 2.32 (1.61-3.36) | <0.001 |  |  |
| **Work status** |  |  |  |  |  |  |  |  |  |  |  |
| Full- (part-) time employed | 136 (43.9) | 148 (43.5) | Ref. |  | Ref. |  | 156 (41.8) | Ref. |  | Ref. |  |
| Full- (part-) time self-employed | 20 (6.5) | 21 (6.2) | 0.96 (0.50-1.86) | 0.915 | 1.14 (0.56-2.30) | 0.719 | 28 (7.5) | 1.22 (0.66-2.26) | 0.528 | 1.08 (0.53-2.18) | 0.839 |
| Unemployed | 18 (5.8) | 15 (4.4) | 0.77 (0.37-1.58) | 0.470 | 0.98 (0.43-2.23) | 0.970 | 8 (2.1) | 0.39 (0.16-0.92) | 0.031 | 0.56 (0.20-1.55) | 0.266 |
| Retired | 14 (4.5) | 71 (20.9) | 1.30 (0.85-2.01) | 0.225 | 1.66 (1.02-2.68) | 0.040 | 91 (24.4) | 1.59 (1.05-2.40) | 0.029 | 1.22 (0.74-2.02) | 0.429 |
| Student/in training/civil-/military-service | 21 (6.8) | 22 (6.5) | 1.44 (0.71-2.94) | 0.310 | 0.91 (0.41-2.02) | 0.817 | 22 (5.9) | 1.37 (0.67-2.78) | 0.384 | 1.08 (0.45-2.57) | 0.865 |
| Household | 8 (2.6) | 3 (0.9) | 0.96 (0.51-1.83) | 0.908 | 1.10 (0.55-2.20) | 0.788 | 20 (5.4) | 0.83 (0.43-1.60) | 0.577 | 1.03 (0.48-2.18) | 0.948 |
| Temporary contract | 43 (13.9) | 38 (11.2) | 0.34 (0.09-1.33) | 0.121 | 0.28 (0.07-1.12) | 0.072 | 9 (2.4) | 0.98 (0.37-2.62) | 0.969 | 0.79 (0.25-2.54) | 0.693 |
| Permanent contract |  |  | 0.81 (0.50-1.33) | 0.409 | 0.88 (0.52-1.51) | 0.654 | 39 (10.5) | 0.79 (0.48-1.29) | 0.348 | 0.98 (0.55-1.75) | 0.940 |
| **Satisfaction with work** |  |  |  |  |  |  |  |  |  |  |  |
| No, does not or does rather not apply | 89 (28.7) | 66 (19.4) | Ref. |  | Ref. |  | 43 (11.5) | Ref. |  | Ref. |  |
| Yes, does rather apply | 146 (47.1) | 196 (57.7) | 1.81 (1.23-2.66) | 0.002 | 1.56 (1.02-2.41) | 0.041 | 189 (50.7) | 2.68 (1.75-4.09) | <0.001 | 1.96 (1.18-3.25) | 0.010 |
| Yes, does totally apply | 75 (24.2) | 78 (22.9) | 1.40 (0.89-2.20) | 0.140 | 0.98 (0.57-1.68) | 0.945 | 141 (37.8) | 3.89 (2.46-6.16) | <0.001 | 1.53 (0.85-2.76) | 0.153 |
| **Work-Life balance** ^[3]^ |  |  |  |  |  |  |  |  |  |  |  |
| Bottom tertile | 120 (38.7) | 101 (29.7) | Ref. |  |  |  | 68 (18.2) | Ref. |  |  |  |
| Middle tertile | 98 (31.6) | 133 (39.1) | 1.61 (1.11-2.34) | 0.012 |  |  | 128 (34.3) | 2.30 (1.55-3.43) | <0.001 |  |  |
| Top tertile | 92 (20.7) | 106 (31.2) | 1.37 (0.93-2.01) | 0.110 |  |  | 177 (47.5) | 3.40 (2.30-5.01) | <0.001 |  |  |
| **Political preference** (last elections) |  |  |  |  |  |  |  |  |  |  |  |
| Did not vote | 171 (55.2) | 184 (54.1) | Ref. |  | Ref. |  | 130 (34.8) | Ref. |  | Ref. |  |
| Opposition parties | 31 (10.0) | 33 (9.7) | 0.99 (0.58-1.69) | 0.968 | 0.77 (0.43-1.38) | 0.379 | 51 (13.7) | 2.16 (1.31-3.57) | 0.003 | 1.61 (0.88-2.95) | 0.124 |
| Governing parties | 108 (34.8) | 123 (36.2) | 1.06 (0.76-1.48) | 0.737 | 0.87 (0.60-1.26) | 0.462 | 192 (51.5) | 2.34 (1.68-3.25) | <0.001 | 1.48 (1.00-2.20) | 0.050 |
| **Participation at religious meetings** |  |  |  |  |  |  |  |  |  |  |  |
| At least once a month | 39 (12.6) | 47 (13.8) | Ref. |  |  |  | 46 (12.3) | Ref. |  |  |  |
| Less than once a month | 44 (14.2) | 54 (15.9) | 1.02 (0.57-1.82) | 0.951 |  |  | 63 (16.9) | 1.21 (0.68-2.16) | 0.509 |  |  |
| Never, or almost never | 227 (73.2) | 239 (70.3) | 0.87 (0.55-1.39) | 0.566 |  |  | 264 (70.8) | 0.99 (0.62-1.57) | 0.952 |  |  |
| **Contact with a close person (except children)** |  |  |  |  |  |  |  |  |  |  |  |
| Less than once a week | 44 (14.2) | 42 (12.4) | Ref. |  |  |  | 19 (5.1) | Ref. |  |  |  |
| At least once a week | 62 (20.0) | 68 (20.0) | 1.15 (0.67-1.98) | 0.618 |  |  | 80 (21.4) | 2.99 (1.59-5.62) | <0.001 |  |  |
| Daily | 204 (65.8) | 230 (67.6) | 1.18 (0.74-1.88) | 0.481 |  |  | 274 (73.5) | 3.11 (1.76-5.49) | <0.001 |  |  |
| **In conversations I consider myself a:** |  |  |  |  |  |  |  |  |  |  |  |
| *“No, but…” type* | 117 (37.7) | 97 (28.5) | Ref. |  | Ref. |  | 71 (19.0) | Ref. |  | Ref. |  |
| *“Yes, and…” type* | 193 (62.3) | 243 (71.5) | 1.52 (1.09-2.11) | 0.013 | 1.35 (0.94-1.94) | 0.104 | 302 (81.0) | 2.58 (1.82-3.64) | <0.001 | 1.89 (1.26-2.84) | 0.002 |
| **Optimism** _[4]_ |  |  |  |  |  |  |  |  |  |  |  |
| Bottom tertile | 153 (49.3) | 128 (37.7) | Ref. |  | Ref. |  | 54 (14.5) | Ref. |  | Ref. |  |
| Middle tertile | 83 (26.8) | 104 (30.6) | 1.50 (1.03-2.17) | 0.033 | 1.29 (0.85-1.95) | 0.236 | 91 (24.4) | 3.11 (2.02-4.78) | <0.001 | 2.50 (1.53-4.08) | <0.001 |
| Top tertile | 74 (23.9) | 108 (31.7) | 1.75 (1.20-2.54) | 0.004 | 1.38 (0.87-2.14) | 0.154 | 228 (61.1) | 8.73 (5.82-13.1) | <0.001 | 5.22 (3.25-8.38) | <0.001 |
| **Empathy** _[5]_ |  |  |  |  |  |  |  |  |  |  |  |
| Bottom tertile | 104 (33.6) | 91 (26.8) | Ref. |  |  |  | 82 (22.0) | Ref. |  |  |  |
| Middle tertile | 90 (29.0) | 128 (37.6) | 1.63 (1.10-2.40) | 0.015 |  |  | 121 (32.4) | 1.71 (1.15-2.54) | 0.009 |  |  |
| Top tertile | 116 (37.4) | 121 (35.6) | 1.19 (0.82-1.74) | 0.364 |  |  | 170 (45.6) | 1.86 (1.28-2.70) | 0.001 |  |  |
| **Perspective taking** _[5]_ |  |  |  |  |  |  |  |  |  |  |  |
| Bottom tertile | 124 (40.0) | 98 (28.8) | Ref. |  | Ref. |  | 80 (21.5) | Ref. |  | Ref. |  |
| Middle tertile | 78 (25.2) | 103 (30.3) | 1.67 (1.12-2.48) | 0.011 | 1.57 (1.02-2.42) | 0.039 | 103 (27.6) | 2.05 (1.36-3.07) | 0.001 | 1.87 (1.16-3.00) | 0.010 |
| Top tertile | 108 (34.8) | 139 (40.9) | 1.63 (1.13-2.35) | 0.009 | 1.74 (1.15-2.64) | 0.009 | 190 (50.9) | 2.73 (1.89-3.94) | <0.001 | 2.26 (1.45-3.53) | <0.001 |
| **Conscientiousness** _[6]_ |  |  |  |  |  |  |  |  |  |  |  |
| Bottom tertile | 114 (36.8) | 134 (39.4) | Ref. |  | Ref. |  | 94 (25.2) | Ref. |  | Ref. |  |
| Middle tertile | 84 (27.1) | 105 (30.9) | 1.06 (0.73-1.56) | 0.751 | 0.88 (0.57-1.35) | 0.559 | 135 (36.2) | 1.95 (1.33-2.87) | 0.001 | 1.34 (0.84-2.13) | 0.224 |
| Top tertile | 112 (36.1) | 101 (29.7) | 0.77 (0.53-1.11) | 0.157 | 0.55 (0.35-0.86) | 0.008 | 144 (38.6) | 1.56 (1.08-2.25) | 0.018 | 0.81 (0.50-1.31) | 0.396 |
| **Extroversion** _[6]_ |  |  |  |  |  |  |  |  |  |  |  |
| Bottom tertile | 114 (36.8) | 104 (30.6) | Ref. |  |  |  | 95 (25.5) | Ref. |  |  |  |
| Middle tertile | 103 (33.2) | 142 (41.8) | 1.51 (1.05-2.18) | 0.028 |  |  | 117 (31.4) | 1.36 (0.93-1.99) | 0.110 |  |  |
| Top tertile | 93 (30.0) | 94 (27.6) | 1.11 (0.75-1.64) | 0.607 |  |  | 161 (42.1) | 2.08 (1.43-3.02) | <0.001 |  |  |
| **Agreeableness** _[6]_ |  |  |  |  |  |  |  |  |  |  |  |
| Bottom tertile | 139 (44.8) | 122 (35.9) | Ref. |  |  |  | 83 (22.2) | Ref. |  |  |  |
| Middle tertile | 96 (31.0) | 135 (39.7) | 1.60 (1.12-2.29) | 0.010 |  |  | 139 (37.3) | 2.42 (1.66-3.53) | <0.001 |  |  |
| Top tertile | 75 (24.2) | 83 (24.4) | 1.26 (0.85-1.87) | 0.251 |  |  | 151 (40.5) | 3.37 (2.29-4.97) | <0.001 |  |  |
| **Openness** _[6]_ |  |  |  |  |  |  |  |  |  |  |  |
| Bottom tertile | 119 (38.4) | 133 (39.1) | Ref. |  |  |  | 110 (29.5) | Ref. |  |  |  |
| Middle tertile | 83 (26.8) | 95 (27.9) | 1.02 (0.70-1.50) | 0.903 |  |  | 122 (32.7) | 1.59 (1.09-2.33) | 0.017 |  |  |
| Top tertile | 108 (34.8) | 112 (33.0) | 0.93 (0.65-1.33) | 0.685 |  |  | 141 (37.8) | 1.41 (0.98-2.03) | 0.061 |  |  |
| **Neuroticism** _[6]_ |  |  |  |  |  |  |  |  |  |  |  |
| Bottom tertile | 68 (21.9) | 76 (22.4) | Ref. |  |  |  | 156 (41.8) | Ref. |  |  |  |
| Middle tertile | 50 (16.1) | 76 (22.4) | 1.36 (0.84-2.21) | 0.213 |  |  | 72 (19.3) | 0.63 (0.40-0.99) | 0.047 |  |  |
| Top tertile | 192 (62.0) | 188 (55.2) | 0.88 (0.60-1.29) | 0.500 |  |  | 145 (38.9) | 0.33 (0.23-0.47) | <0.001 |  |  |
| **COVID-19 infection (positive test)** | 21 (6.8) | 21 (6.2) | 0.91 (0.48-1.69) | 0.757 |  |  | 30 (8.0) | 1.20 (0.67-2.14) | 0.530 |  |  |
| **Approval of the COVID-19 measures implemented by the government** |  |  |  |  |  |  |  |  |  |  |  |
| No, they were unnecessary/  unjustified | 55 (17.8) | 41 (12.0) | Ref. |  |  |  | 33 (8.8) | Ref. |  |  |  |
| Yes, partially | 116 (37.4) | 125 (36.8) | 1.45 (0.90-2.32) | 0.130 |  |  | 108 (29.0) | 1.55 (0.94-2.57) | 0.088 |  |  |
| Yes, mainly or totally | 139 (44.8) | 174 (51.2) | 1.68 (1.06-2.66) | 0.028 |  |  | 232 (62.2) | 2.78 (1.72-4.50) | <0.001 |  |  |
| **Vaccinated against COVID-19** |  |  |  |  |  |  |  |  |  |  |  |
| Fully immunized (second shot or Johnson&Johnson) | 174 (56.1) | 207 (60.9) | Ref. |  |  |  | 270 (72.4) | Ref. |  |  |  |
| Partially immunized (first shot) | 22 (7.1) | 17 (5.0) | 0.65 (0.33-1.26) | 0.203 |  |  | 13 (3.5) | 0.38 (0.19-0.78) | 0.008 |  |  |
| Not yet, but made an appointment to get vaccinated | 27 (8.7) | 21 (6.2) | 0.65 (0.36-1.20) | 0.168 |  |  | 12 (3.2) | 0.29 (0.14-0.58) | 0.001 |  |  |
| No, won´t get vaccinated | 87 (28.1) | 95 (27.9) | 0.92 (0.64-1.31) | 0.635 |  |  | 78 (20.9) | 0.58 (0.40-0.83) | 0.003 |  |  |
| **BMI** [kg/m²] _[7]_ |  |  |  |  |  |  |  |  |  |  |  |
| Normal weight [BMI≥18·5 & <25] | 136 (49.3) | 152 (50.7) | Ref. |  |  |  | 158 (45.4) | Ref. |  |  |  |
| Underweight [BMI<18·5] | 9 (3.3) | 13 (4.3) | 1.29 (0.54-3.12) | 0.568 |  |  | 8 (2.3) | 0.77 (0.29-2.04) | 0.592 |  |  |
| Overweight [BMI≥25 & <30] | 77 (27.9) | 94 (31.3) | 1.09 (0.75-1.60) | 0.649 |  |  | 117 (33.6) | 1.31 (0.91-1.89) | 0.153 |  |  |
| Obesity [BMI≥30] | 54 (19.6) | 41 (13.7) | 0.68 (0.43-1.08) | 0.105 |  |  | 65 (18.7) | 1.04 (0.68-1.59) | 0.871 |  |  |
| **Frequency of physical activity done for at least 10 minutes that raises the heartbeat or respiratory rate** |  |  |  |  |  |  |  |  |  |  |  |
| Less than once a week | 60 (19.4) | 44 (12.9) | Ref. |  | Ref. |  | 51 (13.7) | Ref. |  | Ref. |  |
| 1-2 days a week | 93 (30.0) | 89 (26.2) | 1.30 (0.80-2.12) | 0.283 | 1.32 (0.78-2.25) | 0.304 | 117 (31.4) | 1.48 (0.93-2.35) | 0.096 | 1.31 (0.75-2.28) | 0.340 |
| 3-4 days a week | 82 (26.4) | 99 (29.1) | 1.65 (1.01-2.68) | 0.045 | 1.55 (0.90-2.67) | 0.112 | 97 (26.0) | 1.39 (0.87-2.24) | 0.173 | 0.90 (0.50-1.61) | 0.719 |
| 5-7 days a week | 75 (24.2) | 108 (31.8) | 1.96 (1.21-3.20) | 0.007 | 1.78 (1.03-3.07) | 0.038 | 108 (28.9) | 1.69 ()1.05-2.73 | 0.030 | 0.91 (0.51-1.62) | 0.742 |
| **Smoking status** |  |  |  |  |  |  |  |  |  |  |  |
| Never | 99 (31.9) | 163 (47.9) | Ref. |  | Ref. |  | 180 (48.3) | Ref. |  | Ref. |  |
| Former | 79 (25.5) | 88 (25.9) | 0.68 (0.46-1.00) | 0.051 | 0.64 (0.42-0.99) | 0.043 | 100 (26.8) | 0.70 (0.47-1.02) | 0.064 | 0.65 (0.41-1.02) | 0.061 |
| Current | 132 (42.6) | 89 (26.2) | 0.41 (0.28-0.59) | <0.001 | 0.41 (0.27-0.61) | <0.001 | 93 (24.9) | 0.39 (0.27-0.56) | <0.001 | 0.40 (0.26-0.62) | <0.001 |
| **Chronic disease** _[8]_ | 116 (37.4) | 116 (34.1) | 0.87 (0.63-1.19) | 0.380 |  |  | 148 (39.7) | 1.10 (0.81-1.50) | 0.546 |  |  |
| **Depression** (ever) | 67 (21.6) | 37 (10.9) | 0.44 (0.29-0.68) | <0.001 | 0.57 (0.35-0.93) | 0.023 | 47 (12.6) | 0.52 (0.35-0.79) | 0.002 | 0.85 (0.51-1.40) | 0.521 |
| **Sleep problems in the last 4 weeks** ^[9]^ |  |  |  |  |  |  |  |  |  |  |  |
| None | 60 (19.3) | 100 (29.4) | Ref. |  | Ref. |  | 112 (30.0) | Ref. |  | Ref. |  |
| Once a week | 5 (1.6) | 22 (6.5) | 2.64 (0.95-7.34) | 0.063 | 2.74 (0.93-8.07) | 0.068 | 25 (6.7) | 2.68 (0.98-7.35) | 0.056 | 2.98 (0.96-9.20) | 0.058 |
| 1-2 times a week | 79 (25.5) | 85 (25.0) | 0.65 (0.41-1.01) | 0.053 | 0.71 (0.44-1.14) | 0.157 | 84 (22.5) | 0.57 (0.37-0.88) | 0.012 | 0.73 (0.44-1.22) | 0.232 |
| 3-4 times a week | 88 (28.4) | 71 (20.9) | 0.48 (0.31-0.76) | 0.001 | 0.59 (0.36-0.95) | 0.031 | 91 (24.4) | 0.55 (0.36-0.85) | 0.007 | 0.77 (0.47-1.28) | 0.313 |
| More than 5 times a week | 78 (25.2) | 62 (18.2) | 0.48 (0.30-0.76) | 0.002 | 0.56 (0.34-0.93) | 0.025 | 61 (16.4) | 0.42 (0.26-0.66) | <0.001 | 0.60 (0.35-1.03) | 0.062 |
| **Duration of sleep problems** (regarding the abovementioned) > 3 months | 170 (68.0) | 141 (58.8) | 0.67 (0.46-0.97) | 0.034 |  |  | 173 (66.3) | 0.93 (0.64-1.34) | 0.680 |  |  |
| **Complex real problems require the collaboration between scientists and practitioners in problem solving** |  |  |  |  |  |  |  |  |  |  |  |
| Do not agree at all or rather not agree | 55 (17.7) | 41 (12.1) | Ref. |  | Ref. |  | 15 (4.0) | Ref. |  | Ref. |  |
| Rather agree | 160 (51.6) | 200 (58.8) | 1.68 (1.06-2.64) | 0.026 | 1.45 (0.87-2.41) | 0.155 | 199 (53.4) | 4.56 (2.48-8.37) | <0.001 | 2.45 (1.23-4.85) | 0.011 |
| Agree | 95 (30.7) | 99 (29.1) | 1.40 (0.85-2.29) | 0.183 | 1.22 (0.68-2.17) | 0.508 | 159 (42.6) | 6.14 (3.29-11.5) | <0.001 | 2.30 (1.11-4.77) | 0.025 |
| **I have heard of the SDGs and consider them to be important** |  |  |  |  |  |  |  |  |  |  |  |
| Do not agree at all | 70 (22.6) | 64 (18.8) | Ref. |  | Ref. |  | 93 (24.9) | Ref. |  | Ref. |  |
| Rather not agree | 109 (35.2) | 121 (35.6) | 1.21 (0.79-1.86) | 0.373 | 1.01 (0.64-1.63) | 0.952 | 85 (22.8) | 0.59 (0.39-0.89) | 0.013 | 0.56 (0.34-0.92) | 0.022 |
| Rather agree | 113 (36.4) | 130 (38.2) | 1.26 (0.82-1.92) | 0.286 | 0.94 (0.59-1.50) | 0.795 | 136 (36.5) | 0.91 (0.61-1.35) | 0.626 | 0.71 (0.44-1.14) | 0.160 |
| Agree | 18 (5.8) | 25 (7.4) | 1.52 (0.76-3.04) | 0.238 | 1.56 (0.72-3.38) | 0.256 | 59 (15.8) | 2.47 (1.34-4.55) | 0.004 | 2.18 (1.04-4.58) | 0.040 |
| **Conspiracy score** ^[10]^ |  |  |  |  |  |  |  |  |  |  |  |
| Bottom tertile | 83 (26.7) | 117 (34.4) | Ref. |  | Ref. |  | 194 (52.0) | Ref. |  | Ref. |  |
| Middle tertile | 52 (16.7) | 60 (17.7) | 0.82 (0.51-1.30) | 0.400 | 0.87 (0.53-1.44) | 0.595 | 71 (19.0) | 0.58 (0.38-0.91) | 0.017 | 0.79 (0.48-1.31) | 0.364 |
| Top tertile | 175 (56.4) | 163 (47.9) | 0.66 (0.46-0.94) | 0.021 | 0.70 (0.47-1.04) | 0.076 | 108 (29.0) | 0.26 (0.19-0.38) | <0.001 | 0.39 (0.26-0.59) | <0.001 |
| **Complexity score ^[11]^** |  |  |  |  |  |  |  |  |  |  |  |
| Bottom tertile | 144 (46.5) | 151 (44.4) | Ref. |  |  |  | 127 (34.1) | Ref. |  |  |  |
| Middle tertile | 86 (27.7) | 107 (31.5) | 1.19 (0.82-1.71) | 0.357 |  |  | 115 (30.8) | 1.52 (1.05-2.19) | 0.026 |  |  |
| Top tertile | 80 (25.8) | 82 (24.1) | 0.98 (0.67-1.43) | 0.907 |  |  | 131 (35.1) | 1.86 (1.29-2.68) | 0.001 |  |  |
| **Weight loss** |  |  |  |  |  |  |  |  |  |  |  |
| Yes, I have tried losing weight and I lost the weight I wanted to lose | 99 (31.9) | 107 (31.5) | Ref.1. |  |  |  | 130 (34.9) | Ref. |  |  |  |
| Yes, I have tried losing weight but I have not lost the weight I wanted to lose | 100 (32.3) | 105 (30.9) | 0.97 (0.66-1.43) | 0.884 |  |  | 112 (30.0) | 0.85 (0.59-1.24) | 0.406 |  |  |
| Yes, I have tried losing weight but I have not lost any | 30 (9.7) | 26 (7.6) | 0.80 (0.44-1.45) | 0.465 |  |  | 32 (8.6) | 0.81 (0.46-1.43) | 0.469 |  |  |
| No, I never have tried to lose weight | 81 (26.1) | 102 (30.0) | 1.17 (0.78-1.74) | 0.454 |  |  | 99 (26.5) | 0.93 (0.63-1.38) | 0.721 |  |  |
| [1] mutually adjusted for all variables for which adjusted odds ratios with 95% confidence intervals and adjusted p-values are reported.  [2] citizenship was excluded from multivariable models due to multicollinearity  [3] TKS-WLB^1^  [4] LOT-R^2^  [5] questionnaire for empathy and perspective taking, German version^3^  [6] BFI-S^4^  [7] 99 missing values. Missing indicators were used in multivariable models.  [8] Asthma, COPD, chronical bronchitis, emphysema, heart attack, angina pectoris or coronary heart disease, cancer, hypertension, stroke or diabetes  [9] Report of difficulty initiating sleep and/or difficulty maintaining sleep and/or waking up earlier than desired.  [10] For derivation see supplementary materials  [11] For derivation see supplementary materials | | | | | | | | | | | |

References for Tables:

1 Syrek C, Bauer-Emmel C, Antoni C, Klusemann J. Entwicklung und Validierung der Trierer Kurzskala zur Messung von Work-Life Balance (TKS-WLB). *http://dx.doi.org/101026/0012-1924/a000044* 2011; **57**: 134–45.

2 Hinz A, Sander C, Glaesmer H, *et al.* Optimism and pessimism in the general population: Psychometric properties of the Life Orientation Test (LOT-R). *Int J Clin Heal Psychol* 2017; **17**: 161–70.

3 Maes, Schmitt, Schmal. Fragebogen für Empathie und Perspektivenübernahme. 1995.

4 Gerlitz J-Y, Schupp J. Research Notes Zur Erhebung der Big-Five-basierten Persönlichkeitsmerkmale im SOEP. 2014.
